# Fluorescence *In Situ* Hybridization and Optical Mapping to Correct Scaffold Arrangement in the Tomato Genome

**DOI:** 10.1534/g3.114.011197

**Published:** 2014-05-30

**Authors:** Lindsay A. Shearer, Lorinda K. Anderson, Hans de Jong, Sandra Smit, José Luis Goicoechea, Bruce A. Roe, Axin Hua, James J. Giovannoni, Stephen M. Stack

**Affiliations:** * Department of Biology, Colorado State University, Fort Collins, Colorado 80523; † Laboratory of Genetics, Wageningen University and Research Centre (WUR), Droevendaalsesteeg 1, 6708 PB Wageningen, The Netherlands; ‡ Laboratory of Bioinformatics, WUR, Droevendaalsesteeg1, 6708 PB Wageningen, The Netherlands; § Arizona Genomics Institute, University of Arizona, Tucson, Arizona 85721; ** Department of Chemistry and Biochemistry, Stephenson Research and Technology Center, University of Oklahoma, Norman, Oklahoma 73019; †† Department of Plant Biology, Cornell University, Ithaca, New York 14853

**Keywords:** tomato, genome, scaffolds, fluorescence *in situ* hybridization, optical mapping

## Abstract

The order and orientation (arrangement) of all 91 sequenced scaffolds in the 12 pseudomolecules of the recently published tomato (*Solanum lycopersicum*, 2*n *= 2*x =* 24) genome sequence were positioned based on marker order in a high-density linkage map. Here, we report the arrangement of these scaffolds determined by two independent physical methods, bacterial artificial chromosome–fluorescence *in situ* hybridization (BAC-FISH) and optical mapping. By localizing BACs at the ends of scaffolds to spreads of tomato synaptonemal complexes (pachytene chromosomes), we showed that 45 scaffolds, representing one-third of the tomato genome, were arranged differently than predicted by the linkage map. These scaffolds occur mostly in pericentric heterochromatin where 77% of the tomato genome is located and where linkage mapping is less accurate due to reduced crossing over. Although useful for only part of the genome, optical mapping results were in complete agreement with scaffold arrangement by FISH but often disagreed with scaffold arrangement based on the linkage map. The scaffold arrangement based on FISH and optical mapping changes the positions of hundreds of markers in the linkage map, especially in heterochromatin. These results suggest that similar errors exist in pseudomolecules from other large genomes that have been assembled using only linkage maps to predict scaffold arrangement, and these errors can be corrected using FISH and/or optical mapping. Of note, BAC-FISH also permits estimates of the sizes of gaps between scaffolds, and unanchored BACs are often visualized by FISH in gaps between scaffolds and thus represent starting points for filling these gaps.

The recently sequenced tomato (*Solanum lycopersicum*, 2*n* = 2*x* = 24) genome consists of 12 DNA pseudomolecules corresponding to the 12 tomato chromosomes (The Tomato Genome Consortium 2012). Each pseudomolecule is a linear series of sequenced DNA scaffolds interrupted by gaps of unknown size. Ideally, the arrangement (order and orientation) of scaffolds from the head of a pseudomolecule (end of the short arm of a chromosome, starting with telomere sequence) to the tail of the pseudomolecule (the end of the long arm of a chromosome, ending with telomere sequence) is supposed to be the same as the DNA double helix that runs the length of the corresponding chromosome.

Two methods were used to arrange scaffolds in tomato pseudomolecules (The Tomato Genome Consortium 2012). One relies on identifying mapped molecular markers in scaffolds and then ordering and orienting scaffolds according to the locations of these markers in a high-resolution linkage map. The other relies on fluorescence *in situ* hybridization (FISH) of bacterial artificial chromosomes (BACs) to localize scaffold DNA to spreads of synaptonemal complexes (SCs = pachytene chromosomes) and then ordering and orienting the scaffolds according to the arrangement of the fluorescent signals on SCs. At the time of publication, disagreements in scaffold arrangements based on the two methods were resolved in favor of the Kazusa EXPEN 2000 linkage map. This was appropriate because most of the needed FISH localizations were not yet available. However, now the FISH localizations have been completed, and we find that FISH and the linkage map scaffold arrangements disagree for one-third of the genome. Most of the discrepancies occur in pericentric heterochromatin that includes 77% of the tomato genome and approximately 10% of tomato’s estimated 35,000 nuclear genes (Peterson *et al.* 1996; Van der Hoeven *et al.* 2002; Wang *et al.* 2006b; Peters *et al.* 2009; The Tomato Genome Consortium 2012). It is significant that when scaffolds are arranged using an independent physical method called optical mapping (Dong *et al.* 2013), the results are completely compatible with FISH-based arrangements, while often contradicting linkage map-based arrangements. FISH and optical mapping are based on actual visualization of relative sequence locations, and thus are more likely to reflect biological reality than linkage mapping (Peterson 2014). Consequently, these results suggest that similar problems are likely to occur in other genomes where scaffold arrangements were based exclusively on linkage maps.

## Materials and Methods

### Tomato plants

*Solanum lycopersicum*, var. *Cherry*, accession LA4444, and *S. lycopersicum*, var. Heinz 1706, and reciprocal hybrids of these two lines were grown from seeds to flowering in a greenhouse. Even though var. Heinz 1706 was used for sequencing, almost all BAC-FISH were performed on accession LA4444 because of its characteristics of indeterminate growth and abundant flowering.

### Spreading tomato SCs for FISH and electron microscopy

Tomato SC spreads were prepared as described previously (Stack *et al.* 2009; Stack and Anderson 2009). Briefly, primary microsporocytes in pachytene were squeezed out of anthers, and cell walls were removed with cytohelicase. The resulting protoplast suspension was mixed with an aqueous hypotonic bursting medium [0.05% v/v IGEPAL CA-630 (Sigma), 0.3% w/v (para)formaldehyde, and 0.001% w/v potassium dextran sulfate] and placed on either a glow-discharged glass microscope slide for FISH or a glow-discharged plastic-coated slide for electron microscopy (EM). Slides were sprayed with aqueous 4% w/v (para)formaldehyde, air-dried, washed briefly in deionized water, air-dried again, and then stored up to 2 yr in sealed boxes at −80° for later FISH.

### Probes for FISH

To determine which BACs to use for locating the ends of scaffolds by FISH, it was first necessary to determine the order of BAC clones in scaffolds. We used clone-end sequences [T7 and SP6 and 26-nucleotide sequence tags (http://solgenomics.net)] from the Whole Genome Profiling physical map of tomato (Van Oeveren *et al.* 2011) to map BACs to the genome assembly using BLAST and SOAP (Altschul *et al.* 1990; Li *et al.* 2008). We also wrote several custom scripts to parse the mapping results and determine reliable BAC locations in the scaffolds (Supporting Information, Table S1). Once the order of BAC clones in scaffolds was determined, BACs with sequence at or near the head (toward the end of the short arm of the chromosome) and tail (toward the end of the long arm) of every scaffold were selected to use as probes. Probes were prepared from the tomato *Hind*III, *Mbo*I, *Eco*RI, sheared BAC, and fosmid libraries located at Cornell University (http://solgenomics.net/). Bacteria were grown by standard protocols. BACs and fosmids were isolated using the plasmid kit from AquaPlasmid (MultiTarget Pharmaceuticals, Salt Lake City, Utah) with modifications of the manufacturer’s instructions for BACs and fosmids (File S1). Isolated DNAs were labeled with digoxygenin, biotin, or dinitrophenol (DNP) using a nick translation kit according to the manufacturer’s instructions (Roche Applied Science), except digestion time was reduced from the recommended 2 hr to 0.5 hr or 1 hr. With only four exceptions, each end of each scaffold was marked by hybridization of one BAC, which showed the head–tail orientation and location of each scaffold on an SC. The four exceptions were scaffolds shorter than 400 kb, where only one BAC per scaffold was localized to show the position but not the orientation of these scaffolds on SCs. To determine the relative positions of adjacent scaffolds and the gap sizes between them, two BACs, one each from the two scaffold ends facing the gap, were hybridized at the same time. In some cases, four BACs marking the four ends of two adjacent scaffolds were hybridized simultaneously.

### Fluorescence *in situ* hybridization

FISH was performed as described by (Zhong *et al.* 1996; Chang *et al.* 2007). Briefly, air-dried slides were scanned by phase microscopy, good SC spreads were imaged, and their microscope stage coordinates were recorded. The slides were incubated in 45% v/v acetic acid for 1 min, fixed with 1:3 acetic ethanol for 1 min, and then digested with RNase followed by pepsin. After additional fixation in 1% w/v (para)formaldehyde, 20 µl of hybridization mixture [aqueous 2×SSC that was 50% (v/v) formamide, 10% (w/v) sodium dextran sulfate, 0.25% (w/v) sodium dodecyl sulfate with 50–1000 ng of one or more labeled probes and 1–5 µg of unlabeled Cot 100 tomato DNA to block repeated sequences] was placed on each slide, and a cover glass was added. Slides were incubated at 80° on an aluminum block for 2.5 min to denature the DNA, and then slides were incubated at 37° for at least 12 hr to permit hybridization. Slides were then washed three times in aqueous 2×SSC that was 50% v/v formamide at 42° for 80% stringency (Schwarzacher and Heslop-Harrison 2000). Blocking and antibody incubations were performed at 37° in 1-hr increments with three 3-min washes in a solution of 100 mM Tris, 150 mM NaCl, and 0.05% v/v Tween-20, pH 7.5, after each incubation step. Antibodies (from Jackson ImmunoResearch Laboratories except when otherwise indicated) included mouse anti-biotin (1:100), biotinylated donkey anti-mouse (1:125), rat anti-DNP (1:100, Invitrogen), sheep anti-digoxigenin conjugated to tetramethyl rhodamine isothiocyanate (TRITC; 1:100, Roche), donkey anti-rat conjugated to Dylight 649 (1:100), streptavidin conjugated to fluorescein isothiocyanate (FITC; 1:200), and donkey anti-sheep conjugated to TRITC (1:100). Donkey serum (5%) was added to the blocking buffer when appropriate. After immunolabeling, slides were dehydrated through an ethanol series and air-dried. Cover glasses were mounted with Vectashield (Vector Laboratories) containing 5 µg/ml 4′,6-diamidino-2-phenylindole (DAPI).

### Microscopy

Microscopy and photography were performed with Leica DM 5000B and DM 5500B microscopes, both equipped for phase contrast and fluorescence microscopy with DAPI, FITC, TRITC, and Cy5 (to detect Dylight 649 fluorophore) filter cubes and zero pixel shift. Images were captured with cooled Hamamatsu monochrome 1344×1044 pixel cameras using IP Lab software (version 4).

### Measuring positions of BACs on SCs

After hybridization and immunolabeling, SC spreads were located and photographed using filter cubes appropriate for the fluorescent probes. Pseudocolored fluorescent images were overlaid on corresponding phase images to mark sites of hybridization. The location of each BAC was determined as a percentage of arm length from the center of the kinetochore on at least 10 different SC spreads, and the percentiles were averaged. To express BAC location in micrometers, the percentage of the arm location was multiplied by the average length of the SC arm (Sherman and Stack 1992; Peterson *et al.* 1996; Chang *et al.* 2007). Seven of the 12 SCs were identified reliably using relative length and arm ratio, but within SC group *7*, *9*, and *10* and within SC group *5* and *12*, SCs were indistinguishable by light microcopy. When a BAC FISHed to one of these SCs, the BAC in question was FISHed again, along with appropriate marker BACs to verify the SC and arm involved.

### Determining linear DNA density on SCs

The length in micrometers of a scaffold on an SC can be determined by measuring the distance between FISH foci on the scaffold’s borders. This length divided by the megabases in the scaffold provides an estimate of the amount of DNA per chromatid per micrometer of SC in the type of chromatin involved (the linear density of DNA). However, a correction is required because the amount of DNA between the FISH signals is an overestimate. To explain, the location of a signal is considered to be at the center of a BAC so the amount of DNA between the BAC signals on the borders of the scaffold should be the scaffold size minus half the DNA in the left border BAC and minus half of the DNA in the right border BAC. In addition, sometimes the border BACs were unsuitable for FISH, so a more proximal BAC had to be used. In this case, all of the DNA distal to the BAC signal needs to be subtracted from the scaffold size to determine the amount of DNA between the BAC signals. Knowing the distance in micrometers between BAC signals on the borders of a scaffold, the corrected amount of DNA between the BAC signals, and the type of chromatin between the signals, the linear densities of kinetochores (centromeres), euchromatin, and heterochromatin were estimated (see File S1 for further explanation).

### Determining gap sizes between adjacent scaffolds

The amount of DNA in each gap was estimated by first measuring the distance in micrometers between the two fluorescent foci on the borders of the gap. This distance was multiplied by the linear DNA density of the chromatin type involved to give the amount of DNA between the signals. However, again, a correction is required because the amount of DNA between the FISH signals is an overestimate of the size of the gap (see above and File S1)

Larger gaps between foci could be measured more accurately than smaller gaps due to the resolution of light microscopy. In some cases, the two BAC foci on adjacent scaffolds were too close to each other to measure with confidence, so the gap was arbitrarily assigned a default length of 0.1 µm (just below the resolution of the light microscope). In such small gaps, subtracting scaffold DNA extending toward the gaps from the amount of DNA calculated between the FISH signals sometimes resulted in a negative value for the gap size. Negative values were recorded as 0 kb to indicate very small gaps at or below the resolution of FISH.

### Optical mapping

DNA was isolated from 100 g of expanding leaves of Heinz 1706 seedlings harvested 21 d after planting, with the final 3 d of growth in the dark to reduce starch content. High-molecular-weight DNA was prepared by first isolating nuclei, embedding them in agarose, and then lysing the nuclei while in agarose to reduce shearing as described by Zhang *et al.* (1994). Optical mapping was performed by OpGen, Inc. using the protocol previously described for other plant genomes (Zhou *et al.* 2009; Young *et al.* 2011; Dong *et al.* 2013; Chamala *et al.* 2013). Briefly, this entailed mounting the genomic DNA (∼400–500 kb) onto derivatized glass surfaces using a silastic micro channel system (Dimalanta *et al.* 2004). After digesting the immobilized DNA with *Bam*H1, the linear strands were imaged by a charge-coupled device (CCD) camera at OpGen. The restriction fragment sizes were determined by measuring the distances between the visualized gaps caused by DNA cleavage with the restriction enzyme. The OpGen Mapper software was used to analyze the images channel-by-channel. Nonlinear distorted and short fragments were filtered out prior to generating an ordered restriction map for each genomic DNA molecule. Details of these procedures were previously described (Dimalanta *et al.* 2004; Zhou *et al.* 2004, 2007a). When optical mapping spanned gaps between adjacent sequenced scaffolds, it was possible to determine the order and orientation of the scaffolds relative to each other and to estimate the sizes of their included gaps.

### Electron microscopy of SC spreads

SCs on plastic coated slides were prepared as described by Stack and Anderson (2009). Briefly, SC spreads were digested with DNase I and then fixed in a combination of 2% formaldehyde and 2% glutaraldehyde. After washing, SCs were stained in alcoholic phosphotungstic acid, washed, and dried. Copper EM grids were placed over SC spreads. The grids and plastic film were lifted from the slides and air-dried. SC spreads on grids were examined and photographed using a JEOL 2000 electron microscope.

## Results

### BAC-FISH localizations

**Figure 1:**
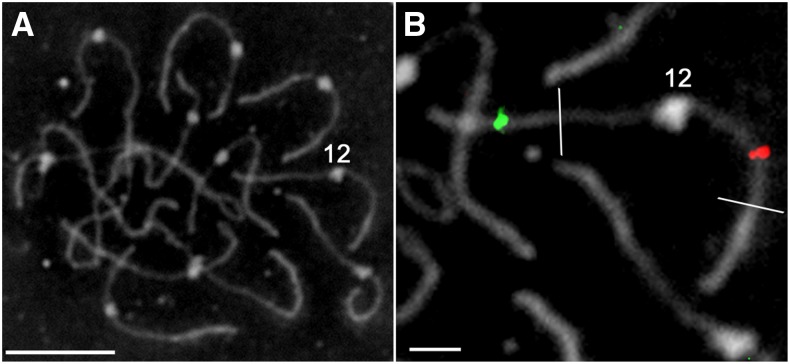
BAC-FISH on tomato SC spreads is effective in both euchromatin and heterochromatin. (A) Digitally reversed, phase contrast image of a complete set of tomato SCs (pachytene chromosomes). Kinetochores appear as fuzzy white disks/ellipses approximately 1 μm in diameter on the SCs. SC in distal euchromatin is relatively thick compared with SC in proximal pericentric heterochromatin. The kinetochore of SC *12* is marked “12.” (B) Enlarged, digitally reversed, phase image of the same SC *12* illustrated in (A) showing FISH localization of two BACs. The approximate borders between distal euchromatin and pericentric heterochromatin are marked with transverse white lines in each arm. The location of BAC LE_HBa0017P17 is indicated by a green focus in distal euchromatin of the long arm, and the location of BAC SL_*Mbo*I0038L04 is indicated by a red focus in pericentric heterochromatin of the short arm. The bar in (A) represents 10 µm, and the bar in (B) represents 2 µm.

For most individual BACs, FISH on SC spreads resulted in a single, discrete site of hybridization (Figure 1). BAC positions were mapped with a high degree of accuracy because there is little or no distortion of SCs in spreads and because BAC locations were averages of measurements from 10 or more different SC spreads. In addition, blocking repeated sequences during hybridization made BAC localizations in heterochromatin often as unique as localizations in euchromatin. Using this technique, we localized 627 BACs to unique sites on tomato SCs (Figure S1, Table S2 and Table S3) (see http://solgenomics.net/cview/map.pl?map_version_id=25 for identification of every BAC on the idiogram and supporting FISH images). An additional 12 BACs localized to two sites, either on the same SC (five BACS) or to two different SCs (seven BACs) [Figure S2 and Figure 8 in (Stack *et al.* 2009)].

We also determined the locations of certain repeated sequences on SCs (Figure S1). Telomeric sequence was observed at the ends of every chromosome, mitochondrial DNA at a distinct site on the long arm of chromosome *11*, 45S rDNA on the distal half of the short arm of chromosome *2*, and 5S rDNA near the kinetochore on the short arm of chromosome *1*. We also found several BACs (*e.g.*, SL_*Mbo*I0034IOA, *Mbo*I034I08, *Eco*RI001K05, *Hin*dIII074704) that all hybridize to the same sites on chromosomes *1*, *2*, *3*, *6*, *8*, *11*, as well as throughout the nucleolar organizer region (NOR) on chromosome *2*. This hybridization pattern is probably due to a satellite repeat consisting of 45S rDNA intergenic spacer sequence (Jo *et al.* 2009). All of these sites are in heterochromatin, and many are located in gaps between scaffolds.

### Ordering and orienting scaffolds in pseudomolecules by FISH

**Figure 2:**
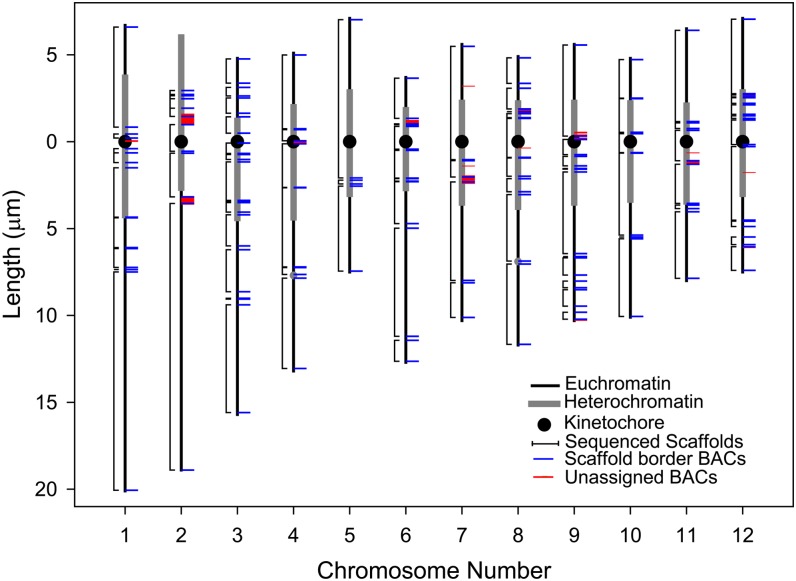
Idiogram of the 12 tomato pachytene SCs (bivalent chromosomes) with FISH localizations of selected BACs. SCs are represented by vertical lines with thinner black segments in distal euchromatin and thicker gray segments in pericentric heterochromatin. The distal half of the short arm of SC *2* is the heterochromatic nucleolus organizer. Kinetochores (at centromeres) are represented by black disks, and gray dots on SCs *4* and *8* represent chromomeres. Horizontal blue lines are localization sites of BACs at or near the ends of scaffolds (Table S4). Brackets to the left of each SC show the chromosomal locations and boundaries of scaffolds. Spaces between brackets are gaps in sequencing and/or assembly between scaffolds. Some scaffolds are so small that they appear only as lines in gaps, and some gaps are so small that no space is visible between adjacent brackets. Horizontal red lines are localization sites of BACs that are not assigned to any of the 12 pseudomolecules, *i.e.*, chromosome *0* BACs (Table S10). Note that most, but not all, unassigned BACs localize to gaps between scaffolds.

**Figure 3:**
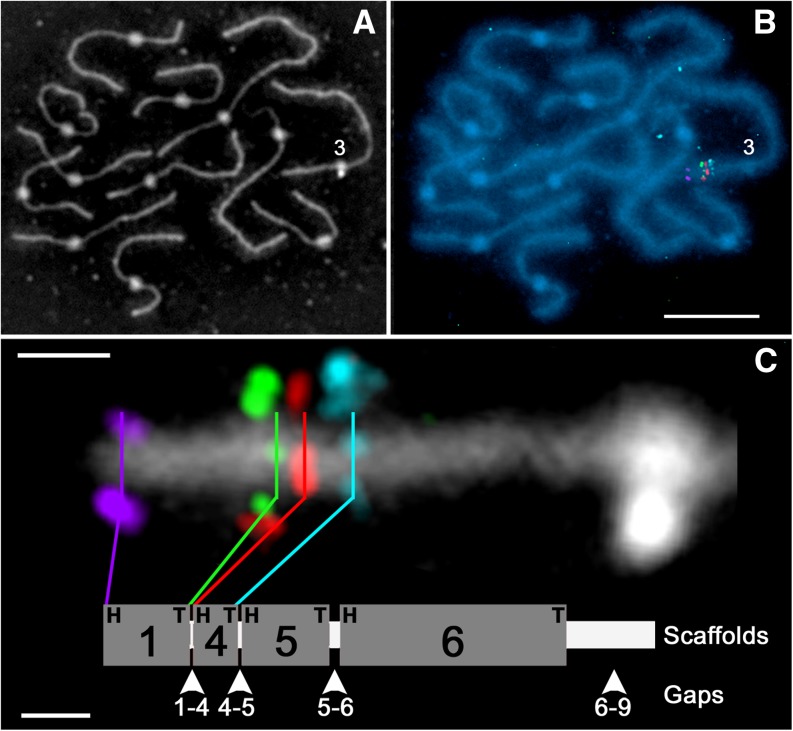
Representative example of simultaneous FISH localization of four BACs to define the borders of two scaffolds and the gap between them on SC *3*. Because scaffold numbering is based on the linkage map, the two adjacent scaffolds are numbered 1 and 4, whereas scaffolds 2 and 3 were localized by FISH to positions on the long arm of chromosome *3* (see Figure 4). (A) Reversed phase image of a complete SC set with SC *3* marked “3” at its kinetochore. (B) Fluorescent image of the same SC set in (A) with DNA stained blue with DAPI and showing colored foci that are FISH localizations of BACs in the distal euchromatin of the short arm of SC *3*. (C) Enlarged reversed phase contrast image of the short arm of SC *3* with the BAC-FISH localizations shown in (B). The upper lobe of the white, dumbbell-shaped structure to the right is the kinetochore, while the lower lobe is debris visible by phase contrast microscopy. BAC SL_s0009C01 (purple) is at the head (H = toward the end of the short arm) and BAC SL_s0086D22 (green) is at the tail (T = toward the end of the long arm) of scaffold 1 (SL2.40sc04439). BAC SL_s0018K15 (red) is at the head and BAC SL_s0002G24 (turquoise) is at the tail of the adjacent scaffold 4 (SL2.40sc4696). Scaffolds 1 and 4 are in distal euchromatin. The space between the green signals and the red signals is the gap between scaffolds 1 and 4. The purple and the turquoise foci mark the location of DNP-labeled BAC probes that were the same color in the original image but that have been given different pseudo colors here. In the diagram beneath the SC, the thick gray segments labeled 1, 4, 5, and 6 represent scaffolds SL2.40sc04439, SL2.40sc4696, SL2.40sc05330, and SL2.40sc4126, respectively, with their lengths proportional to the amounts of DNA they represent (Table S5). BAC-FISH localizations used to order and orient scaffolds 5 and 6 are not illustrated. Based on FISH, these scaffolds have the same head–tail orientation, but a different order from that derived from the linkage map (Figure 4 and Table S4). Gaps between scaffolds are named according to the scaffolds on either side, *e.g.*, 1-4, 4-5, *etc*., and gap lengths (white lines between the scaffolds) are proportional to the amount of DNA they are estimated to represent (Table S7). The bar in (B) represents 10 µm for (A) and (B). In (C), the upper bar represents 1 µm in reference to the SC segment, while the lower bar represents 2 Mb in reference to the pseudomolecule.

**Figure 4:**
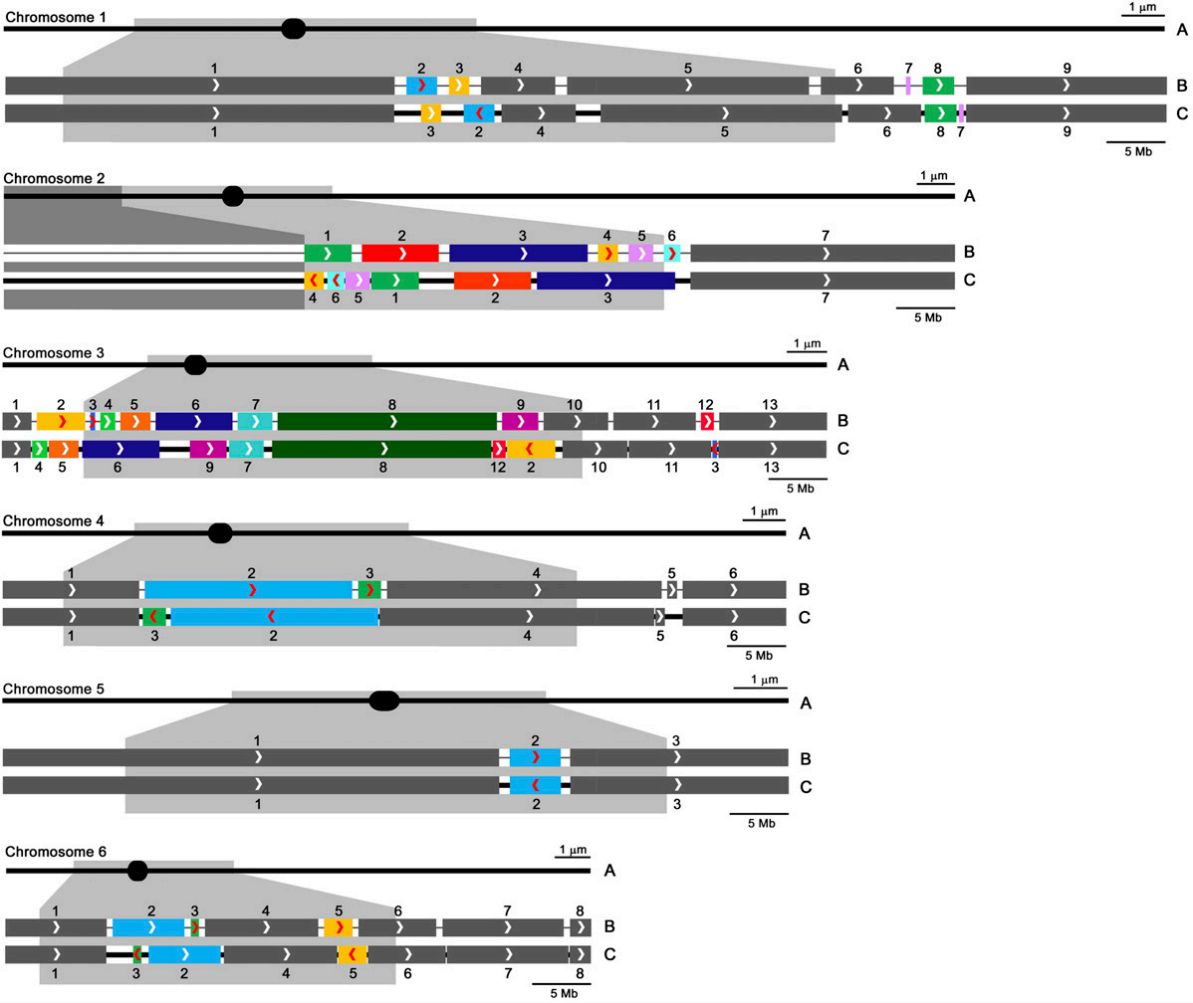
Diagrammatic representations of tomato SCs *1–6* with corresponding linkage map–based pseudomolecules and FISH-based pseudomolecules. (A) SCs are represented as horizontal black lines with solid black ellipses indicating the positions of kinetochores at centromeres. All SCs are oriented with short arms to the left. Pericentric heterochromation is represented as a gray layer to either side of kinetochores, and the approximate location of this heterochromatin is projected onto the DNA pseudomolecules below. The NOR heterochromatin in the distal half of the short arm of chromosome *2* is represented by darker gray. In the pairs of pseudomolecules with scaffolds arranged according to the linkage map (B) and according to FISH (C), thick segments represent sequenced scaffolds and gaps between scaffolds are represented by black lines. Arrowheads in the scaffolds indicate the orientation of each scaffold according to the linkage map from head to tail (*i.e.*, arrowheads point toward the end of the long arm of the chromosome), and numbers above scaffolds indicate their order from the head to the tail of the pseudomolecule based on the linkage map (see Table S4 for scaffold names). Gaps in map-based pseudomolecules are shown of equal length because the linkage map does not contribute to estimates of gap sizes. Scaffolds that show different arrangements between the linkage-based and FISH-based pseudomolecules are indicated by colors other than gray, and scaffolds that show different orientations have red arrowheads. In FISH-based pseudomolecules, changes in scaffold order (position) relative to that predicted from the linkage map are obvious, changes in orientation are shown by red arrowheads that are reversed in direction, and the lengths of gaps are proportional to the amount of DNA estimated to be in the gaps (see Table S7 for gap sizes). The upper bar for each pachytene chromosome (A) represents 1 μm of SC length, and the lower bar for each pair of pseudomolecules (B and C) represents 5 Mb.

**Figure 5:**
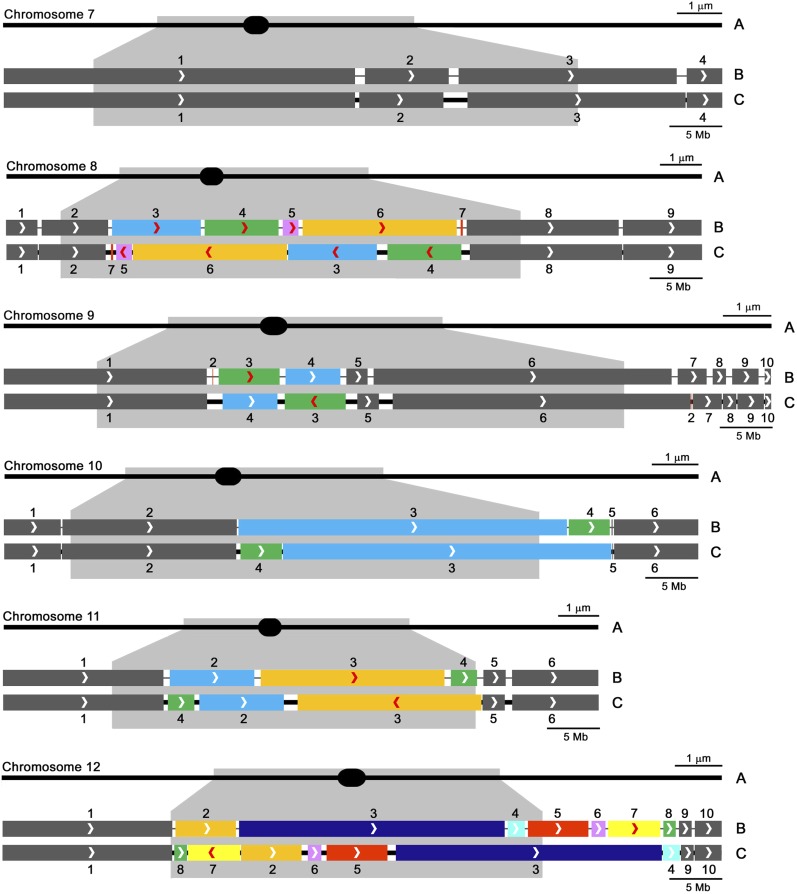
Diagrammatic representations of tomato SCs *7–12* (with corresponding linkage map–based pseudomolecules and FISH-based pseudomolecules below. This figure is a continuation of Figure 4 (see the legend of Figure 4 for details).

Using FISH to localize BACs with sequence at or near the head and tail ends of most scaffolds, we determined the location, order, and head–tail orientation of 87 of the 91 scaffolds. However, only one BAC was localized for each of the four scaffolds that were <400 kb, so order, but not orientation, was determined for these (Figure 2 and Table S4). Figure 3 shows an example of simultaneous FISH localizations of four BACs marking the head and tail ends of two adjacent scaffolds on the short arm of chromosome *3*. Because the chromatin is somewhat dispersed laterally from the SC during the spreading procedure, FISH signals may extend out to either side of the SC and/or may be located directly on the SC (Figure 1 and Figure 3). Scaffold numbering (1, 2, 3, *etc*.) for each pseudomolecule is based on the Kazusa EXPEN 2000 linkage map starting from the end of the short arm (head) of the pseudomolecule (The Tomato Genome Consortium 2012). To facilitate comparisons, these scaffold numbers were also used in pseudomolecules showing FISH-based scaffold orders. Based on FISH, scaffolds 1 and 4 have the same head–tail orientation, but a different order from that based on the linkage map. Details of the differences between linkage map–based *vs.* FISH-based pseudomolecules are given in Table S4 for all 12 chromosomes and are diagrammed in Figure 4 and Figure 5.

In general, scaffolds located in euchromatic regions of the chromosomes are likely to be in the same arrangement in both linkage map–based and FISH-based pseudomolecules, while scaffolds located in pericentric heterochromatin often differ between the two pseudomolecules. In some cases, scaffolds found in one arm by linkage mapping are observed to occur in the other arm by FISH, and scaffolds expected to include the centromere (as indicated by the physical presence of the kinetochore) differ in the two pseudomolecules. While centromere positions in linkage map–based scaffolds are difficult to assign with confidence, the exact locations of kinetochores relative to scaffolds were determined by FISH (Figure 2 and Figure S1). Details of differences between the linkage map-based and FISH-based pseudomolecules for each chromosome are described below.

**Chromosome *1:*** Nine scaffolds. Four of the smaller scaffolds (2, 3, 7, 8) differ in order and scaffold 2 also differs in orientation. Scaffolds 2 and 3 are within pericentric heterochromatin while scaffolds 7 and 8 are in distal euchromatin. Scaffold 7 is very small (400 kb), and its orientation was not determined by FISH. The kinetochore is in the gap between scaffolds 2 and 3.
**Chromosome *2:*** Seven scaffolds. All six scaffolds in or near pericentric heterochromatin (1–6) differ in order and two (4 and 6) also differ in orientation. The kinetochore is in scaffold 2. The distal half of the short arm is composed of heterochromatin (including the NOR), and the DNA in this segment is unsequenced and/or unassembled. Although no estimate of its DNA content was made in the linkage map–based pseudomolecule, the same length is shown in both pseudomolecules to simplify comparison of scaffolds and gaps in the two pseudomolecules. Based on our measurements, we estimate that this 3-μm distal segment includes at least (8.8 Mb/µm × 3 µm =) 26.4 megabases.
**Chromosome *3*:** Thirteen scaffolds. Nine scaffolds (2–9, 12) differ in order, and two (2 and 3) also differ in orientation. The most notable discrepancies include scaffold 2 that was placed in euchromatin of the short arm by linkage mapping but located by FISH in the heterochromatin of the long arm, scaffold 3 (a small scaffold of only ∼400 kb) that was placed in the short arm near/in the heterochromatin by linkage mapping but located in the euchromatin of the long arm by FISH, and scaffold 12 that was located in the middle of the euchromatic portion of the long arm by linkage mapping but located by FISH in or near the relocated scaffold 2 in the heterochromatin of the long arm. The kinetochore is located in the gap between scaffolds 6 and 9. Possibly because we were unable to adequately suppress repeat sequences in the vicinity of scaffolds 2 and 12, the positions of the tail of scaffold 8, both ends of scaffolds 2 and 12, and the head of scaffold 10 are relatively uncertain and, as a consequence, the sizes of gaps 8-12, 12-2, and 2-10 are uncertain as well. Similar difficulties in localizing BACs in this area were encountered using both spreads of SCs and 1:3 acetic ethanol–fixed pachytene chromosomes (Chang 2004) from the two tomato varieties *Cherry* LA4444 and Heinz 1706, so this problem does not appear to be due to a structural difference between the two tomato lines.
**Chromosome *4:*** Six scaffolds. Two scaffolds in heterochromatin (2, 3) differ in both order and orientation by a simple inversion of the sequences. The kinetochore is in the gap between scaffolds 3 and 2.
**Chromosome *5:*** Three scaffolds. The only difference between the two pseudomolecules is the orientation of scaffold 2 that is located in heterochromatin. The kinetochore remains in scaffold 1.
**Chromosome *6:*** Eight scaffolds. Two scaffolds (2, 3), both located in heterochromatin, differ in order, and scaffolds (3 and 5) differ in orientation. The kinetochore is in scaffold 2.
**Chromosome *7:*** Four scaffolds. The arrangement of all four scaffolds is the same in the linkage-based and FISH-based pseudomolecules. The kinetochore is in scaffold 1.
**Chromosome *8:*** Nine scaffolds. Five scaffolds (3–7), all in heterochromatin, differ in order, and scaffolds 3–6 differ in orientation. The small scaffold 7 (200 kb) was localized by only one BAC, so its orientation is not known. The kinetochore is in scaffold 6.
**Chromosome *9:*** Ten scaffolds. Three scaffolds (2–4) differ in order, and scaffold 3 also differs in orientation. Using one fosmid, the tiny scaffold 2 (length not yet determined) was localized by FISH to euchromatin of the long arm compared to its mapped position in heterochromatin of the short arm. The orientation of scaffold 2 was not determined. The kinetochore is mostly in scaffold 4.
**Chromosome *10:*** Six scaffolds. Two scaffolds (3, 4) differ in order with scaffold 4 moving from euchromatin in the long arm to heterochromatin near the kinetochore in the long arm. The kinetochore is in scaffold 4.
**Chromosome *11:*** Six scaffolds. Three scaffolds (2–4) differ in order, and scaffold 3 also differs in orientation. All of these scaffolds are located in heterochromatin. The kinetochore is in scaffold 2.
**Chromosome *12:*** Ten scaffolds. Seven scaffolds (2–8) differ in order, and scaffold 7 also differs in orientation. Notably, scaffolds 5–8 were predicted to be located in euchromatin of the long arm using linkage mapping but were found by FISH to be in the pericentric heterochromatin of the short arm. The kinetochore is in scaffold 5. Unlike any of the other scaffolds, we found an apparent overlap of scaffolds 3 and 5 by FISH on chromosome *12* from LA4444. Using SC spreads from Heinz 1706 for FISH with the same BAC probes, the apparent overlap disappeared, indicating the presence of a small structural inversion difference between the two lines. The inversion includes the kinetochore, the tail of scaffold 5, and the head of scaffold 3 (Figure S3). Even so, a hybrid between LA4444 and Heinz 1706 shows only normal straight synapsis throughout the length of all 12 bivalents, including the presumably nonhomologously synapsed inverted segment around the kinetochore of chromosome *12* (Figure S4). This small inversion was the only structural difference that we identified between the two tomato varieties.

Overall, 46 of the scaffolds, mostly in euchromatin and representing 66% (= 500.2 Mb/760.0 Mb) of the assembled genome, had the same arrangement based on the linkage map compared to FISH. The remaining 45 scaffolds, mostly in heterochromatin and representing 34% (259.8 Mb) of the assembled genome, had different arrangements based on the linkage map compared to FISH. Of these, 28 differed only in order, three differed only in orientation, and 14 differed in both order and orientation. We observed no instance in which a scaffold was located on one chromosome by linkage mapping and on another chromosome by FISH.

### Optical mapping to order and orient scaffolds in pseudomolecules

Optical mapping resulted in 14 superscaffolds bridging two or more adjacent scaffolds (Table S5). Ten chromosomes had at least one superscaffold, and these superscaffolds were observed in both euchromatin and heterochromatin (Figure 4, Figure 5, and Table S5). However, the superscaffolds included only 38 of the 91 total scaffolds, with 53 scaffolds resolving as uninformative singles. The 38 optical scaffolds represent approximately 240.5 Mb (32%) of the total 760 Mb of sequenced tomato genome. The order and orientation of all 38 scaffolds arranged by optical mapping are compatible with FISH results, while the arrangement of only 22 of these 38 scaffolds are compatible with map-based results (Table S5).

### Determining gap sizes between scaffolds

We estimated the linear densities of DNA in kinetochores (centromeres), euchromatin, and heterochromatin to be 3.3, 1.5, and 8.8 Mb/µm, respectively (Table S6 and File S1). Using this information together with the measured distance between two adjacent scaffolds, we estimated individual gap sizes to range from 3.2 Mb down to 0 Mb (very small) (Figure 4, Figure 5, and Table S7). We were able to compare size estimates for 24 gaps using FISH and optical mapping (Table S8). The two values differed from 0 kb to 1.3 Mb, with the paired estimates usually being more similar in euchromatin than in heterochromatin.

With FISH we estimate the total amount of DNA in all 79 gaps in the tomato pseudomolecules to be 43.9 Mb (Table S7) or approximately (43.9 Mb/919 Mb =) 5% of the genome (Table S9). Of this, 34.4 Mb occurs in pericentric heterochromatin, 2.3 Mb occurs in kinetochores, 4.5 Mb occurs in euchromatin/heterochromatin borders, 1.6 Mb occurs in a heterochromatic chromomere, and 1.0 Mb occurs in euchromatin.

### Localizing unassigned (chromosome *0*) BACs

**Figure 6:**
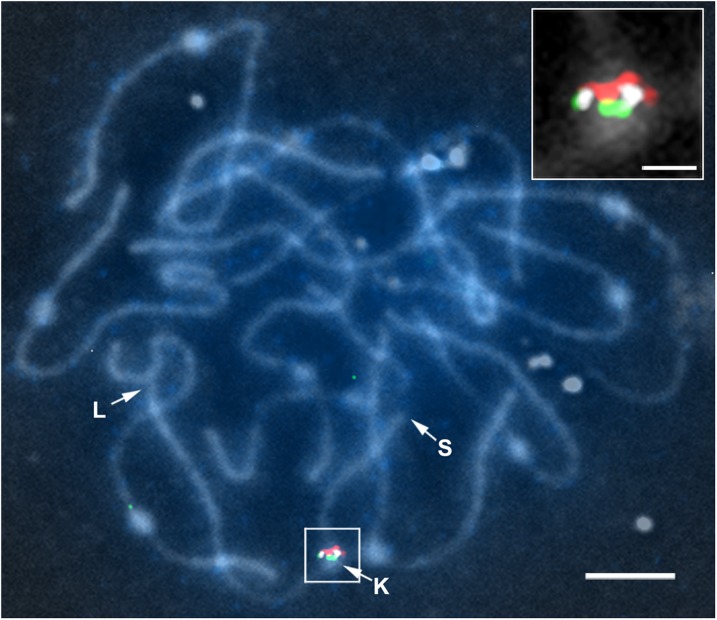
DAPI-stained SC spread showing FISH localizations of an unassigned (chromosome *0*) BAC in a gap between BACs at the ends of adjacent scaffolds on SC *9*. Arrows indicate the ends of the short (S) and long (L) arms of SC*9* as well as the kinetochore (K). BAC SL_*Mbo*I0025N23 (red) is at the tail of scaffold SL2.40sc03771 (scaffold 1 in Figure 5), BAC SL_*Eco*RI0015A15 (green) is at the head of adjacent scaffold SL2.40sc04008 (scaffold 4 in Figure 5), and unassigned BAC SL_*Mbo*I0045G03 (white) is between the red and green signals. The inset shows the hybridization signals and kinetochore of a segment of SC *9* on a magnified reversed phase contrast image of the boxed area. The bar for the large image represents 5 µm, and the bar for the inset represents 1 µm.

Unassigned BACs, also referred to as chromosome *0* BACs, lack sequence alignment and mapped markers so they could not be reliably assembled into any of the pseudomolecules. Of the 93 unassigned BACs that we FISHed, 75 were found at distinct single sites on SCs, and 70 (93%) of these were located in gaps between scaffolds (Figure 2, Figure 6, Figure S1, and Table S10). Five unassigned BACs were located within scaffolds and require further characterization to explain their unassigned designation. The remaining 18 unassigned BACs either did not produce a FISH signal or produced diffuse signals over large areas of heterochromatin. All unassigned BACs are listed in Table S11.

## Discussion

The Kazusa tomato EXPEN 2000 linkage map was used to arrange scaffolds in the recently published tomato genome sequence (Shirasawa *et al.* 2010; The Tomato Genome Consortium 2012). Using FISH as an independent means of ordering and orienting these scaffolds (Figure 1, Figure 2, and Figure 3), we find that 45 of the 91 total scaffolds disagree with the linkage map–based arrangement (Figure 4, Figure 5, and Table S4). Although most of these scaffolds are in heterochromatin, they represent a substantial fraction (34%) of the genome that includes several thousand genes (Wang *et al.* 2006b; Peters *et al.* 2009; Di Filippo *et al.* 2012). So what is the justification for using FISH to order and orient scaffolds when there is a disagreement with linkage mapping?

### FISH is a direct physical means of ordering and orienting scaffolds on chromosomes in both euchromatin and heterochromatin

A pseudomolecule is a sequencing construct that ideally corresponds to the chromosomal DNA molecule. FISH demonstrates this relationship directly by physically hybridizing scaffold DNA to complementary sites on long pachytene chromosomes. In tomato, BAC-FISH was equally successful in both euchromatin and heterochromatin, *i.e.*, most BACS carrying DNA from heterochromatic parts of chromosomes hybridized to single sites when repeated sequences were blocked (Joos *et al.* 1994; Sadder *et al.* 2000; Schubert *et al.* 2001; Szinay *et al.* 2010). This result also indicates that most BAC inserts carried single-copy sequence, which agrees with reports that at least 50% of the DNA in tomato heterochromatin shows single copy reassociation kinetics (Zamir and Tanksley 1988; Peterson *et al.* 1998).

In comparison, the linkage map acts as an intermediary between scaffolds and corresponding chromosomal DNA molecules, so scaffold placement can be no more accurate than the linkage map. Linkage maps are most accurate in regions of the genome that have high rates of recombination, *e.g.*, distal euchromatin, where map-based scaffold arrangements often agree with FISH and optical mapping-based arrangements. However, crossing over is suppressed in heterochromatin, with the result that accuracy of the linkage maps in heterochromatin is correspondingly low (Tanksley *et al.* 1992; Sherman and Stack 1995). This can result in mistakes in arranging scaffolds and, indeed, most discrepancies in scaffold arrangement occur in pericentric heterochromatin (Figure 4 and Figure 5). Similar discrepancies in scaffold arrangements have been reported in heterochromatin of a cucumber chromosome and a barley chromosome (Yang *et al.* 2012; Karafiátová *et al.* 2013). Another problem with arranging scaffolds by linkage maps is that scaffolds must include mapped markers, and this requirement may not be met by some small scaffolds.

### Scaffolds are accurately positioned by FISH on pachytene chromosomes

In tomato, SCs are more than 10-times longer than corresponding C-metaphase chromosomes, and SC spreads have little or no distortion (Sherman and Stack 1995; Stack *et al.* 2009). These two features contribute to precise and reproducible BAC-FISH localizations with SDs of approximately 0.1 µm for the final position of each BAC based on measurements from at least 10 SC spreads. An SC length of 0.1 µm is equivalent to a resolution of approximately (1.5 Mb/µm × 0.1 µm =) 150 kb in euchromatin and (8.8 Mb/µm × 0.1 µm =) 880 kb in heterochromatin. Similar BAC-FISH resolution has been reported for pachytene chromosomes of tomato and other plant species when anthers were fixed with 1:3 acetic ethanol that lengthens pachytene chromosomes approximately two-fold compared to the formaldehyde fixation we used (De Jong *et al.* 1999; Kulikova *et al.* 2001; Cheng *et al.* 2001, 2002; Wang *et al.* 2006b; Szinay *et al.* 2008; Peters *et al.* 2009).

### The Kazusa tomato EXPEN 2000 linkage map is based on a hybrid

Tomato has unusually low genetic diversity because it went through more than one genetic bottleneck during domestication (Rick 1991). To obtain more heterozygosity for genetic mapping, the Kazusa tomato EXPEN 2000 linkage map is based on a hybrid cross between the *Solanum lycopersicum* cultivar LA925 and the wild tomato species *S. pennellii* cultivar LA716 (Fulton *et al.* 2002; Frary *et al.* 2005; Shirasawa *et al.* 2010). For the EXPEN 2000 linkage map to correspond precisely to cultivated tomato chromosomes, the genomes of the two species have to be structurally identical, *i.e.*, no major inversion, translocation, duplication, or deletion differences. While this was generally thought to be the case when the precursor EXPEN 1992 map was published (Khush and Rick 1963; Tanksley *et al.* 1992), we now know that the two species differ in both genome size and organization (Anderson *et al.* 2010; Szinay *et al.* 2012). The genome of *S. pennellii* is 20–30% larger than the tomato genome, and electron microscopic examination of SC spreads from an F1 hybrid revealed a large segment of foldback synapsis, a small inversion loop, and at least five SCs with mismatched kinetochores (Anderson *et al.* 2010). Mismatched kinetochores indicate proximal nonhomologous synapsis and/or neocentromere formation that are consistent with reports of enhanced suppression of proximal crossing over in a tomato × *S. pennellii* F1 hybrid compared to tomato controls (Rick 1969, 1972; Zhang *et al.* 2014). Such structural differences between chromosomes of the two species may cause problems in the linkage map that interfere with its use in ordering and orienting scaffolds. However, the generally good agreement between map-based and FISH-based pseudomolecules in distal euchromatin indicates that most of the major differences between the two genomes occur in pericentric heterochromatin.

### Optical mapping strongly supports the FISH-based scaffold arrangement

Optical mapping is an independent physical method used for arranging the scaffolds of pseudomolecules in several eukaryotic genomes (Zhou *et al.* 2009; Young *et al.* 2011; Dong *et al.* 2013; Chamala *et al.* 2013). Like FISH, optical mapping works in euchromatin and heterochromatin. Though useful for only part of the tomato genome, optical mapping was always consistent with FISH-based arrangements of scaffolds but often differed from linkage-based arrangements (Table S5).

Because the results of BAC-FISH and optical mapping strongly indicate that many scaffolds were arranged incorrectly, the FISH-based arrangement of scaffolds is being used in the new release of the tomato genome build (SL2.5, http://solgenomics.net/organism/Solanum_lycopersicum/genome). In a role reversal, the new scaffold arrangement based on physical methods should be used to rearrange hundreds of markers in the linkage map, particularly in heterochromatin.

### Gap sizes between adjacent scaffolds were estimated by FISH

While linkage mapping provides little help with determining the sizes of gaps in sequencing and/or assembly between scaffolds, FISH permits estimates of the amounts of DNA in gaps based on gap lengths and linear DNA densities of 3.3 Mb/µm for kinetochores, 1.5 Mb/µm for euchromatin, and 8.8 Mb/µm for heterochromatin (Table S6). Approximately similar estimates of linear DNA densities have been made before for euchromatin and heterochromatin in 1:3 acetic ethanol-fixed tomato pachytene chromosomes (Szinay *et al.* 2008). We found individual gap sizes ranging between 0 and 3.2 Mb. Overall, we estimate that 43.9 Mb or approximately (43.9 Mb/919 Mb =) 5% of the genome is in gaps between scaffolds and thus unincorporated into the genome assembly (Table S7).

A caveat to our current estimates of linear DNA density is the assumption that the sequenced scaffold lengths are correct. In euchromatin, where the scaffolds are more likely to be completely sequenced, our estimate of 1.5 Mb/µm matches the value determined using microspectrophotometry of pachytene chromosomes (Peterson *et al.* 1996; Stack *et al.* 2009). However, our estimate of 8.8 Mb/µm for the linear density of DNA in heterochromatin is lower than the estimate of 9.2 Mb/µm based on microspectrophotometry (Peterson *et al.* 1996; Stack *et al.* 2009). This may indicate that 8.8 Mb/µm is an underestimate for heterochromatin where repeated sequences are more likely to interfere with sequencing and/or assembly.

We were unable to make such a genome-wide estimate of gap sizes using optical mapping because 53 of the 91 scaffolds resolved as singletons. However, the gap sizes estimated by FISH were almost always larger than those estimated by optical mapping (Table S8), so if optical mapping is more accurate for estimating gap sizes, then the true amount of DNA in gaps may be less than our estimate based on FISH. Although it is not clear why gap size estimates by FISH tend to be larger, the resolution limits of light microscopy as well as the use of average values to estimate linear DNA density could be involved. Regardless of the approach used to assess the sizes of gaps, a number of gaps are so small (<100 kb) that it is surprising that no BACs (that average ∼100 kb inserts) have been found to bridge the gaps. Possibly some of these gaps are caused by simple sequence repeats (Chang *et al.* 2008) that make assembly difficult, and incorrect arrangement of some scaffolds using the linkage map may also have made it difficult to find BACs that bridge gaps. If so, then the rearrangement of scaffolds based on FISH results should aid in joining at least some adjacent scaffolds.

### FISH locates most unassigned (chromosome *0*) BACs in gaps between scaffolds

BACs with sequence that does not fit in any scaffold would be predicted to be located in gaps between scaffolds and, indeed, FISH indicates this is the case (Figure 2, Figure 6, and Figure S1). These unassigned BACs are entrées for sequencing and/or assembling sequence in the gaps where they are located, while the few unassigned BACs that are located by FISH within scaffolds suggest assembly errors that can be corrected.

### FISH relates pseudomolecules and linkage maps to the structure of pachytene chromosomes

FISH has been used before to relate physical maps and pachytene chromosomes to linkage maps, *e.g.*, in rice (Cheng *et al.* 2001), potato (Iovene *et al.* 2008), papaya (Wai *et al.* 2012), and maize (Wang *et al.* 2006a). In tomato, also, pseudomolecules can be superimposed on the structure of pachytene chromosomes so euchromatin, heterochromatin, and kinetochores can be accurately related to scaffolds and pseudomolecules (Figures 2 and Figure S1). Similarly, molecular markers in the Kazusa EXPEN 2000 linkage map have known positions in pseudomolecules, so the tomato linkage map should be superimposable on pachytene chromosomes as well (Koo *et al.* 2008; The Tomato Genome Consortium 2012). However, this requires reconciliation of the linkage map with the arrangement of scaffolds determined by FISH and optical mapping, especially in pericentric heterochromatin where reduced crossing-over makes the linkage map less reliable.

A previous effort to superimpose the tomato linkage map on tomato pachytene chromosome *1* took advantage of the distribution of recombination nodules that are cytological markers for crossovers on SCs (Chang *et al.* 2007). While FISH showed generally good agreement between observed marker positions on the SCs and the positions predicted from combining the RN and EXPEN 2000 linkage maps, one exceptional segment of SC *1* had three adjacent marker BACs that did not fit well with their expected locations [see Figure 3B in (Chang *et al.* 2007)]. It probably is not a coincidence that these BACs are located in the same region as scaffolds 7 and 8 that appear by FISH to be out of order in the linkage map–based pseudomolecule for chromosome *1* (Figure 4, Table S4, and Table S5).

### FISH shows that recent duplications in the tomato genome are rare

With 627 of 639 BACs localizing to single sites, there appear to be few duplications in the tomato genome based on hybridization at 80% stringency. This is a bit surprising considering that the tomato lineage is thought to have undergone a genome triplication 71 (±19) million years ago (The Tomato Genome Consortium 2012). Apparently, there has been enough gene loss and mutation since the most recent polyploidy event to make tomato effectively diploid, *i.e.*, most genes may have been duplicated, but in the meantime one copy was lost or the two copies diverged sufficiently to no longer hybridize (Rick 1991; The Tomato Genome Consortium 2012).

### Limitations of BAC-FISH on spreads of pachytene chromosomes for assembling pseudomolecules

Like most cytological techniques, BAC-FISH is not high-throughput. For instance, starting with bacterial cultures, it may take a skilled worker 1 month to precisely localize BACs to define the location of a scaffold, although FISH experiments can be staggered and run in parallel. Even so, for organisms in which the genome sequence is assembled into hundreds or thousands of scaffolds, BAC-FISH may not be a cost-effective approach to build pseudomolecules corresponding to chromosomes. Also, locating BACs only at the ends of long scaffolds by FISH does not test the correctness of contig arrangement within scaffolds. Our localization of five unassigned BACs within scaffolds in heterochromatin may indicate problems with assembly and/or sequencing (Karafiátová *et al.* 2013). Furthermore, the accuracy of BAC-FISH is limited both by the resolving power of light microscopy and by differences in levels of chromatin compaction along SCs. In addition, utilizing BAC-FISH for genome assembly requires prior knowledge of chromosome architecture and the ability to prepare clean, well-separated, distortion-free, pachytene chromosome or SC spreads suitable for FISH [see Supplementary Figure 24 in The Tomato Genome Consortium (2012)]. In this regard, tomato was an ideal candidate for arranging scaffolds using BAC-FISH because it has been a cytogenetic model for many years (Rick and Butler 1956; Ramanna and Prakken 1967; Stack *et al.* 2009; Szinay *et al.* 2012), and the proportion of highly repeated sequences in the tomato genome is low (Peterson *et al.* 1998). However, species with large genomes and many repeated sequences, *e.g.*, maize, may require SC spreads, single copy probes, more sensitive FISH techniques, and/or more effective means of blocking repeated sequences to effectively use BAC-FISH for checking genome assembly (Zhong *et al.* 2001; Stack and Anderson 2002; Koumbaris and Bass 2003; Kato *et al.* 2006; Wang *et al.* 2006a). When BAC-FISH is impractical, optical mapping may be a feasible alternative (or a supplement) to linkage map–based genome assemblies (Zhou *et al.* 2007a,b; Chamala *et al.* 2013), with the caveat that optical mapping was capable of ordering fewer than half of the scaffolds in the tomato genome. However, BAC-FISH is a powerful technique for determining overall scaffold arrangement for organisms in which genome assembly is relatively complete and in which adequate chromosome spreads can be prepared.

## Conclusions

The accuracy of the FISH-based arrangement of scaffolds in tomato is strongly supported by an independent physical method, optical mapping, and the internal consistency of FISH results. By this we mean that the FISH-based arrangement of scaffolds covers most of the length of the tomato chromosomes with only small gaps, scaffolds do not overlap, and unanchored BACs localize primarily to gaps between scaffolds. Because of this, the FISH-based arrangement of scaffolds is being used in the new release of the tomato genome build (SL2.5) on the SGN website (http://solgenomics.net/organism/Solanum_lycopersicum/genome). Furthermore, because tomato scaffolds have linkage markers, the new build can be used to improve the tomato linkage map, particularly in regions where crossing over is suppressed. Finally, our results suggest the existence of similar problems in the arrangement of scaffolds in other large genomes that were assembled according to linkage maps (Karafiátová *et al.* 2013; The International Barley Genome Sequencing Consortium 2014) while providing possible approaches for correcting those problems.

## Supplementary Material

Supporting Information
