# Supplementary material for: Fluorescence In Situ Hybridization and Optical Mapping to Correct Scaffold Arrangement in the Tomato Genome
Source: G3 (Bethesda). 2014 May 30;4(8):1395–405. doi: 10.1534/g3.114.011197 (PMC4132171; doi:10.1534/g3.114.011197)
Supplement: Supporting Information [file supp_4_8_1395__index.html]

Fluorescence In Situ Hybridization and Optical Mapping to Correct Scaffold Arrangement in the Tomato Genome — Supporting Information 

# Fluorescence *In Situ* Hybridization and Optical Mapping to Correct Scaffold Arrangement in the Tomato Genome

## Supporting Information for Shearer *et al.*, 2014

**Files in this Data Supplement:**

- Supporting Information - File S1, Figures S1-S4, and Tables S1-S11 (PDF, 1 MB)
- File S1 - Materials and Methods (PDF, 131 KB)
- Figure S1 - Diagrammatic representation of BAC- and repeated sequence-FISH localizations relative to inferred scaffold positions on an idiogram of the twelve tomato SCs. (PDF, 202 KB)
- Figure S2 - Idiogram of tomato SCs with FISH localizations of BACs having two hybridization sites. (PDF, 136 KB)
- Figure S3 - FISH reveals a probable inversion around the centromere of chromosome *12*, which distinguishes tomato var. *Cherry*, accession LA4444 and *S. lycopersicum*, var. Heinz 1706. (PDF, 219 KB)
- Figure S4 - Electron micrographs of phosphotungstic acid (PTA)-stained SCs from a Heinz 1706 X Cherry LA4444 tomato hybrid. (PDF, 811 KB)
- Table S2 - Tomato SC karyotype. (PDF, 119 KB)
- Table S3 - All BACs and repeated sequences located by FISH on tomato SCs. (PDF, 220 KB)
- Table S4 - FISH-based scaffold order, orientation, size, and chromatin type. (PDF, 135 KB)
- Table S5 - Comparison of scaffold order and orientation based on linkage, optical mapping, and FISH. (PDF, 131 KB)
- Table S6 - Estimating the linear density of DNA in euchromatin, heterochromatin, and kinetochores by FISH. (PDF, 121 KB)
- Table S7 - Gap sizes between scaffolds determined using BAC-FISH. (PDF, 127 KB)
- Table S8 - Comparison of gap sizes by optical mapping and FISH. (PDF, 121 KB)
- Table S9 - Estimates of tomato genome size (1C DNA amount). (PDF, 123 KB)
- Table S10 - Location of unassigned (chromosome *0*) BACs by FISH. (PDF, 141 KB)
- Table S1 - Master Scaffold List of all BACs in tomato genome sequence. (.xls, 4 MB)
- Table S11 - List of all of unassigned (chromosome *0*) BACs. (.xls, 729 KB)
