# Supplementary material for: Fluorescence In Situ Hybridization and Optical Mapping to Correct Scaffold Arrangement in the Tomato Genome
Source: G3 (Bethesda). 2014 May 30;4(8):1395–405. doi: 10.1534/g3.114.011197 (PMC4132171; doi:10.1534/g3.114.011197)
Supplement: Supporting Information [file supp_g3.114.011197_011197SI.pdf]

G3: Genes | Genomes | Genetics

Fluorescence *in situ* hybridization and optical mapping to correct scaffold arrangement in the tomato genome

Lindsay A. Shearer,* Lorinda K. Anderson,* Hans de Jong,§ Sandra Smit,† José Luis Goicoechea,‡ Bruce A. Roe,** Axin Hua,** James J. Giovannoni, §§ and Stephen M. Stack*

* Department of Biology, Colorado State University, Fort Collins, CO 80525, USA
§ Laboratory of Genetics, Wageningen University and Research Centre (WUR), Droevendaalsesteeg 1, 6708 PB Wageningen, The Netherlands
† Laboratory of Bioinformatics, WUR, Droevendaalsesteeg1, 6708 PB Wageningen, The Netherlands
‡ Arizona Genomics Institute, University of Arizona, Tucson, AZ 85721
** Department of Chemistry and Biochemistry, Stephenson Research and Technology Center, University of Oklahoma, Norman, OK, USA 73019

§§ Department of Plant Biology, Cornell University, Ithaca, NY 14853, US
Corresponding author:
Stephen Stack
Department of Biology
Colorado State University
1878 Campus Delivery
Fort Collins, CO 80523-1878
Phone: 970-491-6802
Stephen.Stack@colostate.edu

DOI: 10.1534/g3.114.011197

File S1

Materials and Methods

Isolating BACs. The following modifications were made in the standard protocol for plasmid isolation using an AquaPlasmid kit (MultiTarget Pharmaceuticals, Salt Lake City, Utah). Bacterial culture volume was increased from 2 ml to 4 ml. After removing supernatant from the bacterial pellet, bacteria were suspended in 100 µL of deionized water containing 400 µg/ml ribonuclease A instead of 80 µl of deionized water. After addition of AquaLysis, instead of mixing by vortexing, the tubes were rocked gently for 5 minutes. 50 µL of 3M sodium acetate was added to each tube and tubes were rocked gently for 5 minutes to mix. Then AquaPlasmid reagents were added and mixed gently by rocking for 5 minutes. Centrifugation was increased from 5 minutes to 20 minutes to pellet debris after the addition of AquaLysis.

Determining the amount of DNA per micrometer of SC (= linear DNA density) in kinetochores, euchromatin, and heterochromatin. The lengths of scaffolds that are wholly contained in a kinetochore, or in distal euchromatin, or in pericentric heterochromatin were measured as a percent of arm length between the fluorescent signal of a BAC at the head of the scaffold and the fluorescent signal of a BAC at the tail of the scaffold. These percent lengths were converted to micrometers based on the average pachytene SC set (see *Measuring positions of BACs on SCs* in Materials and Methods). Before linear density of DNA can be calculated, a correction must be made to take into account the fact that amount of DNA (scaffold size) between FISH foci is actually an overestimate because 1) a FISH focus is considered to be in the center of a BAC and 2) sometimes subterminal BACs rather than terminal BACs in scaffolds were used for FISH in cases where terminal BACs did not work for FISH. Therefore, there is less DNA between the FISH signals than in the scaffold because some scaffold DNA extends beyond the FISH signals into the adjacent gaps. As a correction, the scaffold DNA extending beyond the FISH signals was subtracted from the scaffold size to yield the amount of DNA between the FISH foci. For example, based on BAC-FISH, scaffold SL2.40sc04626 in the euchromatin of the long arm of SC *7* is measured to be 2.0 µm long. This scaffold is known to be is 3.39 Mb long (http://solgenomics.net). On the left side of the scaffold, the localized BAC SL_EcoRI0032D11 is 150 kb long. Assuming the fluorescent signal is in the middle of the BAC, (150 kb ÷ 2 =) 75 kb that extends beyond the FISH signal was subtracted from the scaffold size. Because this BAC starts 11 kb into the scaffold, another 11 kb was subtracted from the scaffold size. On the right side of the scaffold the localized BAC SL_MboI0017L19 is 74 Mb long, so an additional (74 Mb ÷ 2 =) 37 kb was subtracted from the scaffold size. Also, this BAC is not terminal, but starts 99 kb from the right end of the scaffold, so an additional 99 kb was subtracted from the scaffold size. Thus, the corrected amount of DNA between these two FISH signals is (3390 kb – 11 kb – 75 kb – 37 kb – 99 kb =) 3168 kb or ~3.2 Mb compared to the complete scaffold size of 3.39 Mb.

Adjusting measured gap sizes. Gap lengths were initially determined by measuring the distance between the fluorescent focus of a BAC at or near the end of one scaffold and the fluorescent focus of another BAC at or near the end of the adjacent scaffold. This measurement, expressed as a percent of the arm length, was repeated on ten or more SC spreads to obtain an average distance expressed as a percent of the arm length between the two BACs. This percent distance was converted to micrometers by multiplying the percent (decimal fraction) times the average length of this SC arm in micrometers that was determined by Sherman and Stack (1992). This distance in micrometers was converted to a quantity of DNA based on the linear (DNA) density of the chromatin type where the gap is located (Table S6). Gap sizes calculated this way are overestimates because 1) FISH foci are assumed to be in the center of a localized BAC and 2) subterminal BACs rather than terminal BACs in scaffolds often were used for FISH. Therefore, the measured gap sizes should be reduced by the amount of DNA estimated to extend from the FISH signals into the gap. For example, gap 9-10 on the long arm of SC 12, *i.e*., the gap between scaffolds SL2.40sc05611and SL2.40sc05380, was measured to be 0.12 µm long in euchromatin and the amount of DNA in the gap is estimated to be (1.5 Mb/µm X 0.12 µm =) 0.18 Mb or 180 kb. On the left side of the gap, the localized BAC SL_EcoRI0024H05 is 106 kb long, and this BAC is the terminal (the last) BAC in the scaffold. Assuming that the fluorescent focus is in the middle of the BAC (106 kb ÷ 2 =) 53 kb should be subtracted from the gap size. On the right side of the scaffold the localized BAC Le_HBa0029L21 is 120 kb long, so an additional (120kb ÷ 2=) 60 kb should be subtracted from the scaffold size. Also the BAC localized to the right is not the terminal BAC, but 53 kb from the end of the right scaffold, so this amount of DNA also should be subtracted from the gap size. Thus, the corrected gap size is (180 kb – 53 kb – 60 kb – 53 kb =) 14 kb.

REFERENCE

Sherman, J. D. and S. M. Stack, 1992 Two-dimensional spreads of synaptonemal complexes from solanaceous plants. V. Tomato (*Lycopersicon esculentum*) karyotype and idiogram. Genome **35**: 354-359.

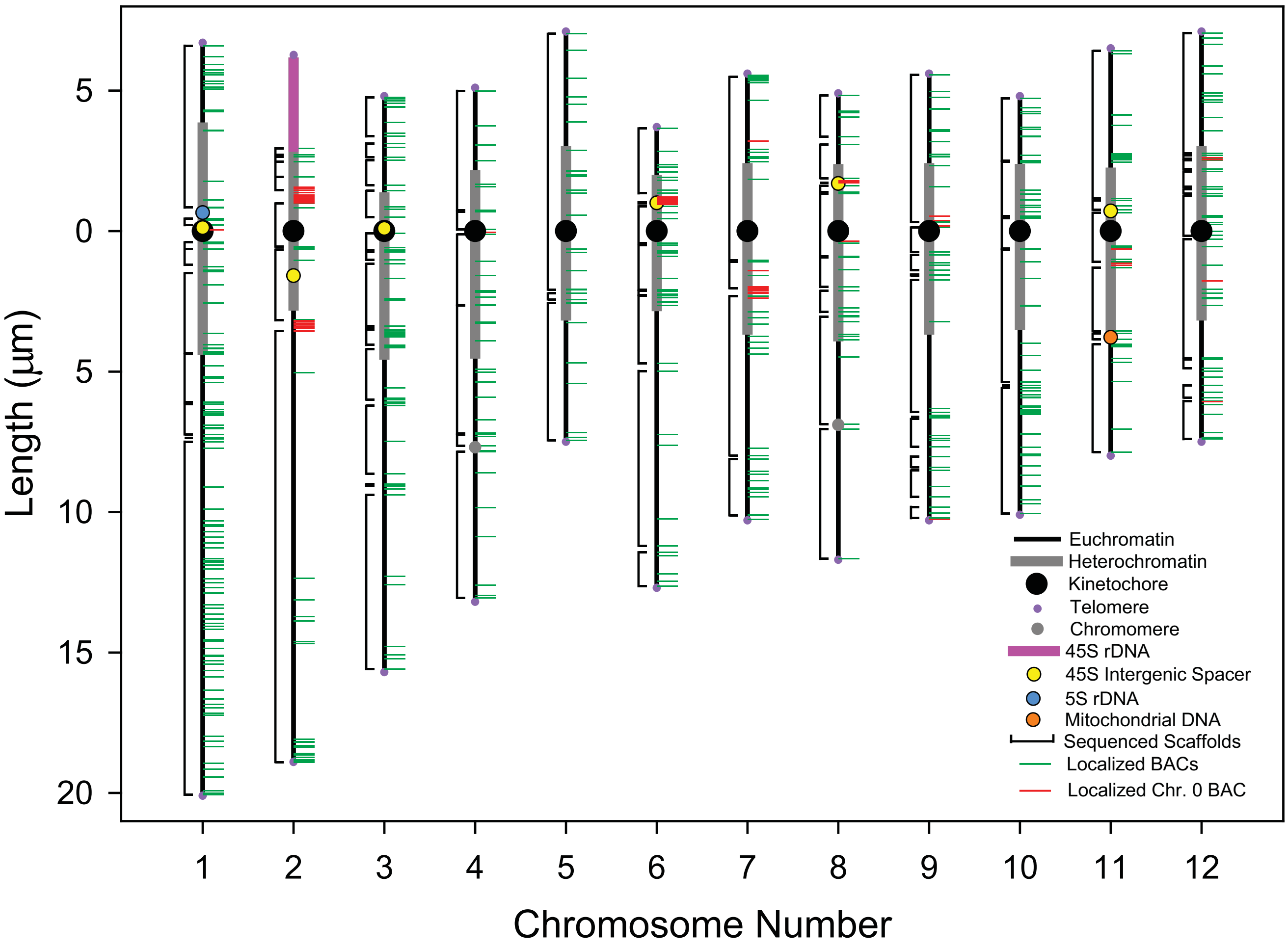

Figure S1 Diagrammatic representation of BAC- and repeated sequence-FISH localizations relative to inferred scaffold positions on an idiogram of the twelve tomato SCs. The twelve vertically oriented tomato SCs are numbered along the bottom. For each SC, black lines represent SC in distal euchromatin, thick grey lines represent SC in pericentric heterochromatin, and kinetochores (centromeres) are shown as black disks aligned at 0 µm on the left scale. Grey dots in the euchromatin of the long arms of SCs *4* and *8* represent chromomeres. The short arm of chromosome *2* is entirely heterochromatic with the nucleolus organizer region (NOR) in the thick, pink, terminal segment marking the location of 45S rDNA. Other repeated sequences localized by FISH include sites of 45S rDNA intergenic spacer sequence (yellow spots on SCs *1*, *2*, *3*, *6*, *8* and *11)*, 5S rDNA (blue spot in the short arm of SC *1*), a concentration of mitochondrial DNA (orange spot on SC *11*), and canonical $(TTTAGGG)_n$ telomere repeats (purple knobs at the ends of all chromosomes). Short horizontal red and green lines extending to the right of each SC show BACs located by FISH. Red lines indicate the locations of 75 chromosome *0* BACs, while green lines indicate the locations of all other BACs. A total of 627 BACs have been localized, but there are 639 horizontal lines because twelve of the BACs hybridize to two locations (Figure S1). See http://solgenomics.net/cview/map.pl?map_version_id=25 for identification of each BAC on the idiogram and the photographic data for every FISH localization. Brackets to the left of each SC mark chromosomal locations of scaffolds, while spaces between brackets represent gaps between scaffolds. Some scaffolds are so small that they appear only as lines in gaps, and some gaps are so small that no space is visible between adjacent brackets. Note that many chromosome *0* BACs (red horizontal lines) occur in gaps and that most of the locations of repeated sequences also correspond to gaps between sequenced scaffolds.

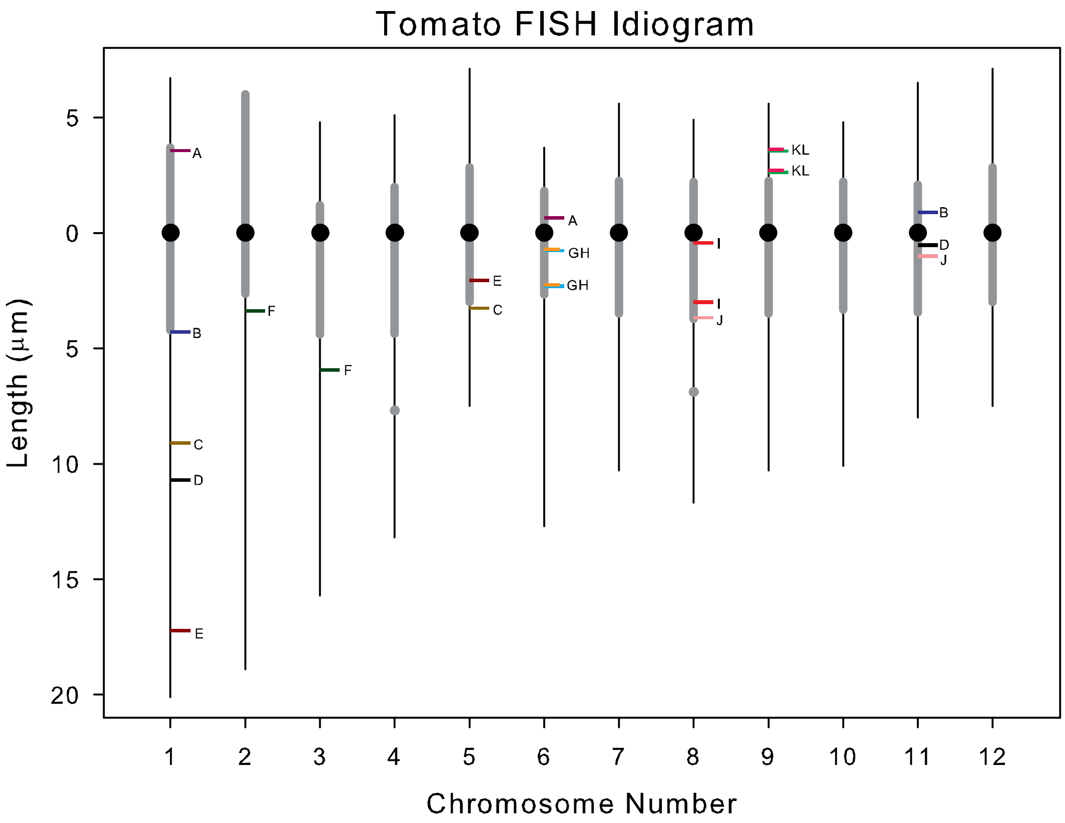

Figure S2 Idiogram of tomato SCs with FISH localizations of BACs having two hybridization sites. Each BAC is represented by two horizontal bars of the same color. Five BACs hybridize to two locations on the same SC. Two of these BACs are close to each other on SC *6* (G and H), two are close to each other on SC *9* (K and L), and one is on SC *8* (I). On SCs *6* and *9*, the two BACs are so close together that they must carry DNA sequence from the same small duplicated chromosome segment. The other seven BACs each hybridize at one location on two different SCs, A on *1* and *6*, B on *1* and *11*, C on *1* and *5*, D on *1* and *11*, E on *1* and *5*, F on *2* and *3*, J on *8* and *11*. These apparent duplications also could be due to chimeric BACs (one BAC with two random segments of tomato DNA), and/or cultures with two BACs, *i.e.*, contaminated cultures. Key to duplications: A is SL_MboI0081L19, B is LE_HBa0023H04, C is LE_HBa0006D05, D is LE_HBa0305L18, E is LE_HBa0033M02, F is LE_HBa0101C24, G is LE_HBa0031J01, H is SL_MboI0034N14, I is SL_MboI0012P13, J is SL_EcoRI0027A06, K is LE_HBa0300E15 and L is LE_HBa0026P14.

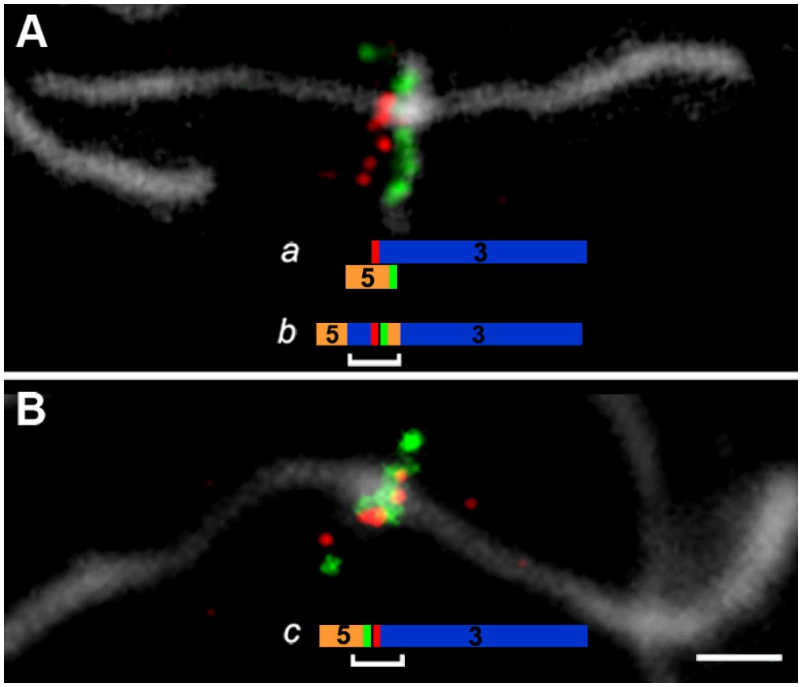

Figure S3 FISH reveals a probable inversion around the centromere of chromosome *12*, which distinguishes tomato var. *Cherry*, accession LA4444 and *S. lycopersicum*, var. Heinz 1706. Digitally reversed phase images of SC *12* from (**A**) LA4444 and (**B**) Heinz 1706 with overlaid FISH signals for probes LE_HBa0005D14 (red) that is part of scaffold SL2.40sc04057 (= scaffold 3) and SL_EcoRI0012H19 (green) that is part of scaffold SL2.40sc04757 (= scaffold 5). Both SCs are oriented with the short arms to the left. Both probes hybridize near the centromere in both lines, but the order of the two probes relative to the short arm differs. Although not shown here, probes that mark the opposite ends of scaffolds 3 and 5 are located in the same relative positions in the long and short arms in both lines. Interpretive drawings (a-*c*) of scaffolds 3 (blue) and 5 (orange) with the red bar indicating the approximate position of probe LE_HBa0005D14 on scaffold 3 and the green bar indicating the approximate position of probe SL_EcoRI0012H19 on scaffold 5. (***a***) The positions of the probes on these two scaffolds indicate that the scaffolds overlap in LA4444. However, sequencing of Heinz 1706 did not reveal any such overlap. (***b***) Diagram of a probable inversion (shown by the bracket underneath) of a segment of chromosome *12* from LA4444 relative to Heinz 1706, which explains the observed probe locations. (***c***) The positions of the probes at the ends of scaffolds 5 and 3 on chromosome *12* of Heinz 1706 are consistent with two adjacent scaffolds. The bracket underneath indicates the location of the chromosome segment inverted between LA4444 and Heinz 1706. Bar equals 2 µm for **A** and **B**. The drawings (***a-c***) are not to scale except the lengths of the two scaffolds are correct relative to each other.

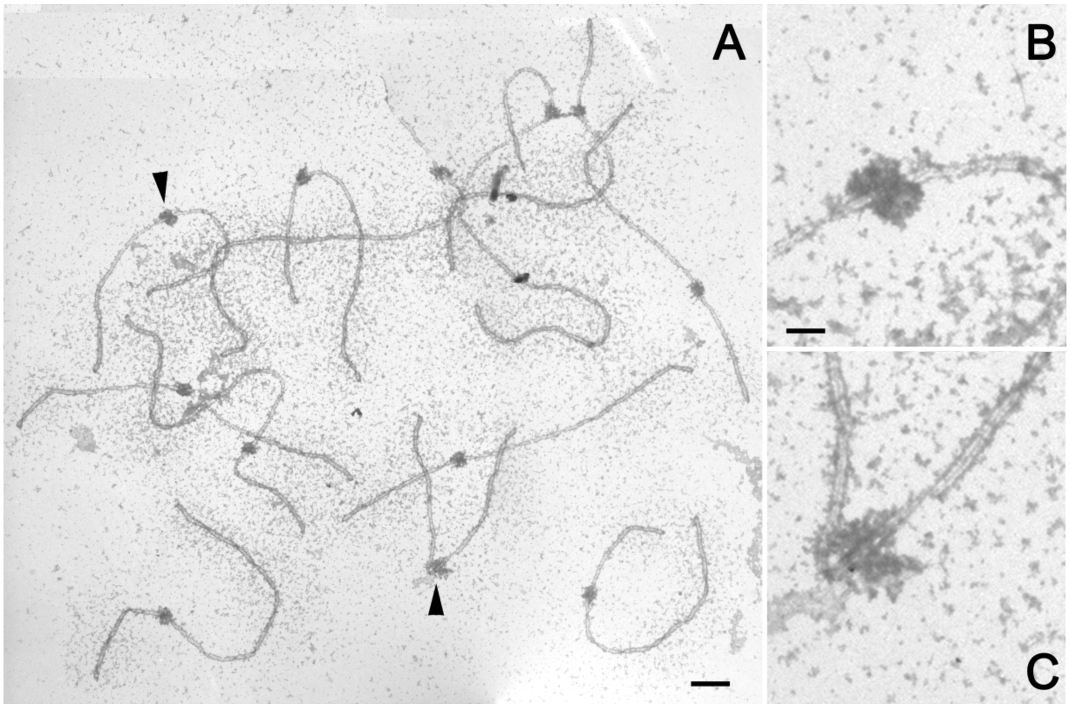

Figure S4 Electron micrographs of phosphotungstic acid (PTA)-stained SCs from a Heinz 1706 X Cherry LA4444 tomato hybrid. **A**. Complete SC spread showing no synaptic irregularities that would indicate structural heterozygosities between the two lines. Chromosomes *5* and *12* (indicated by arrowheads at kinetochores) are structurally indistinguishable. **B**. and **C.** Higher magnification views of pericentric regions of SCs *5* and *12* from (**A**). Both chromosomes show straight synapsis through the kinetochore, even though the hybrid is probably heterozygous for an inversion involving the kinetochore of chromosome *12*. SC spreading and PTA staining was performed as described by Stack and Anderson (2009). Bar in A = 2 µm, bar in B (and C) = 0.5 µm

Table S1 Master Scaffold List of all BACs in tomato genome sequence.

Available for download as an Excel file at http://www.g3journal.org/lookup/suppl/doi:10.1534/g3.114.011197/-/DC1

Table S2 Tomato SC karyotype[1]

Chromosome number	% of set	Chromosome length (µm)	Arm ratio	Short arm length (µm)	Long arm length (µm)
1	13.1	26.8	3.00	6.7	20.1
2	8.5	24.9	3.15	6.0	18.9
3	9.8	20.5	3.27	4.8	15.7
4	8.7	18.3	2.59	5.1	13.2
5	7.2	14.6	1.06	7.1	7.5
6	7.9	16.4	3.43	3.7	12.7
7	7.8	15.9	1.80	5.6	10.3
8	7.9	16.6	2.39	4.9	11.7
9	7.8	15.9	1.83	5.6	10.3
10	7.2	14.9	2.10	4.8	10.1
11	6.9	14.5	1.23	6.5	8.0
12	7.2	14.6	1.06	7.1	7.5
**Total**	**100.0**	**213.9**		**67.9**	**146.0**

[1]Modified from Sherman and Stack (1992) and Peterson et al. (1995)

Table S3 All BACs and repeated sequences located by FISH on tomato SCs. Unless otherwise noted, all BACs are from the HindIII BAC library.

Chromosome arm	BAC	Position %	Position um (SC)	Scaffold	Legend Key
	Telomere	100.0%	0.0		**S = short arm; L = long arm**
**1S**	**SL_s0090M22**	**98.5%**	**0.1**	**S1**	Telomere
					Eu/Het border using RNs to adjust estimate based on SC appearance
1S	165H20	92.6%	0.5		
1S	088A08	88.5%	0.8		Centromere
					Less than 1 micron
1S	130I12	85.7%	1.0		from a telomere
1S	069E17	84.1%	1.1		or eu/het border
					Less than 1 micron
1S	240P19	83.0%	1.1		from a centromere
1S	262O22	79.7%	1.4		**End of a scaffold**
1S	305J13	78.3%	1.5		BAC with 2 positions
1S	291L11	76.4%	1.6		Repeated sequences
					IGS = inter-genic
					spacer repeat from rDNA
1S	263P17	75.6%	1.6		
1S	032H01	64.3%	2.4		
1S	003D15	63.5%	2.4		
1S	Eu/Het border	55.0%	3.0		
1S	SL_MboI0012I20	53.5%	3.1		

1S	SL_MboI0081L19	53.2%	3.1	
1S	177F14	26.4%	4.9	
1S	107C12	16.5%	5.6	
**1S**	**SL_MboI0034D03**	**12.5%**	**5.9**	**S1**
1S	027P13	12.4%	5.9	
1S	5S rDNA	9.8%	6.0	
**1S**	**SL_s0053P14**	**6.6%**	**6.3**	**S3**
1S	081I16	5.8%	6.3	
**1S**	**SL_s0083L21**	**3.2%**	**6.5**	**S3**
1S	IGS-derived satellite	1.8%	6.6	
1S	SL_MboI0014K08	0.6%	**6.7**	
	Centromere	0.0%	6.7	
**1L**	**SL_EcoRI0016I11**	**2.0%**	**7.1**	**S2**
1L	054N01	2.2%	7.2	
**1L**	**SL_s0006A13**	**3.2%**	**7.3**	**S2**
**1L**	**033N15**	**3.2%**	**7.3**	**S4**
1L	256E08	6.3%	8.0	
**1L**	**SL_s0042B18**	**6.9%**	**8.1**	**S4**
**1L**	**SL_MboI0042O02**	**7.2%**	**8.1**	**S5**
1L	061J17	9.5%	8.6	
1L	252G05	12.8%	9.3	
1L	017P20	18.1%	10.3	
1L	174H02	20.1%	10.7	
1L	126F09	20.7%	10.9	

1L	048O19	20.8%	10.9	
1L	Eu/Het border	21.0%	10.9	
1L	023H04	21.3%	11.0	
1L	163B20	21.3%	11.0	
**1L**	**037N04**	**21.6%**	**11.0**	**S5**
**1L**	**SL_s0040G18**	**21.8%**	**11.1**	**S6**
1L	095K03	23.9%	11.5	
1L	033C15	25.7%	11.9	
1L	305F14	26.0%	11.9	
1L	087D19	26.8%	12.1	
**1L**	**SL_EcoRI0021C24**	**30.3%**	**12.8**	**S6**
**1L**	**SL_s0022L14**	**30.6%**	**12.9**	**S8**
1L	004L01	30.6%	12.9	
1L	210F09	31.6%	13.1	
1L	SL_MboI0017A22	32.0%	13.1	
1L	SL_MboI0008G17	32.0%	13.1	
1L	SL_MboI0034B03	32.4%	13.2	
1L	SL_EcoRI0021A20	32.6%	13.3	
1L	SL_EcoRI0022O19	34.4%	13.6	
1L	055E23	34.8%	13.7	
1L	001J06	35.0%	13.7	
**1L**	**SL_s0121I01**	**36.0%**	**13.9**	**S8**
**1L**	**SL_s0071P10**	**36.6%**	**14.1**	**S7**
1L	067M04	36.8%	14.1	

1L	039M19	36.8%	14.1	
**1L**	**SL_s0024J19**	**37.3%**	**14.2**	**S9**
1L	029G15	37.3%	14.2	
1L	155M04	38.4%	14.4	
1L	006D05	45.3%	15.8	
1L	329A12	49.2%	16.6	
1L	289N16	51.3%	17.0	
1L	035M19	52.0%	17.2	
1L	182E16	52.3%	17.2	
1L	053C22	52.3%	17.2	
1L	305L18	53.2%	17.4	
1L	208M24	54.2%	17.6	
1L	034P21	55.2%	17.8	
1L	049F03	56.1%	18.0	
1L	330O05	58.0%	18.4	
1L	108J06	58.4%	18.4	
1L	140O23	58.6%	18.5	
1L	234D05	59.1%	18.6	
1L	080J18	59.8%	18.7	
1L	309D12	61.5%	19.1	
1L	125A09	62.3%	19.2	
1L	203E06	63.1%	19.4	
1L	123A07	64.0%	19.6	
1L	169H23	64.0%	19.6	

1L	058O13	64.2%	19.6
1L	057C07	66.2%	20.0
1L	159C14	66.8%	20.1
1L	165M11	66.8%	20.1
1L	051C14	67.8%	20.3
1L	010F03	68.7%	20.5
1L	005L21	69.5%	20.7
1L	001H15	70.0%	20.8
1L	302G11	70.5%	20.9
1L	057A01	72.3%	21.2
1L	SL_EcoRI0127B04	72.6%	21.3
1L	111D21	73.9%	21.6
1L	024O06	75.1%	21.8
1L	043D23	75.3%	21.8
1L	164M23	76.1%	22.0
1L	243A15	76.7%	22.1
1L	026N10	77.8%	22.3
1L	057J16	78.9%	22.6
1L	049P09	81.3%	23.0
1L	037D10	82.9%	23.4
1L	245N21	83.8%	23.5
1L	002F21	83.8%	23.5
1L	100N21	84.4%	23.7
1L	079N04	85.4%	23.9

1L	092H13	85.4%	23.9	
1L	033M02	85.7%	23.9	
1L	239E07	89.5%	24.7	
1L	064D24	90.3%	24.9	
1L	008L19	91.3%	25.0	
1L	131F15	94.2%	25.6	
1L	128J14	95.2%	25.8	
1L	043P11	96.7%	26.1	
1L	231M15	99.1%	26.6	
1L	088L02	99.5%	26.7	
**1L**	**SL_MboI0028C09**	**99.8%**	**26.8**	**S9**
1L	Telomere	100.0%	26.8	
2S	Telo./Break Point*	100.0%	0.0	
2S	45S rDNA	16.0%	3.0	
**2S**	**SL_EcoRI0007F19**	**15.6%**	**3.1**	**S4**
**2S**	**SL_EcoRI0008B15**	**14.4%**	**3.3**	**S4**
**2S**	**SL_s0053E24**	**14.0%**	**3.4**	**S6**
**2S**	**SL_s0044P10**	**13.1%**	**3.5**	**S6**
**2S**	**LE_HBa0011G24**	**13.1%**	**3.5**	**S5**
**2S**	**SL_s0056I16**	**10.2%**	**4.1**	**S5**
**2S**	**SL_MboI0036C03**	**10.2%**	**4.1**	**S1**
2S	SL_MboI0056H03	8.3%	4.4	
2S	SL_MboI0063O23	8.1%	4.5	

**2S**	**SL_s0079J24**	**7.7%**	**4.5**	**S1**
2S	SL_MboI0025G13	7.7%	4.5	
2S	SL_MboI0016G20	7.7%	4.5	
2S	078N01	7.3%	4.6	
2S	SL_EcoRI0040P16	6.8%	4.7	
2S	009K14	6.7%	4.7	
2S	111L05	6.7%	4.7	
2S	SL_EcoRI0024C17	6.6%	4.8	
2S	SL_MboI0034C01	6.2%	4.8	
2S	SL_MboI0050I21	6.0%	4.9	
2S	SL_MboI0034J07	5.9%	4.9	
2S	SL_EcoRI0031O09	5.9%	4.9	
2S	SL_MboI0004J02	5.9%	4.9	
2S	058D23	5.7%	4.9	
2S	043D06	5.6%	4.9	
2S	060L16	5.3%	5.0	
2S	012G12	5.3%	5.0	
**2S**	**SL_s0053C07**	**5.2%**	**5.0**	**S2**
2S	090J13	4.4%	5.2	
	Centromere	0.0%	6.0	
**2L**	**SL_s0050E09**	**2.9%**	**6.5**	**S2**
2L	006P20	3.2%	6.6	
**2L**	**SL_MboI0021A03**	**3.5%**	**6.7**	**S3**
2L	060G11	5.5%	7.0	

2L	IGS-derived satellite	8.4%	7.6	
2L	Eu/Het border	14.0%	8.6	
2L	176A14	16.7%	9.2	
**2L**	**SL_EcoRI0034J17**	**16.8%**	**9.2**	**S3**
2L	SL_MboI0010K24	16.9%	9.2	
2L	SL_EcoRI0032D02	17.0%	9.2	
2L	SL_MboI0050I17	17.3%	9.3	
2L	SL_EcoRI0002O15	17.3%	9.3	
2L	060D16	17.4%	9.3	
2L	SL_MboI0029F22	17.5%	9.3	
2L	101C24	17.9%	9.4	
2L	026M09	17.9%	9.4	
2L	059K12	17.9%	9.4	
2L	SL_EcoRI0006L04	18.1%	9.4	
2L	SL_EcoRI0042F18	18.1%	9.4	
2L	027N03	18.2%	9.4	
2L	SL_EcoRI0007J10	18.3%	9.5	
2L	SL_EcoRI0039I06	18.8%	9.6	
**2L**	**SL_s0101F18**	**18.8%**	**9.6**	**S7**
2L	067G24	18.9%	9.6	
2L	303I24	26.7%	11.0	
2L	286I11	65.4%	18.4	
2L	108A18	69.4%	19.1	
2L	106H06	72.6%	19.7	

2L	118M12	73.4%	19.9	
2L	001M12	77.3%	20.6	
2L	189G15	77.7%	20.7	
2L	SL_MboI0055O24	95.7%	24.1	
2L	064B17	96.2%	24.2	
2L	SL_EcoRI0034H10	96.3%	24.2	
2L	072B02	96.9%	24.3	
2L	SL_EcoRI0042D07	97.2%	24.4	
2L	SL_EcoRI0061K08	98.4%	24.6	
2L	257H21	98.7%	24.7	
2L	098J01	99.1%	24.7	
2L	032J10	99.6%	24.8	
2L	177F12	99.8%	24.9	
**2L**	**SL_MboI0017J13**	**100.0%**	**24.9**	**S7**
2L	Telomere	100.0%	24.9	
3S	Telomere	100.0%	0.0	
**3S**	**SL_s0009C01**	**99.3%**	**0.0**	**S1**
3S	020P05	98.0%	0.1	
3S	SL_MboI0103M17	96.6%	0.2	
3S	203C09	94.8%	0.3	
3S	SL_FOS0082H20	92.4%	0.4	
3S	SL_FOS0097P22	92.1%	0.4	
3S	137K15	80.6%	0.9	
3S	257N18	72.6%	1.3	

**3S**	**SL_s0086D22**	**70.2%**	**1.4**	**S1**
**3S**	**SL_s0018K15**	**65.1%**	**1.7**	**S4**
3S	030A11	65.0%	1.7	
3S	031M05	62.1%	1.8	
**3S**	**SL_s0002G24**	**54.9%**	**2.2**	**S4**
**3S**	**SL_s0050E05**	**52.5%**	**2.3**	**S5**
**3S**	**037B06**	**34.2%**	**3.2**	**S5**
**3S**	**020G20**	**29.8%**	**3.4**	**S6**
3S	Eu/Het border	25.0%	3.6	
3S	130G19	17.9%	3.9	
3S	162G22	16.2%	4.0	
**3S**	**039C15**	**10.2%**	4.3	**S6**
3S	IGS-derived satellite	1.9%	4.7	
	Centromere	0.0%	4.8	
**3L**	**SL_MboI0079O10**	**0.5%**	4.9	**S9**
3L	007J09	3.7%	5.4	
**3L**	**SL_MboI0002J15**	**4.3%**	5.5	**S9**
**3L**	**244B01**	**4.7%**	5.5	**S7**
**3L**	**SL_EcoRI0121G21**	**6.5%**	5.8	**S7**
**3L**	**027L13**	**7.4%**	6.0	**S8**
3L	197O22	10.7%	6.5	
3L	014A17	15.3%	7.2	
3L	077H15	15.6%	7.2	
**3L**	**SL_s0071G06**	**21.5%**	**8.2**	**S8**

**3L**	**SL_s0042B23**	**22.3%**	**8.3**	**S2**
3L	SL_MboI0022A20	22.4%	8.3	
3L	SL_MboI0018H18	22.6%	8.3	
3L	157B13	23.1%	8.4	
3L	011P01	23.2%	8.4	
3L	SL_MboI0023D14	23.2%	8.4	
3L	084A03	23.4%	8.5	
**3L**	**SL_EcoRI0018K21**	**23.4%**	**8.5**	**S12**
**3L**	**028L03**	**23.7%**	**8.5**	**S12**
3L	SL_MboI0011B02	24.0%	8.6	
3L	011C02	25.9%	8.9	
**3L**	**037N01**	**26.1%**	**8.9**	**S10**
3L	SL_MboI0015M02	26.2%	8.9	
**3L**	**030A19**	**26.3%**	**8.9**	**S2**
3L	299H10	26.5%	9.0	
3L	Eu/Het border	28.0%	9.2	
3L	031P17	35.5%	10.4	
3L	101C24	37.9%	10.7	
**3L**	**001H05**	**38.2%**	**10.8**	**S10**
3L	028E17	38.8%	10.9	
3L	254B14	39.1%	10.9	
**3L**	**SL_MboI0003H09**	**39.6%**	**11.0**	**S11**
3L	SL_s0003D15	47.7%	12.3	
**3L**	**SL_EcoRI0031G05**	**55.0%**	**13.4**	**S11**

**3L**	**SL_MboI0015G06**	**57.4%**	**13.8**	**S3**
**3L**	**SL_s0048H23**	**57.7%**	**13.9**	**S3**
3L	SL_EcoRI0034O23	57.9%	13.9	
3L	117I12	58.4%	14.0	
**3L**	**SL_EcoRI0006F17**	**59.8%**	**14.2**	**S13**
3L	157P11	78.3%	17.1	
3L	276M05	80.2%	17.4	
3L	241F16	94.2%	19.6	
3L	142A05	96.0%	19.9	
3L	159C06	97.0%	20.0	
**3L**	**SL_EcoRI0034G21**	**99.3%**	**20.4**	**S13**
3L	Telomere	100.0%	20.5	
4S	Telomere	100.0%	0.0	
**4S**	**SL_MboI0035I14**	**97.9%**	**0.1**	**S1**
4S	008K08	73.6%	1.3	
4S	036C23	60.3%	2.0	
4S	323C04	49.1%	2.6	
4S	Eu/Het border	39.0%	3.1	
4S	062J03	32.6%	3.4	
4S	209A01	30.8%	3.5	
**4S**	**012B20**	**14.7%**	**4.4**	**S1**
**4S**	**SL_EcoRI0036D15**	**13.6%**	**4.4**	**S3**
**4S**	**018I03**	**1.0%**	**5.0**	**S3**
	Centromere	0.0%	5.1	

4L	SL_EcoRI0027P01	0.3%	5.1	
**4L**	**SL_MboI0036H17**	**0.9%**	**5.2**	**S2**
4L	059C20	8.2%	6.2	
4L	105J24	12.0%	6.7	
4L	030F21	16.1%	7.2	
4L	007D04	16.5%	7.3	
4L	203K18	17.8%	7.4	
**4L**	**SL_MboI0016J15**	**19.9%**	**7.7**	**S2**
**4L**	**SL_MboI0038M16**	**20.1%**	**7.8**	**S4**
4L	006E18	24.5%	8.3	
4L	SL_MboI0078A08	24.8%	8.4	
4L	SL_MboI0120F05	29.6%	9.0	
4L	Eu/Het border	33.0%	9.5	
4L	291H22	37.3%	10.0	
4L	119A16	38.0%	10.1	
4L	020F17	40.7%	10.5	
4L	077O05	44.8%	11.0	
4L	078E04	50.9%	11.8	
4L	208L16	54.4%	12.3	
**4L**	**SL_MboI0029O16**	**54.6%**	**12.3**	**S4**
**4L**	**013P02**	**54.9%**	**12.3**	**S5**
4L	132O11	54.9%	12.3	
4L	171B19	55.5%	12.4	
**4L**	**SL_MboI0018D12**	**57.9%**	**12.7**	**S5**

4L	Chromomere	58.1%	12.8	
**4L**	**020C13**	**59.5%**	**13.0**	**S6**
4L	094K06	59.2%	12.9	
4L	SL_MboI0039E17	65.2%	13.7	
4L	053M02	74.6%	14.9	
4L	008K13	82.3%	16.0	
4L	303A06	95.4%	17.7	
4L	106F07	98.2%	18.1	
**4L**	**SL_MboI0023M21**	**98.9%**	**18.2**	**S6**
4L	Telomere	100.0%	18.3	
5S	Telomere	100.0%	0.0	
**5S**	**SL_s0094J05**	**98.9%**	**0.1**	**S1**
5S	189E17	90.6%	0.7	
5S	309L13	76.6%	1.7	
5S	147F10	67.4%	2.3	
5S	157F14	63.5%	2.6	
5S	298C03	54.7%	3.2	
5S	149J17	40.4%	4.2	
5S	Eu/Het border	40.0%	4.3	
5S	116D11	30.2%	5.0	
5S	282A06	28.3%	5.1	
5S	255N21	27.6%	5.1	
5S	303C11	20.5%	5.6	
5S	107A04	19.1%	5.7	

5S	011G20	7.9%	6.5	
	Centromere	0.0%	7.1	
5L	SL_MboI0058F07	8.5%	7.7	
5L	164O03	10.2%	7.9	
5L	091D14	18.8%	8.5	
5L	033M02	27.6%	9.2	
**5L**	**002J04**	**27.8%**	**9.2**	**S1**
**5L**	**SL_s0031L05**	**29.5%**	**9.3**	**S2**
**5L**	**SL_s0084H11**	**32.4%**	**9.5**	**S2**
**5L**	**016D20**	**34.1%**	**9.7**	**S3**
5L	SL_EcoRI0012L12	34.1%	9.7	
5L	Eu/Het border	40.0%	10.1	
5L	006D05	43.4%	10.4	
5L	169M21	62.6%	11.8	
5L	138J03	72.4%	12.5	
5L	100I16	95.5%	14.3	
5L	251J13	98.0%	14.5	
**5L**	**SL_EcoRI0004N07**	**99.3%**	**14.5**	**S3**
5L	Telomere	100.0%	14.6	
6S	Telomere	100.0%	0.0	
**6S**	**SL_MboI0032G06**	**98.8%**	**0.0**	**S1**
6S	251G05	76.6%	0.9	
6S	250I21	63.7%	1.3	
6S	024L21	61.4%	1.4	

6S	095C08	56.8%	1.6	
6S	SL_MboI0134P07	51.8%	1.8	
6S	Eu/Het border	49.0%	1.9	
6S	008F19	48.9%	1.9	
6S	097D13	39.4%	2.2	
**6S**	**028K02**	**36.4%**	**2.4**	**S1**
6S	SL_EcoRI0056L08	32.6%	2.5	
6S	SL_EcoRI0047D19	31.7%	2.5	
6S	SL_EcoRI0005K11	30.9%	2.6	
6S	SL_EcoRI0031A01	30.8%	2.6	
6S	SL_EcoRI0035D19	30.5%	2.6	
6S	SL_MboI0049I18	29.8%	2.6	
6S	LE_HBa0029E01	29.6%	2.6	
6S	027D17	28.9%	2.6	
6S	176D13	28.7%	2.6	
6S	078K13	28.7%	2.6	
6S	002P09	28.6%	2.6	
6S	SL_MboI0016K18	28.1%	2.7	
**6S**	**025N04**	**27.8%**	**2.7**	**S3**
6S	SL_EcoRI0021L01	27.6%	2.7	
6S	001E08	27.3%	2.7	
6S	IGS-derived satellite	27.1%	2.7	
**6S**	**SL_MboI0029F23**	**26.4%**	**2.7**	**S3**
6S	SL_EcoRI0013G12	25.8%	2.7	

**6S**	**SL_EcoRI0040C12**	**23.9%**	**2.8**	**S2**
6S	SL_MboI0081L19	17.7%	3.0	
6S	068M22	15.2%	4.6	
6S	057J04	2.7%	3.3	
	Centromere	0.0%	3.7	
**6L**	**SL_EcoRI0013C07**	**3.4%**	**4.1**	**S2**
**6L**	**SL_MboI0029C07**	**3.9%**	**4.2**	**S4**
6L	031J01	5.6%	4.4	
6L	SL_MboI0034N14	6.1%	4.5	
6L	188N10	13.2%	5.4	
**6L**	**SL_EcoRI0009J17**	**16.5%**	**5.8**	**S4**
**6L**	**SL_EcoRI0033O20**	**16.7%**	**5.8**	**S5**
6L	SL_MboI0018M19	16.9%	5.8	
6L	SL_EcoRI0040G06	17.7%	5.9	
**6L**	**031J01**	**17.9%**	**6.0**	**S5**
**6L**	**SL_MboI0034N14**	**18.1%**	**6.0**	**S6**
6L	SL_MboI0029D03	18.7%	6.1	
6L	SL_EcoRI0001M14	19.8%	6.2	
6L	SL_EcoRI0027F24	19.9%	6.2	
6L	055B14	20.9%	6.3	
6L	Eu/Het border	21.0%	6.4	
**6L**	**SL_EcoRI0023E10**	**37.1%**	**8.4**	**S6**
**6L**	**SL_EcoRI0018M04**	**39.2%**	**8.7**	**S7**
6L	SL_MboI0030C09	57.0%	10.9	

6L	081D06	60.1%	11.3	
6L	146O07	80.6%	13.9	
**6L**	**SL_EcoRI0008B16**	**88.2%**	**14.9**	**S7**
**6L**	**LE_HBa0025F06**	**90.0%**	**15.1**	**S8**
6L	068M14	91.0%	15.3	
6L	184G14	96.1%	15.9	
6L	098L02	98.1%	16.2	
**6L**	**SL_EcoRI0019F04**	**99.5%**	**16.3**	**S8**
6L	Telomere	100.0%	16.4	
7S	Telomere	100.0%	0.0	
7S	193E01	99.1%	0.1	
**7S**	**SL_EcoRI0006L14**	**98.0%**	**0.1**	**S1**
7S	002D20	97.5%	0.1	
7S	095C18	97.1%	0.2	
7S	007H24	96.6%	0.2	
7S	111F22	95.5%	0.3	
7S	150H03	94.5%	0.3	
7S	SL_EcoRI0042G10	83.0%	1.0	
7S	SL_EcoRI0025E06	57.1%	2.4	
7S	SL_MboI0137E08	52.0%	2.7	
7S	173A21	50.2%	2.8	
7S	SL_EcoRI0008H22	47.2%	3.0	
7S	SL_MboI0024O10	46.3%	3.0	
7S	325D07	44.0%	3.1	

7S	Eu/Het border	40.0%	3.4	
7S	293I23	33.0%	3.8	
	Centromere	0.0%	5.6	
**7L**	**SL_MboI0016H01**	**10.0%**	**6.6**	**S1**
**7L**	**032J14**	**10.6%**	**6.7**	**S2**
7L	003K20	13.6%	7.0	
7L	082D04	15.4%	7.2	
7L	027F11	19.3%	7.6	
7L	SL_EcoRI0021M11	19.5%	7.6	
**7L**	**SL_EcoRI0016C19**	**19.7%**	**7.6**	**S2**
7L	SL_EcoRI0041L20	19.9%	7.6	
7L	SL_EcoRI0041L23	19.9%	7.6	
7L	042K23	20.5%	7.7	
7L	036D05	21.1%	7.8	
7L	025A06	21.3%	7.8	
7L	SL_MboI0017P20	21.5%	7.8	
7L	SL_MboI0017I04	21.5%	7.8	
**7L**	**001O15**	**22.5%**	**7.9**	**S3**
7L	012C17	23.1%	8.0	
7L	034B22	27.8%	8.5	
7L	188B22	29.9%	8.7	
7L	224G23	32.1%	8.9	
7L	Eu/Het border	34.0%	9.1	
7L	041L08	36.3%	9.3	

7L	106F06	38.4%	9.6	
7L	SL_MboI0141H03	40.5%	9.8	
7L	SL_EcoRI0101G19	42.5%	10.0	
7L	SL_EcoRI0095F20	75.0%	13.3	
**7L**	**SL_EcoRI0032D11**	**77.5%**	**13.6**	**S3**
**7L**	**024C10**	**78.8%**	**13.7**	**S4**
7L	308M01	83.0%	14.1	
7L	079F09	84.0%	14.2	
7L	227C07	86.2%	14.5	
7L	178O02	88.7%	14.7	
7L	130B18	89.0%	14.8	
7L	226J04	89.4%	14.8	
7L	059P18	90.3%	14.9	
7L	261J23	91.9%	15.1	
7L	167K07	98.0%	15.7	
7L	179K09	98.0%	15.7	
**7L**	**SL_MboI0017L19**	**98.2%**	**15.7**	**S4**
7L	215P04	99.7%	15.9	
7L	Telomere	100.0%	15.9	
8S	Telomere	100.0%	0.0	
**8S**	**030O09**	**98.5%**	**0.1**	**S1**
8S	025I17	87.1%	0.6	
8S	216M19	85.9%	0.7	
8S	270A17	82.8%	0.8	

**8S**	**008J16**	**68.5%**	**1.5**	**S1**
**8S**	**015E06**	**62.8%**	**1.8**	**S2**
8S	Eu/Het border	45.0%	2.7	
**8S**	**009K19**	**38.5%**	**3.0**	**S2**
8S	SL_EcoRI0002D11	36.5%	3.1	
8S	LE_HBa0041G04	35.7%	3.2	
**8S**	**SL_MboI0012I24**	**35.2%**	**3.2**	**S7**
8S	SL_MboI0041D13	34.9%	3.2	
8S	IGS-derived satellite	34.6%	3.2	
**8S**	**SL_s0093G06**	**33.0%**	**3.3**	**S5**
**8S**	**SL_s0030B23**	**28.3%**	**3.5**	**S5**
**8S**	**SL_s0105O13**	**27.4%**	**3.6**	**S6**
	Centromere	0.0%	4.9	
8L	SL_EcoRI0008F12	3.1%	5.3	
8L	SL_MboI0012P13	3.7%	5.3	
**8L**	**SL_MboI0005G10**	**7.7%**	**5.8**	**S6**
**8L**	**SL_s0078C24**	**8.0%**	**5.8**	**S3**
8L	213E05	11.8%	6.3	
**8L**	**SL_s0058J24**	**17.0%**	**6.9**	**S3**
**8L**	**SL_EcoRI0024O12**	**18.2%**	**7.0**	**S4**
8L	034L18	23.4%	7.6	
8L	SL_MboI0019D14	24.6%	7.8	**S4**
8L	SL_MboI0012P13	25.7%	7.9	
8L	033A16	26.0%	7.9	**S8**

8L	027D09	27.6%	8.1	
8L	SL_EcoRI0027A06	31.4%	8.6	
8L	SL_EcoRI0039B17	32.0%	8.6	
8L	Eu/Het border	32.0%	8.6	
8L	160L02	32.1%	8.7	
8L	225C20	33.1%	8.8	
8L	076I13	38.3%	9.4	
8L	Chromomere	58.6%	11.8	
**8L**	**SL_MboI0007C12**	**58.7%**	**11.8**	**S8**
**8L**	**SL_s0082B11**	**60.2%**	**11.9**	**S9**
8L	005L01	99.6%	16.6	
8L	SL_EcoRI0040H01	99.7%	16.6	**S9**
8L	Telomere	100.0%	16.6	
9S	Telomere	100.0%	0.0	
**9S**	**SL_MboI0032A02**	**99.4%**	**0.0**	**S1**
9S	026I24	99.3%	0.0	
9S	072G22	88.9%	0.6	
9S	116C14	84.9%	0.8	
9S	168F14	77.9%	1.2	
9S	203J14	77.6%	1.3	
9S	300E15	64.5%	2.0	
9S	026P14	63.4%	2.1	
9S	255E01	56.9%	2.4	
9S	SL_MboI0080E11	48.9%	2.9	

9S	300E15	47.5%	2.9	
9S	026P14	47.3%	3.0	
9S	308C20	41.6%	3.3	
9S	Eu/Het border	40.0%	3.4	
9S	197E10	28.3%	4.0	
9S	SL_MboI0056H18	9.4%	5.1	
9S	072H18	6.6%	5.2	
**9S**	**SL_MboI0025N23**	**5.8%**	**5.3**	**S1**
9S	SL_MboI0045G03	3.4%	5.4	
9S	SL_EcoRI0052D12	3.4%	5.4	
**9S**	**SL_EcoRI0015A15**	**2.3%**	**5.5**	**S4**
	Centromere	0.0%	5.6	
**9L**	**SL_EcoRI0025E12**	**7.2%**	**6.3**	**S4**
**9L**	**SL_EcoRI0018E08**	**8.2%**	**6.4**	**S3**
9L	061J21	11.0%	6.7	
9L	075M11	11.8%	6.8	
**9L**	**LE_HBa0005G01**	**13.5%**	**7.0**	**S3**
**9L**	**SL_MboI0013P10**	**15.0%**	**7.1**	**S5**
**9L**	**SL_EcoRI0022O16**	**15.3%**	**7.2**	**S5**
**9L**	**SL_MboI0018I03**	**16.9%**	**7.3**	**S6**
9L	107D15	31.3%	8.8	
9L	Eu/Het border	34.0%	9.1	
9L	099F14	61.2%	11.9	
**9L**	**LE_HBa0005M15**	**62.7%**	**12.1**	**S6**

9L	107P11	63.7%	12.2	
**9L**	**SL_FOS0051C04**	**64.2%**	**12.2**	**S2**
**9L**	**SL_MboI0019G06**	**64.6%**	**12.3**	**S7**
9L	099P03	70.7%	12.9	
9L	278J12	71.6%	13.0	
9L	248I10	72.3%	13.0	
**9L**	**SL_MboI0020M04**	**74.5%**	**13.3**	**S7**
**9L**	**SL_MboI0025N11**	**77.9%**	**13.6**	**S8**
**9L**	**LE_HBa0010F04**	**81.6%**	**14.0**	**S8**
**9L**	**LE_HBa0001I06**	**82.7%**	**14.1**	**S9**
9L	245E05	88.2%	14.7	
**9L**	**SL_EcoRI0024O21**	**91.9%**	**15.1**	**S9**
**9L**	**SL_MboI0037I08**	**95.3%**	**15.4**	**S10**
9L	165P17	95.4%	15.4	
9L	109D11	97.5%	15.6	
**9L**	**SL_MboI0022L14**	**99.2%**	**15.8**	**S10**
9L	SL_MboI0042F10	99.6%	15.9	
9L	Telomere	100.0%	15.9	
10S	Telomere	100.0%	0.0	
**10S**	**SL_EcoRI0027L04**	**98.5%**	**0.1**	**S1**
10S	060A06	91.6%	0.4	
10S	091N19	88.4%	0.6	
10S	012D17	87.3%	0.6	
10S	176H22	87.2%	0.6	

10S	155I21	76.8%	1.1	
10S	222H10	75.4%	1.2	
10S	SL_EcoRI0076M19	70.5%	1.4	
10S	028O04	69.9%	1.4	
10S	041K23	56.3%	2.1	
**10S**	**SL_EcoRI0009D07**	**52.3%**	**2.3**	**S1**
**10S**	**023E16**	**51.7%**	**2.3**	**S2**
10S	201C12	50.7%	2.4	
10S	Eu/Het border	46.0%	2.6	
10S	205L07	30.5%	3.3	
10S	SL_MboI0020C04	27.5%	3.5	
10S	036L10	23.2%	3.7	
10S	111D09	19.1%	3.9	
10S	105C09	17.5%	4.0	
10S	SL_EcoRI0040D01	14.5%	4.1	
**10S**	**SL_s0071N16**	**11.3%**	**4.3**	**S2**
10S	237A02	10.8%	4.3	
**10S**	**SL_EcoRI0029F05**	**9.6%**	**4.3**	**S4**
	Centromere	0.0%	4.8	
**10L**	**SL_MboI0011N01**	**6.2%**	**5.4**	**S4**
**10L**	**SL_s0042K13**	**6.5%**	**5.5**	**S3**
10L	115K16	10.8%	5.9	
10L	114B06	16.6%	6.5	
10L	043B12	26.3%	7.5	

10L	037N23	26.4%	7.5	
10L	Eu/Het border	33.0%	8.1	
10L	044O20	39.6%	8.8	
10L	045L17	43.8%	9.2	
10L	053L19	49.1%	9.8	
**10L**	**SL_s0121P17**	**53.2%**	**10.2**	**S3**
**10L**	**SL_FOS0014E24**	**54.3%**	**10.3**	**S5**
**10L**	**SL_EcoRI0036N16**	**55.3%**	**10.4**	**S6**
10L	SL_EcoRI0013B18	56.3%	10.5	
10L	204I05	57.7%	10.6	
10L	189B10	58.5%	10.7	
10L	022B11	58.7%	10.7	
10L	044H12	61.7%	11.0	
10L	188N09	62.1%	11.1	
10L	071D20	62.9%	11.1	
10L	020A12	63.1%	11.2	
10L	248A13	63.5%	11.2	
10L	234C10	64.1%	11.3	
10L	156C03	64.6%	11.3	
10L	057G01	71.3%	12.0	
10L	206B16	71.8%	12.0	
10L	005D06	76.1%	12.5	
10L	256L16	78.5%	12.7	
10L	021O12	79.2%	12.8	

10L	334K22	82.8%	13.2	
10L	049K02	86.1%	13.5	
10L	011E16	89.9%	13.9	
10L	181E17	94.7%	14.4	
10L	013B20	96.0%	14.5	
**10L**	**SL_EcoRI0008A07**	**99.6%**	**14.9**	**S6**
10L	Telomere	100.0%	14.9	
11S	Telomere	100.0%	0.0	
**11S**	**SL_s0029B21**	**98.6%**	**0.1**	**S1**
11S	168B23	97.2%	0.2	
11S	024K09	63.9%	2.3	
11S	027B05	63.6%	2.4	
11S	015A13	58.0%	2.7	
11S	SL_MboI121I03	42.2%	3.8	
11S	034I10	41.8%	3.8	
11S	214E16	41.4%	3.8	
11S	080C09	41.0%	3.8	
11S	128G07	40.5%	3.9	
11S	064J13	39.9%	3.9	
11S	291F09	39.2%	4.0	
11S	SL_MboI0052K14	37.7%	4.1	
11S	Eu/Het border	32.0%	4.4	
**11S**	**SL_EcoRI0018D15**	**17.9%**	**5.3**	**S1**
**11S**	**008F06**	**16.7%**	**5.4**	**S4**

11S	023H04	13.7%	5.6	
**11S**	**004F15**	**11.7%**	**5.7**	**S4**
11S	IGS-derived satellite	11.0%	5.8	
**11S**	**SL_s0082L15**	**10.1%**	**5.8**	**S2**
	Centromere	0.0%	6.5	
11L	305L18	6.6%	7.0	
11L	187P23	7.4%	7.1	
11L	SL_EcoRI0045G19	8.0%	7.1	
11L	SL_EcoRI0027A06	12.6%	7.5	
**11L**	**001N12**	**13.7%**	**7.6**	**S2**
11L	SL_MboI0047F15	14.3%	7.6	
11L	062E02	15.2%	7.7	
**11L**	**SL_EcoRI0031L09**	**16.2%**	**7.8**	**S3**
11L	Eu/Het border	43.0%	9.9	
**11L**	**SL_s0045H10**	**44.4%**	**10.1**	**S3**
**11L**	**SL_s0084O21**	**45.6%**	**10.1**	**S5**
11L	Mitochondrial DNA	47.3%	10.3	
**11L**	**SL_s0017L16**	**48.1%**	**10.3**	**S5**
**11L**	**SL_s0105E01**	**50.4%**	**10.5**	**S6**
11L	245M17	50.7%	10.6	
11L	158K02	51.5%	10.6	
11L	119D16	56.6%	11.0	
11L	316E10	58.3%	11.2	
11L	249E07	66.8%	11.8	

11L	323E19	88.2%	13.6	
**11L**	**SL_s0014A19**	**98.3%**	**14.4**	**S6**
11L	Telomere	100.0%	14.5	
12S	Telomere	100.0%	0.0	
**12S**	**SL_MboI0030P17**	**99.3%**	**0.0**	**S1**
12S	045N22	96.8%	0.2	
12S	026C13	93.7%	0.4	
12S	163O04	82.7%	1.2	
12S	146I19	78.9%	1.5	
12S	049J09	69.3%	2.2	
12S	075A23	67.9%	2.3	
12S	180J10	65.9%	2.4	
12S	206N09	64.7%	2.5	
12S	105C12	56.9%	3.1	
12S	154D06	50.4%	3.5	
12S	Eu/Het border	40.0%	4.3	
**12S**	**SL_EcoRI0005K14**	**38.9%**	**4.3**	**S1**
**12S**	**LE_HBa0016B06**	**37.9%**	**4.4**	**S8**
12S	SL_MboI0050G12	36.9%	4.5	
**12S**	**SL_MboI0038L04**	**36.3%**	**4.5**	**S8**
12S	SL_EcoRI0010P04	35.8%	4.6	
**12S**	**SL_EcoRI0036O23**	**35.7%**	**4.6**	**S7**
**12S**	**SL_EcoRI0003D15**	**31.1%**	**4.9**	**S7**
**12S**	**SL_MboI0008A07**	**29.9%**	**5.0**	**S2**

**12S**	**003H12**	**22.2%**	**5.5**	**S2**
**12S**	**SL_MboI0023C14**	**21.0%**	**5.6**	**S6**
**12S**	**SL_EcoRI0004B02**	**18.9%**	**5.8**	**S6**
**12S**	**SL_MboI0040D19**	**17.6%**	**5.9**	**S5**
**12S**	**SL_MboI0019O03**	**7.7%**	**6.6**	**S3**
12S	005D14	7.0%	6.6	
**12S**	**SL_EcoRI0001K10**	**4.2%**	**6.8**	**S5**
12S	SL_EcoRI0012H19	3.4%	6.9	
12S	012P02	0.0%	7.1	
	Centromere	0.0%	7.1	
12L	SL_EcoRI0012H19	2.2%	7.3	**Heinz 1706 S5**
12L	005D14	3.7%	7.4	**Heinz 1706 S3**
12L	153L10	7.4%	7.7	
12L	148K11	16.1%	8.3	
12L	006G23	23.6%	8.9	
12L	047D08	27.6%	9.2	
12L	SL_MboI0009A03	29.5%	9.3	
12L	SL_MboI0128K09	31.6%	9.5	
12L	331D02	32.1%	9.5	
12L	152A18	35.4%	9.8	
12L	Eu/Het border	40.0%	10.1	
**12L**	**SL_MboI0035N06**	**60.2%**	**11.6**	**S3**
**12L**	**SL_MboI0006L17**	**61.0%**	**11.7**	**S4**

**12L**	**017P17**	**65.1%**	**12.0**	**S4**
12L	079O22	66.6%	12.1	
12L	SL_MboI0011A16	69.3%	12.3	
**12L**	**SL_EcoRI0031M18**	**73.1%**	**12.6**	**S9**
12L	012E19	76.4%	12.8	
**12L**	**SL_EcoRI0024H05**	**79.0%**	**13.0**	**S9**
**12L**	**LE_HBa0029L21**	**80.6%**	**13.1**	**S10**
12L	SL_EcoRI0045D23	81.0%	13.2	
12L	326K10	82.4%	13.3	
12L	093P12	87.0%	13.6	
12L	055G18	95.7%	14.3	
12L	116D04	97.9%	14.4	
**12L**	**LE_HBa0030J22**	**98.7%**	**14.5**	**S10**
12L	Telomere	100.0%	14.6	

Table S4   FISH-based scaffold order, orientation, size, and chromatin type

Chr.	Scaffold Name[1,2]	Scaffold Number[2,3]	Left (head)[4] BAC Probe[5]	Right (tail)[4] BAC Probe[5]	Scaffold Size (Mb)[6]	SC len. (µm)	Chromatin Type[11]
1	SL2.40sc04133	1	s090M22	M034D03	33.0	5.8	Eu/Het
1	SL2.40sc04191	3	s053P14	s083L21	1.7	0.2	Het
1	SL2.40sc03666	2	E016I11	H033N15	2.6	0.2	Het
1	SL2.40sc03594	4	s006A13	s042B18	6.3	0.7	Het
1	SL2.40sc05010	5	s042O02	H037N04	20.5	2.9	Het/Border
1	SL2.40sc05941	6	s040G18	E021C24	6.2	1.7	Eu/Border
1	SL2.40sc06903	8	s022L14	s121I01	2.7	1.1	Eu
1	SL2.40sc06917	7	s071P10	n/a[7]	0.4	n/a	Eu
1	SL2.40sc04323	9	s024J19	M028C09	17.0	12.6	Eu
2	SL2.40sc04732	4	E007F19	E008B15	1.7	0.2	Het
2	SL2.40sc04208	6	s053E24	s044P10	1.4	0.2	Het
2	SL2.40sc05776	5	H011G24	M036C03	2.1	0.5	Het
2	SL2.40sc06593	1	s056I16	s079J24	4.0	0.5	Het
2	SL2.40sc04142	2	s053C07	s050E09	6.5	1.5	Het/Cent
2	SL2.40sc03766	3	M021A03	E034J17	11.7	2.5	Het/Border
2	SL2.40sc03665	7	s101F18	M017J13	22.4	15.3	Eu
3	SL2.40sc04439	1	s009C01	s086D22	2.5	1.4	Eu
3	SL2.40sc04696	4	s018K15	s002G24	1.3	0.5	Eu
3	SL2.40sc05330	5	s050E05	H037B06	2.6	0.9	Eu/Border
3	SL2.40sc04126	6	H020G20	H039C15	6.5	0.9	Het/Border
3	SL2.40sc04616	9	M079O10	M002J15	3.1	0.6	Cent/Het
3	SL2.40sc06725	7	H244B01	E121G21	3.0	0.3	Het
3	SL2.40sc04704	8	H027L13	s071G06	18.6	2.2[8]	Het
3	SL2.40sc03721	12	E018K21	H028L03	1.1	0.0[8]	Het/Border
3	SL2.40sc04822	2	s042B23	H030A19	4.1	0.5[8]	Het/Border
3	SL2.40sc03806	10	H011C02	H001H05	5.5	1.8[8]	Border/Eu
3	SL2.40sc03796	11	M003H09	E031G05	7.0	2.4	Eu
3	SL2.40sc06911	3	M015G06	s048H23	0.4	0.0	Eu
3	SL2.40sc03701	13	E006F17	E034G21	9.2	6.2	Eu
4	SL2.40sc03604	1	M035I14	H012B20	11.6	4.2	Eu/Het
4	SL2.40sc05339	3	E036C15	H018I03	2.0	0.6	Het/Cent
4	SL2.40sc03683	2	M036H17	M016J15	17.6	2.5	Cent/Het
4	SL2.40sc06101	4	M038M16	M029O16	23.3	4.6	Het/Eu
4	SL2.40sc04680	5	H013P02	M018D12	0.8	0.4	Eu

4	SL2.40sc04135	6	H020C13	M023M21	8.8	5.2	Eu
5	SL2.40sc03726	1	s094J05	H002J04	42.1	9.1	Eu/Het/Cent
5	SL2.40sc06155	2	s031L05	s084H11	4.3	0.2	Het
5	SL2.40sc03902	3	H016D20	E004N07	18.6	4.9	Eu/Het
6	SL2.40sc04474	1	M032G6	H028K02	8.6	2.3	Eu/Het
6	SL2.40sc06140	3	H025N04	M029F23	0.7	0.1	Het
6	SL2.40sc05383	2	E040C12	E013C07	6.1	1.3	Het/Cent
6	SL2.40sc04279	4	M029C07	E009J17	9.6	1.6	Het
6	SL2.40sc05188	5	E033O20	H031J01	2.4	0.2	Border
6	SL2.40sc05732	6	M034N10	E023E10	6.6	2.4	Border/Eu
6	SL2.40sc05054	7	E018M04	E008B16	10.3	6.2	Eu
6	SL2.40sc03622	8	H025F06	E019F04	1.8	1.2	Eu
7	SL2.40sc03731	1	E006L14	M016H01	33.3	6.5	Eu/Het/Cent
7	SL2.40sc05397	2	H032J14	E016C19	8.0	0.9	Het
7	SL2.40sc03685	3	H001O15	E032D11	20.7	5.7	Het/Eu
7	SL2.40sc04626	4	H024C01	M017L19	3.4	2.0	Eu
8	SL2.40sc04813	1	H030O09	H008J16	3.0	1.5	Eu
8	SL2.40sc03770	2	H015E06	H009K19	6.3	1.2	Eu/Border
8	SL2.40sc04167	7	M012I24	n/a[7]	0.2	n/a	Het
8	SL2.40sc03749	5	s093G06	s030B23	1.5	0.2	Het
8	SL2.40sc04236	6	s105O13	M005G10	14.6	2.2	Het/Cent
8	SL2.40sc03835	3	s078C24	s058J24	8.4	1.1	Het
8	SL2.40sc04701	4	E024O12	M019D14	7.0	0.7	Het
8	SL2.40sc04948	8	H033A16	M007C12	14.4	3.8	Eu/Het
8	SL2.40sc03923	9	s082B11	E040H01	7.5	4.6	Eu
9	SL2.40sc03771	1	M032A02	M025N23	19.2	5.2	Eu/Het
9	SL2.40sc04008	4	E015A15	E025E12	5.2	0.9	Het/Cent
9	SL2.40sc04950	3	E018E08	M005G01	5.8	0.5	Het
9	SL2.40sc04785	5	M013P10	E022O16	2.0	0.0	Het
9	SL2.40sc04777	6	M018I03	H005M15	28.2	4.7	Eu/Het
9	SL2.40sc06916	2	F051C04	n/a[7]	0.0[9]	n/a	Eu
9	SL2.40sc05269	7	M019G06	M020M04	2.7	1.0	Eu
9	SL2.40sc03852	8	M025N11	H010F04	1.3	0.4	Eu
9	SL2.40sc04828	9	H001I06	E024O21	2.5	0.9	Eu
9	SL2.40sc06214	10	M037I08	M022L14	0.6	0.4	Eu
10	SL2.40sc05925	1	E027L04	E009D07	5.4	2.2	Eu/Border
10	SL2.40sc03798	2	H023E16	s071N16	16.5	1.9	Het
10	SL2.40sc04872	4	E029F05	M011N01	3.9	1.1	Cent
10	SL2.40sc05632	3	s042K13	s121P17	31.1	4.7	Eu/Het

10	SL2.40sc04534	5	F014E24	n/a[7]	0.1	n/a	Eu
10	SL2.40sc04199	6	E036N16	E008A07	8.0	4.5	Eu
11	SL2.40sc03748	1	s029B21	E018D15	15.2	5.2	Eu/Het
11	SL2.40sc06763	4	H008F06	H004F15	2.5	0.3	Het
11	SL2.40sc04054	2	s082L15	H001N12	8.0	1.8	Het/Cent
11	SL2.40sc03752	3	E031L09	s045H10	17.4	2.3	Het/Border
11	SL2.40sc06137	5	s084O21	s017L16	2.1	0.2	Border
11	SL2.40sc03876	6	s105E01	s014A19	8.2	3.8	Eu
12	SL2.40sc04607	1	M030P17	E005K14	16.1	4.3	Eu/Border
12	SL2.40sc06147	8	H016B06	M038L04	1.2	0.1	Border
12	SL2.40sc04039	7	E036O23	E003D15	4.9	0.3	Border
12	SL2.40sc04878	2	M008A07	H003H12	5.7	0.5	Het
12	SL2.40sc04266	6	M023C14	E004B02	1.3	0.1	Het
12	SL2.40sc04757[10]	5	M040D19	E012H19	5.8	1.4	Het/Cent
12	SL2.40sc04057	3	H005D14	M035N06	25.2	4.2	Eu/Het
12	SL2.40sc04915	4	M006L17	H017P17	1.6	0.3	Eu
12	SL2.40sc05611	9	E031M18	E024H05	1.2	0.4	Eu
12	SL2.40sc05380	10	H029L21	E030J22	2.6	1.4	Eu
				Total:	760.0	196.7	

[1] http://solgenomics.net/cview/map.pl?map_id=agp

[2] FISH-based scaffolds are color coded with reference to a comparison with their order and orientation determined using the Kazusa EXPEN 2000 linkage map. Scaffold names and numbers that are unchanged by FISH are shown with black lettering on a white background. Changed scaffolds are shown with white lettering on a red background (order only), white lettering on a blue background (orientation only), and white lettering on a purple background (both order and orientation).

[3] Scaffold number is determined by scaffold order from head to tail in the pseudomolecules based on the linkage map.

[4] Left and right refer to the probe orientation when the chromosomes are diagramed with the telomere of the short arm on the left (= head) and the telomere of the long arm on the right (= tail).

[5] BAC library names have been abbreviated. H=HindIII, E=EcoRI, M=MboI, s=sheared, F=Fosmid

[6] The Tomato Genome Consortium, 2012 The tomato genome sequence provides insights into fleshy fruit evolution. Nature 485: 635-641; http://solgenomics.net

[7] Not applicable because only one BAC/fosmid was localized in this small scaffold.

[8] SC length determinations are relatively inaccurate in and near scaffolds 12 and 2.

[9] The actual length of this scaffold is 17.6 Kb so it is rounded to 0.0 Mb.

[10] Scaffold SL2.40sc04757 was ordered and oriented in Heinz 1706 because this scaffold was involved in an inversion around the kinetochore of Cherry Tomato LA4444.

11 Eu = euchromatin; Het = heterochromatin; cent = centromere/kinetochore; Border (transition between euchromatin and heterochromatin); Eu/Het includes euchromatin, heterochromatin, and the border between Eu and Het; Het/Cent includes heterochromatin and centromere; Border/Eu includes euchromatin and border with heterochromatin; Eu/Het/Cent includes euchromatin, the border with heterochromatin, heterochromatin and centromeres.

Table S5 Comparison of scaffold order and orientation based on linkage, optical mapping, and FISH

				Scaffold Order According to:			
Chromosome	Scaffold No.[1]	Scaffold Name[2]	Mb Length	Linkage	Optical Superscaffold (OS)[3]		FISH[4]
1	1	SL2.40sc04133	33.0	1			1
1	2	SL2.40sc03666	1.7	2			3
1	3	SL2.40sc04191	2.6	3	2	OS 1	2*
1	4	SL2.40sc03594	6.3	4	4		4
1	5	SL2.40sc05010	20.5	5	5		5
1	6	SL2.40sc05941	6.2	6	6	OS 2	6
1	7	SL2.40sc06903	2.7	7	8		8
1	8	SL2.40sc06917	0.4	8	7		7
1	9	SL2.40sc04323	17.0	9			9
2	1	SL2.40sc06593	1.7	1	4	OS 3	4*
2	2	SL2.40sc04142	1.4	2	6		6*
2	3	SL2.40sc03766	2.1	3			5
2	4	SL2.40sc04732	4.0	4			1
2	5	SL2.40sc05776	6.5	5			2
2	6	SL2.40sc04208	11.7	6			3
2	7	SL2.40sc03665	22.4	7			7
3	1	SL2.40sc04439	2.5	1			1
3	2	SL2.40sc04822	1.3	2			4
3	3	SL2.40sc06911	2.6	3			5
3	4	SL2.40sc04696	6.5	4			6
3	5	SL2.40sc05330	3.1	5			9
3	6	SL2.40sc04126	3.0	6			7
3	7	SL2.40sc06725	18.6	7			8
3	8	SL2.40sc04704	4.1	8			2*
3	9	SL2.40sc04616	1.1	9			12
3	10	SL2.40sc03806	5.5	10			10
3	11	SL2.40sc03796	7.0	11			11
3	12	SL2.40sc03721	0.4	12	3	OS 4	3*
3	13	SL2.40sc03701	9.2	13	13		13
4	1	SL2.40sc03604	11.6	1			1
4	2	SL2.40sc03683	2.0	2			3*
4	3	SL2.40sc05339	17.6	3			2*
4	4	SL2.40sc06101	23.3	4			4

4	5	SL2.40sc04680	0.8	5			5
4	6	SL2.40sc04135	8.8	6			6
5	1	SL2.40sc03726	42.1	1			1
5	2	SL2.40sc06155	4.3	2			2*
5	3	SL2.40sc03902	18.6	3			3
6	1	SL2.40sc04474	8.6	1			1
6	2	SL2.40sc05383	0.7	2			3*
6	3	SL2.40sc06140	6.1	3			2
6	4	SL2.40sc04279	9.6	4			4
6	5	SL2.40sc05188	2.4	5	5		5*
6	6	SL2.40sc05732	6.6	6	6	OS 5	6
6	7	SL2.40sc05054	10.3	7	7		7
6	8	SL2.40sc03622	1.8	8	8		8
7	1	SL2.40sc03731	33.3	1	1	OS 6	1
7	2	SL2.40sc05397	8.0	2	2		2
7	3	SL2.40sc03685	20.7	3			3
7	4	SL2.40sc04626	3.4	4			4
8	1	SL2.40sc04813	3.0	1	1	OS 7	1
8	2	SL2.40sc03770	6.3	2	2		2
8	3	SL2.40sc03835	0.2	3			7
8	4	SL2.40sc04701	1.5	4	5		5*
8	5	SL2.40sc03749	14.6	5	6	OS 8	6*
8	6	SL2.40sc04236	8.4	6	3		3*
8	7	SL2.40sc04167	7.0	7	4		4*
8	8	SL2.40sc04948	14.4	8			8
8	9	SL2.40sc03923	7.5	9			9
9	1	SL2.40sc03771	19.2	1			1
9	2	SL2.40sc06916	0.0	2	4		4
9	3	SL2.40sc04950	5.2	3	3	OS 9	3*
9	4	SL2.40sc04008	5.8	4	5		5
9	5	SL2.40sc04785	2.0	5	6		6
9	6	SL2.40sc04777	28.2	6			2
9	7	SL2.40sc05269	2.7	7			7
9	8	SL2.40sc03852	1.3	8	8		8
9	9	SL2.40sc04828	2.5	9	9	OS 10	9
9	10	SL2.40sc06214	0.6	10	10		10
10	1	SL2.40sc05925	5.4	1	1	OS 11	1
10	2	SL2.40sc03798	16.5	2	2		2
10	3	SL2.40sc05632	3.9	3			4

10	4	SL2.40sc04872	31.1	4			3
10	5	SL2.40sc04534	0.1	5			5
10	6	SL2.40sc04199	8.0	6			6
11	1	SL2.40sc03748	15.2	1			1
11	2	SL2.40sc04054	2.5	2			4
11	3	SL2.40sc03752	8.0	3			2
11	4	SL2.40sc06763	17.4	4			3*
11	5	SL2.40sc06137	2.1	5	**5**	OS 12	5
11	6	SL2.40sc03876	8.2	6	**6**		6
12	1	SL2.40sc04607	16.1	1			1
12	2	SL2.40sc04878	1.2	2			8
12	3	SL2.40sc04057	4.9	3			7*
12	4	SL2.40sc04915	5.7	4			2
12	5	SL2.40sc04757	1.3	5			6
12	6	SL2.40sc04266	5.8	6	**5**	OS 13	5
12	7	SL2.40sc04039	25.2	7	**3**		3
12	8	SL2.40sc06147	1.6	8	**4**		4
12	9	SL2.40sc05611	1.2	9	**9**	OS 14	9
12	10	SL2.40sc05380	2.6	10	**10**		10
**Total**			760.0				

[1]Scaffolds are numbered according to their order determined using the tomato Kazusa EXPEN 2000 linkage map at http://solgenomics.net/cview/map.pl?map_version_id=103

[2] http://solgenomics.net/cview/map.pl?map_id=agp

[3]Optical superscaffolds consist of 2-4 sequenced scaffolds ordered and oriented by optical mapping. Scaffold numbers refer to column 2. Empty spaces indicate scaffolds that were not ordered and oriented by optical mapping. Green scaffolds are in the same order and orientation as that determined by the linkage map. Red scaffolds are in the same orientation but different order, blue scaffolds are in the same order but different orientation, and purple scaffolds are in a different order and orientation than that determined by the linkage map.

[4]Order and orientation of scaffolds determined by FISH. Scaffold numbers refer to column 2. Green scaffolds are in the same order and orientation by both optical mapping and by FISH.

*Denotes scaffolds with a different orientation by FISH than that determined by the linkage map.

Table S6 Estimating the linear density of DNA in euchromatin, heterochromatin, and kinetochores by FISH[1]

Chromatin Type	Chr. #	Scaffold #	Scaffold Size Mb (Corrected Mb)[2]	SC Length - µm	Mb/µm
Euchromatin	1	SL2.40sc04323	17.0 (16.8)	12.6	1.33
	2	SL2.40sc03665	22.4 (21.8)	15.3	1.43
	3	SL2.40sc04439	2.5 (2.3)	1.4	1.62
	3	SL2.40sc03701	9.2 (8.7)	6.2	1.40
	4	SL2.40sc04135	8.8 (8.6)	5.2	1.65
	6	SL2.40sc05054	10.3 (9.8)	6.2	1.58
	6	SL2.40sc03622	1.8 (1.7)	1.2	1.38
	7	SL2.40sc04626	3.4 (3.2)	2.0	1.58
	8	SL2.40sc03923	7.5 (7.4)	4.6	1.60
	10	SL2.40sc04199	8.0 (7.7)	4.5	1.70
	11	SL2.40sc03876	8.2 (7.9)	3.8	2.07
	12	SL2.40sc05380	2.6 (2.3)	1.4	1.66
	**Total**		**101.7 (98.0) Mb**	**64.4 µm**	**1.52 Mb/µm[3]**
Heterochromatin	1	SL2.40sc03594	6.3 (5.9)	0.7	8.41
	7	SL2.40sc05397	8.0 (7.8)	0.9	8.69
	8	SL2.40sc04701	7.0 (6.8)	0.7	9.76
	10	SL2.40sc03798	16.5 (16.3)	1.9	8.58
	**Total**		**37.8 (36.8) Mb**	**4.2 µm**	**8.76 Mb/µm[3]**
Kinetochore	10	SL2.40sc04872	3.9 (3.6)	1.1	3.27
	**Total**		**3.9 (3.6) Mb**	**1.1 µm**	**3.27 Mb/µm[3]**

[1]Scaffold numbers, DNA amounts in scaffolds, lengths of scaffolds, and chromatin types were extracted from Table S1.

[2]The FISH signals at either end of a scaffold are not at the very ends of the scaffold but somewhat into the scaffold [see File S1, Additional Materials and Methods- Determining the amount of DNA per micrometer of SC (= linear DNA density) in kinetochores, euchromatin, and heterochromatin]. Because of this, scaffold DNA extending beyond the FISH signals must be subtracted from the length of the scaffold to yield the "Corrected Mb" between FISH signals that will be divided by SC length in micrometers to yield Mb/µm.

[3] = Total (corrected) Mb DNA ÷ total SC length (µm)

Table S7 Gap sizes between scaffolds determined using BAC-FISH

Chr.	Gap between Scaffold A and Scaffold B[1]	BAC Probe for Tail of Scaffold A[2]	BAC Probe for Head of Scaffold B[2]	Chromatin Type[3]	DNA Mb per µm	Gap Size SC length (µm)	Distance between BACs (Mb)	Corrected Gap Size Mb estimate[4]
1	1-3	M034D03	s053P14	Het	8.8	0.3	2.6	2.4
1	3-2	s083L21	E016I11	Cent	3.3	0.7	2.2	1.9
1	2-4	H033N15	s006A13	Het	8.8	0.1	0.9	0.6
1	4-5	s042B18	s042O02	Het	8.8	0.3	2.6	2.2
1	5-6	H037N04	s040G18	Border	5.1	0.1	0.5	0.3
1	6-8	E021C24	s022L14	Border	5.1	0.1	0.5	0.3
1	8-7	s121I01	s071P10	Eu	1.5	0.3	0.5	0.2
1	7-9	s071P10	s024J19	Eu	1.5	0.3	0.5	0.2
2	4-6	E008B15	s053E24	Het	8.8	0.1	0.7	0.4
2	6-5	s044P10	H011G24	Het	8.8	0.0	0.2	0.0
2	5-1	M036C03	s056I16	Het	8.8	0.0	0.1	0.0
2	1-2	s079J24	s053C07	Het	8.8	0.5	4.1	3.2
2	2-3	s050E09	M021A03	Het	8.8	0.1	1.0	0.5
2	3-7	E034J17	s101F18	Border	5.1	0.4	1.9	1.4
3	1-4	s086D22	s018K15	Eu	1.5	0.2	0.4	0.1
3	4-5	s002G24	s050E05	Eu	1.5	0.1	0.2	0.0
3	5-6	H037B06	H020G20	Border	5.1	0.2	1.1	0.3
3	6-9	H039C15	M079O10	Het/Cent	8.8	0.6	5.0	4.7
3	9-7	M002J15	H244B01	Het	8.8	0.1	0.6	0.2
3	7-8	E121G21	H027L13	Het	8.8	0.1	1.2	0.8
3	8-12	s071G06	E018K21	Het	8.8	0.0	0.3	0.2[5]
3	12-2	H028L03	s042B23	Het	8.8	0.0	0.3	0.0[5]
3	2-10	H030A19	H011C02	Border	5.1	0.2	0.8	0.6[5]
3	10-11	H001H05	M003H09	Eu	1.5	0.2	0.3	0.0
3	11-3	E031G05	M015G06	Eu	1.5	0.4	0.6	0.1
3	3-13	s048H23	E006F17	Eu	1.5	0.3	0.4	0.1
4	1-3	H012B20	E036C15	Het	8.8	0.06	0.5	0.3
4	3-2	H018I03	M036H17	Cent	3.3	0.2	0.6	0.4
4	2-4	M016J15	M038M16	Het	8.8	0.03	0.2	0.1
4	4-5	M029O16	H013P02	Eu	1.5	0.04	0.1	0.0
4	5-6	M018D12	H020C13	Chrom	8.8	0.2	1.9	1.6
5	1-2	H002J04	s031L05	Het	8.8	0.1	1.1	0.9
5	2-3	s084H11	H016D20	Het	8.8	0.1	1.1	0.8

6	1-3	H028K02	H025N04	Het	8.8	0.3	2.8	2.4
6	3-2	M029F23	E040C12	Het	8.8	0.1	0.8	0.6
6	2-4	E013C07	M029C07	Het	8.8	0.1	0.6	0.4
6	4-5	E009J17	E033O20	Border	5.1	0.0	0.1	0.0
6	5-6	H031J01	M034N10	Border	5.1	0.0	0.1	0.0
6	6-7	E023E10	E018M04	Eu	1.5	0.3	0.4	0.0
6	7-8	E008B16	H025F06	Eu	1.5	0.2	0.3	0.0
7	1-2	M016H01	H032J14	Het	8.8	0.06	0.5	0.4
7	2-3	E016C19	H001O15	Het	8.8	0.3	2.5	2.4
7	3-4	H024C01	E032D11	Eu	1.5	0.13	0.2	0.0
8	1-2	H008J16	H015E06	Eu	1.5	0.3	0.4	0.0
8	2-7	H009K19	M012I24	Border	5.1	0.2	0.8	0.5
8	7-5	M012I24	s093G06	Border	5.1	0.1	0.6	0.3
8	5-6	s030B23	s105O13	Border	5.1	0.04	0.2	0.0
8	6-3	M005G10	s078C24	Het	8.8	0.04	0.3	0.05
8	3-4	s058J24	E024O12	Het	8.8	0.1	1.2	1.0
8	4-8	M019D14	H033A16	Het	8.8	0.2	1.4	0.8
8	8-9	M007C12	s082B11	Eu	1.5	0.2	0.3	0.0
9	1-4	M025N23	E015A15	Het	8.8	0.2	1.7	1.5
9	4-3	E025E12	E018E08	Het	8.8	0.1	0.9	0.8
9	3-5	M005G01	M013P10	Het	8.8	0.2	1.4	1.1
9	5-6	E022O16	M018I03	Het	8.8	0.2	1.4	1.3
9	6-2	H005M15	F051C04	Eu	1.5	0.2	0.3	0.0
9	2-7	F051C04	M019G06	Eu	1.5	0.1	0.1	0.0
9	7-8	M020M04	M025N11	Eu	1.5	0.4	0.5	0.0
9	8-9	H010F04	H001I06	Eu	1.5	0.1	0.2	0.0
9	9-10	E024O21	M037I08	Eu	1.5	0.4	0.5	0.1
10	1-2	E009D07	H023E16	Border	5.1	0.02	0.1	0.0
10	2-4	s071N16	E029F05	Het	8.8	0.1	0.7	0.4
10	4-3	M011N01	s042K13	Het	8.8	0.02	0.2	0.0
10	3-5	s121P17	F014E24	Eu	1.5	0.13	0.2	0.1
10	5-6	F014E24	E036N16	Eu	1.5	0.10	0.2	0.0
11	1-4	E018D15	H008F06	Het	8.8	0.1	0.7	0.4
11	4-2	H004F15	s082L15	Het	8.8	0.1	0.9	0.6
11	2-3	H001N12	E031L09	Het	8.8	0.2	1.8	1.4
11	3-5	s045H10	s084O21	Border	5.1	0.1	0.5	0.1
11	5-6	s017L16	s105E01	Border	5.1	0.2	0.9	0.7
12	1-8	E005K14	H016B06	Border	5.1	0.1	0.4	0.2
12	8-7	M038L04	E036O23	Border	5.1	0.0	0.2	0.0

12	7-2	E003D15	M008A07	Border	5.1	0.1	0.4	0.1
12	2-6	H003H12	M023C14	Het	8.8	0.1	0.7	0.6
12	6-5	E004B02	M040D19	Het	8.8	0.1	0.8	0.5
12	5-3[6]	E012H19	H005D14	Het	8.8	0.1	1.0	0.8
12	3-4	M035N06	M006L17	Eu	1.5	0.1	0.1	0.0
12	4-9	H017P17	E031M18	Eu	1.5	0.6	0.9	0.1
12	9-10	E024H05	H029L21	Eu	1.5	0.1	0.2	0.0
							**Total:**	**43.9**

[1] Gaps are named by the two flanking scaffolds. Scaffold A is closer to the short arm of the chromosome, and across the gap, scaffold B is closer to the long arm of the chromosome. Scaffold number is based on scaffold order from head to tail of pseudomolecules based on the linkage map, and the number and name of each scaffold is defined in Table S1.

[2] BAC library names have been abbreviated. H=HindIII, E=EcoRI, M=MboI, s=sheared, F=Fosmid

[3]Eu=Euchromatin, Het=Pericentric Heterochromatin, Cent=Centromere, Chrom=Chromomere (non-pericentric heterochromatin), Border=in transition from euchromatin to heterochromatin. These landmarks are based on chromosome structure in SC spreads.

[4] Corrected gap size estimates are smaller than distance between BACs because BACs were not usually at the absolute end of the scaffold and FISH positions were calculated from the middle of the BAC rather than the end of the BAC. See Materials and Methods for procedure to correct gap size. Negative values have been changed to 0.

[5] Positions of the tail of scaffold 8, both ends of scaffolds 2 and 12, and the head of scaffold 10 are relatively uncertain, so estimates of the sizes of gaps 8-12, 12-2, and 2-10 are relatively uncertain as well.

[6] Tomato variety Heinz 1706 was used for measurements of this gap due to an inversion of the kinetochore compared to Cherry Tomato LA4444.

Table S8 Comparison of gap sizes by optical mapping and FISH

Adjacent Scaffolds		FISH Gap ID	Optical Gap Size (Kb)	FISH Gap Size (Kb)	Difference in Gap Size (FISH minus Optical) Kb[1]	Chromatin Type[3]
SL2.40SC05611	SL2.40SC05380	Chr. 12: 9-10	0	0[2]	0	Eu
SL2.40SC03622	SL2.40SC05054	Chr. 6: 7-8	1	0[2]	-1	Eu
SL2.40SC05732	SL2.40SC05054	Chr. 6: 6-7	-2	0[2]	2	Eu
SL2.40SC03798	SL2.40SC05925	Chr. 10: 1-2	2	0[2]	-2	Border
SL2.40SC05188	SL2.40SC05732	Chr. 6: 5-6	15	0[2]	-15	Het
SL2.40SC04813	SL2.40SC03770	Chr. 8: 1-2	19	0[2]	-19	Eu
SL2.40SC03835	SL2.40SC04236	Chr. 8: 6-3	23	50	27	Het
SL2.40SC03852	SL2.40SC04828	Chr. 9: 8-9	46	0[2]	-46	Eu
SL2.40SC03701	SL2.40SC06911	Chr. 3: 3-13	37	100	63	Eu
SL2.40SC04828	SL2.40SC06214	Chr. 9: 9-10	26	100	74	Eu
SL2.40SC04236	SL2.40SC03749	Chr. 8: 5-6	76	0[2]	-76	Het
SL2.40SC04915	SL2.40SC05611	Chr. 12: 4-9	16	100	84	Eu
SL2.40SC06917	SL2.40SC06903	Chr. 1: 8-7	33	200	167	Eu
SL2.40SC05941	SL2.40SC06917	Chr. 1: 6-8	68	300	232	Eu/Border
SL2.40SC03594	SL2.40SC03666	Chr. 1: 2-4	347	600	253	Het
SL2.40SC05941	SL2.40SC05010	Chr. 1: 5-6	7	300	293	Border
SL2.40SC04208	SL2.40SC04732	Chr. 2: 4-6	65	400	335	Het
SL2.40SC03731	SL2.40SC05397	Chr. 7: 1-2	48	400	352	Het
SL2.40SC06137	SL2.40SC03876	Chr. 11: 5-6	41	700	659	Eu
SL2.40SC04950	SL2.40SC04008	Chr. 9: 4-3	124	800	676	Het
SL2.40SC04757	SL2.40SC04057	Chr. 12: 5-3	42	800	758	Het
SL2.40SC04701	SL2.40SC03835	Chr. 8: 3-4	32	1000	968	Het
SL2.40SC04785	SL2.40SC04950	Chr. 9: 3-5	18	1100	1082	Het
SL2.40SC04785	SL2.40SC04777	Chr. 9: 5-6	139	1300	1161	Het
		**Totals:**	**1223**	**8250**	**7027**	

[1] The gap sizes are arranged in order of increasing absolute difference in gap size estimates. Gaps in euchromatin tend to be smaller than gaps in heterochromatin.
[2] A value of 0 kb indicates that a gap had a negative value after correcting for the position of FISH signals relative to the end of the scaffold (see Estimating Gap Sizes in Materials and Methods and in Results).
[3] Eu = euchromatin, Het = heterochromatin, Border = transition zone between euchromatin and heterochromatin

Table S9 Estimates of tomato genome size (1C DNA amount)

Method	Genome Size pg (Mb equivalent)[1]	Reference
Microspectrophotometry	1.0 (965)	(Bennett & Smith 1976)
Microspectrophotometry and flow cytometry	0.95 (917)	(Michaelson *et al.* 1991)
Flow cytometry	0.75 (724)	(Galbraith *et al.* 1983)
Flow cytometry	1.10 (1062)	(Anderson *et al.* 1985)
Flow cytometry	1.0 (965)	(Arumuganathan & Earle 1991)
Flow cytometry	0.98 (946)	(Dolezel *et al.* 1992)
Flow cytometry	0.93 (897)	(Valkonen 1994)
Flow cytometry	1.00 (965)	(Obermayer *et al.* 2002)
Cot curve	0.86 (830)	(Peterson *et al.* 1998)
**Average**	**0.95 (919)**	

[1] Picograms were converted to megabase pairs (Mb) by multiplying the number of picograms (pg) times 965 Mb (Bennett & Smith 1976).

Anderson, L. K., S. M. Stack, M. H. Fox, and C. Zhang, 1985 The relationship between genome size and synaptonemal complex length in higher plants. Exp. Cell Res. **156:** 367-378.

Arumuganathan, K., and E. D. Earle, 1991 Nuclear DNA content of some important plant species. Plant Mol. Biol. Reptr. **9:** 208-218.

Bennett, M. D., and J. B. Smith, 1976 Nuclear DNA amounts in angiosperms. Phil. Trans. Roy. Soc. Lond. B **274:** 227-274.

Dolezel, J., S. Sgorbati, and S. Lucretti, 1992 Comparison of three DNA fluorochromes for flow cytometric estimation of nuclear DNA content in plants. Physiol. Plant. **85:** 625-631.

Galbraith, D. W., K. R. Harkins, J. M. Maddox, N. M. Ayers, D. P. Sharma *et al.* 1983 Rapid flow cytometric analysis of the cell cycle in intact plant tissues. Science **220:** 1049-1051.

Michaelson, M. J., H. J. Price, J. R. Ellison, and J. S. Johnston, 1991 Comparison of plant DNA contents determined by Feulgen microspectrophotometry and laser flow cytometry. Am. J. Bot. **78:** 183-188.

Obermayer, R., I. J. Leitch, L. Hanson, and M. D. Bennett, 2002 Nuclear DNA C-values in 30 species double the familial representation in pteridophytes. Ann. Bot. **90:** 209-217.

Peterson, D. G., W. R. Pearson, and S. M. Stack, 1998 Characterization of the tomato (*Lycopersicon esculentum*) genome using in vitro and in situ DNA reassociation. Genome **41:** 346-356.

Valkonen, J. P. T., 1994 Nuclear DNA content of the Solanum spp. in the series Etuberosa as determined by laser flow cytometry. Annals of Applied Botany **125:** 589-600.

Table S10   Location of unassigned (chromosome *0*) BACs by FISH

Library	BAC ID	Chromosome	Arm	% from KC	Gap ID[1]	Flanking Scaffold	Flanking Scaffold
Mbo I	014K08	1	S	0.6%	3-2	SL2.40sc04191	SL2.40sc03666
Mbo I	056H03	2	S	8.3%	1-2	SL2.40sc06593	SL2.40sc04142
Mbo I	063O23	2	S	8.1%	1-2	SL2.40sc06593	SL2.40sc04142
Mbo I	016G20	2	S	7.7%	1-2	SL2.40sc06593	SL2.40sc04142
Mbo I	025G13	2	S	7.7%	1-2	SL2.40sc06593	SL2.40sc04142
Hind III	078N01	2	S	7.3%	1-2	SL2.40sc06593	SL2.40sc04142
EcoR I	040P16	2	S	6.8%	1-2	SL2.40sc06593	SL2.40sc04142
Hind III	009K14	2	S	6.7%	1-2	SL2.40sc06593	SL2.40sc04142
EcoR I	024C17	2	S	6.6%	1-2	SL2.40sc06593	SL2.40sc04142
Mbo I	034C01	2	S	6.2%	1-2	SL2.40sc06593	SL2.40sc04142
Mbo I	050I21	2	S	6.0%	1-2	SL2.40sc06593	SL2.40sc04142
EcoR I	031O09	2	S	5.9%	1-2	SL2.40sc06593	SL2.40sc04142
Mbo I	004J02	2	S	5.9%	1-2	SL2.40sc06593	SL2.40sc04142
Mbo I	034J07	2	S	5.9%	1-2	SL2.40sc06593	SL2.40sc04142
Hind III	058D23	2	S	5.7%	1-2	SL2.40sc06593	SL2.40sc04142
Hind III	043D06	2	S	5.6%	1-2	SL2.40sc06593	SL2.40sc04142
Hind III	012G12	2	S	5.3%	1-2	SL2.40sc06593	SL2.40sc04142
Hind III	060L16	2	S	5.3%	1-2	SL2.40sc06593	SL2.40sc04142
Mbo I	010K24	2	L	16.9%	3-7	SL2.40sc03766	SL2.40sc03665
EcoR I	032D02	2	L	17.0%	3-7	SL2.40sc03766	SL2.40sc03665
Mbo I	050I17	2	L	17.3%	3-7	SL2.40sc03766	SL2.40sc03665
EcoR I	002O15	2	L	17.3%	3-7	SL2.40sc03766	SL2.40sc03665
Hind III	060D16	2	L	17.4%	3-7	SL2.40sc03766	SL2.40sc03665
Mbo I	029F22	2	L	17.5%	3-7	SL2.40sc03766	SL2.40sc03665
Hind III	059K12	2	L	17.9%	3-7	SL2.40sc03766	SL2.40sc03665
Hind III	026M09	2	L	17.9%	3-7	SL2.40sc03766	SL2.40sc03665
EcoR I	042F18	2	L	18.1%	3-7	SL2.40sc03766	SL2.40sc03665
EcoR I	006L04	2	L	18.1%	3-7	SL2.40sc03766	SL2.40sc03665
Hind III	027N03	2	L	18.2%	3-7	SL2.40sc03766	SL2.40sc03665
EcoR I	007J10	2	L	18.3%	3-7	SL2.40sc03766	SL2.40sc03665
EcoR I	039I06	2	L	18.8%	3-7	SL2.40sc03766	SL2.40sc03665
Hind III	067G24	2	L	18.9%	3-7	SL2.40sc03766	SL2.40sc03665
EcoR I	027P01	4	L	0.3%	3-2	SL2.40sc05339	SL2.40sc03683
EcoR I	056L08	6	S	32.6%	1-3	SL2.40sc04474	SL2.40sc06140
EcoR I	047D19	6	S	31.7%	1-3	SL2.40sc04474	SL2.40sc06140

EcoR I	005K11	6	S	30.9%	1-3	SL2.40sc04474	SL2.40sc06140
EcoR I	031A01	6	S	30.8%	1-3	SL2.40sc04474	SL2.40sc06140
EcoR I	035D19	6	S	30.5%	1-3	SL2.40sc04474	SL2.40sc06140
Mbo I	049I18	6	S	29.8%	1-3	SL2.40sc04474	SL2.40sc06140
Hind III	029E01	6	S	29.6%	1-3	SL2.40sc04474	SL2.40sc06140
Hind III	027D17	6	S	28.9%	1-3	SL2.40sc04474	SL2.40sc06140
Hind III	078K13	6	S	28.7%	1-3	SL2.40sc04474	SL2.40sc06140
Hind III	002P09	6	S	28.6%	1-3	SL2.40sc04474	SL2.40sc06140
Mbo I	016K18	6	S	28.1%	1-3	SL2.40sc04474	SL2.40sc06140
EcoR I	021L01	6	S	27.6%	1-3	SL2.40sc04474	SL2.40sc06140
Hind III	001E08	6	S	27.3%	1-3	SL2.40sc04474	SL2.40sc06140
EcoR I	013G12	6	S	25.8%	1-3	SL2.40sc04474	SL2.40sc06140
EcoR I	025E06	7	S	57.1%	n/a[2]	n/a[2]	n/a[2]
Hind III	003K20	7	L	13.6%	n/a[2]	n/a[2]	n/a[2]
Hind III	027F11	7	L	19.3%	2-3	SL2.40sc05397	SL2.40sc03685
EcoR I	021M11	7	L	19.5%	2-3	SL2.40sc05397	SL2.40sc03685
EcoR I	041L20	7	L	19.9%	2-3	SL2.40sc05397	SL2.40sc03685
EcoR I	041L23	7	L	19.9%	2-3	SL2.40sc05397	SL2.40sc03685
Hind III	042K23	7	L	20.5%	2-3	SL2.40sc05397	SL2.40sc03685
Hind III	036D05	7	L	21.1%	2-3	SL2.40sc05397	SL2.40sc03685
Hind III	025A06	7	L	21.3%	2-3	SL2.40sc05397	SL2.40sc03685
Mbo I	017I04	7	L	21.5%	2-3	SL2.40sc05397	SL2.40sc03685
Mbo I	017P20	7	L	21.5%	2-3	SL2.40sc05397	SL2.40sc03685
Hind III	012C17	7	L	23.1%	2-3	SL2.40sc05397	SL2.40sc03685
EcoR I	002D11	8	S	36.5%	7-5	SL2.40sc04167	SL2.40sc03749
Hind III	041G04	8	S	35.7%	7-5	SL2.40sc04167	SL2.40sc03749
Mbo I	041D13	8	S	34.9%	7-5	SL2.40sc04167	SL2.40sc03749
EcoR I	008F12	8	L	3.1%	n/a[2]	n/a[2]	n/a[2]
Mbo I	056H18	9	S	9.4%	1-4	SL2.40sc03771	SL2.40sc04008
Hind III	072H18	9	S	6.6%	1-4	SL2.40sc03771	SL2.40sc04008
Mbo I	045G03	9	S	3.4%	1-4	SL2.40sc03771	SL2.40sc04008
EcoR I	052D12	9	S	3.4%	1-4	SL2.40sc03771	SL2.40sc04008
Mbo I	042F10	9	L	99.6%	10-T	SL2.40sc06214	telomere
EcoR I	045G19	11	L	8.0%	n/a[2]	n/a[2]	n/a[2]
Mbo I	047F15	11	L	14.3%	2-3	SL2.40sc04054	SL2.40sc03752
Hind III	062E02	11	L	15.2%	2-3	SL2.40sc04054	SL2.40sc03752
Mbo I	050G12	12	S	36.9%	1-8	SL2.40sc04607	SL2.40sc06147
EcoR I	010P04	12	S	35.8%	8-7	SL2.40sc06147	SL2.40sc04039
Hind III	006G23	12	L	23.6%	n/a[2]	n/a[2]	n/a[2]

EcoR I	045D23	12	L	81.0%	9-10	SL2.40sc05611	SL2.40sc05380

[1]Gap ID refers to the gap name given in the FISH Scaffold/Gap diagrams and is derived from the linkage map-based order of the scaffolds.

[2] BAC is not within a gap

Table S11 List of all of unassigned (chromosome 0) BACs.

Available for download as an Excel file at http://www.g3journal.org/lookup/suppl/doi:10.1534/g3.114.011197/-/DC1
