# Supplementary material for: Fluorescence In Situ Hybridization and Optical Mapping to Correct Scaffold Arrangement in the Tomato Genome
Source: G3 (Bethesda). 2014 May 30;4(8):1395–405. doi: 10.1534/g3.114.011197 (PMC4132171; doi:10.1534/g3.114.011197)
Supplement: Supporting Information [file supp_g3.114.011197_FileS1.pdf]

File S1

Materials and Methods

Isolating BACs. The following modifications were made in the standard protocol for plasmid isolation using an AquaPlasmid kit (MultiTarget Pharmaceuticals, Salt Lake City, Utah). Bacterial culture volume was increased from 2 ml to 4 ml. After removing supernatant from the bacterial pellet, bacteria were suspended in 100 µL of deionized water containing 400 µg/ml ribonuclease A instead of 80 µl of deionized water. After addition of AquaLysis, instead of mixing by vortexing, the tubes were rocked gently for 5 minutes. 50 µL of 3M sodium acetate was added to each tube and tubes were rocked gently for 5 minutes to mix. Then AquaPlasmid reagents were added and mixed gently by rocking for 5 minutes. Centrifugation was increased from 5 minutes to 20 minutes to pellet debris after the addition of AquaLysis.

Determining the amount of DNA per micrometer of SC (= linear DNA density) in kinetochores, euchromatin, and heterochromatin. The lengths of scaffolds that are wholly contained in a kinetochore, or in distal euchromatin, or in pericentric heterochromatin were measured as a percent of arm length between the fluorescent signal of a BAC at the head of the scaffold and the fluorescent signal of a BAC at the tail of the scaffold. These percent lengths were converted to micrometers based on the average pachytene SC set (see *Measuring positions of BACs on SCs* in Materials and Methods). Before linear density of DNA can be calculated, a correction must be made to take into account the fact that amount of DNA (scaffold size) between FISH foci is actually an overestimate because 1) a FISH focus is considered to be in the center of a BAC and 2) sometimes subterminal BACs rather than terminal BACs in scaffolds were used for FISH in cases where terminal BACs did not work for FISH. Therefore, there is less DNA between the FISH signals than in the scaffold because some scaffold DNA extends beyond the FISH signals into the adjacent gaps. As a correction, the scaffold DNA extending beyond the FISH signals was subtracted from the scaffold size to yield the amount of DNA between the FISH foci. For example, based on BAC-FISH, scaffold SL2.40sc04626 in the euchromatin of the long arm of SC *7* is measured to be 2.0 µm long. This scaffold is known to be is 3.39 Mb long (http://solgenomics.net). On the left side of the scaffold, the localized BAC SL_EcoRI0032D11 is 150 kb long. Assuming the fluorescent signal is in the middle of the BAC, (150 kb ÷ 2 =) 75 kb that extends beyond the FISH signal was subtracted from the scaffold size. Because this BAC starts 11 kb into the scaffold, another 11 kb was subtracted from the scaffold size. On the right side of the scaffold the localized BAC SL_MboI0017L19 is 74 Mb long, so an additional (74 Mb ÷ 2 =) 37 kb was subtracted from the scaffold size. Also, this BAC is not terminal, but starts 99 kb from the right end of the scaffold, so an additional 99 kb was subtracted from the scaffold size. Thus, the corrected amount of DNA between these two FISH signals is (3390 kb – 11 kb – 75 kb – 37 kb – 99 kb =) 3168 kb or ~3.2 Mb compared to the complete scaffold size of 3.39 Mb.

Adjusting measured gap sizes. Gap lengths were initially determined by measuring the distance between the fluorescent focus of a BAC at or near the end of one scaffold and the fluorescent focus of another BAC at or near the end of the adjacent scaffold. This measurement, expressed as a percent of the arm length, was repeated on ten or more SC spreads to obtain an average distance expressed as a percent of the arm length between the two BACs. This percent distance was converted to micrometers by multiplying the percent (decimal fraction) times the average length of this SC arm in micrometers that was determined by Sherman and Stack (1992). This distance in micrometers was converted to a quantity of DNA based on the linear (DNA) density of the chromatin type where the gap is located (Table S6). Gap sizes calculated this way are overestimates because 1) FISH foci are assumed to be in the center of a localized BAC and 2) subterminal BACs rather than terminal BACs in scaffolds often were used for FISH. Therefore, the measured gap sizes should be reduced by the amount of DNA estimated to extend from the FISH signals into the gap. For example, gap 9-10 on the long arm of SC 12, *i.e*., the gap between scaffolds SL2.40sc05611and SL2.40sc05380, was measured to be 0.12 μm long in euchromatin and the amount of DNA in the gap is estimated to be (1.5 Mb/μm X 0.12 μm =) 0.18 Mb or 180 kb. On the left side of the gap, the localized BAC SL_EcoRI0024H05 is 106 kb long, and this BAC is the terminal (the last) BAC in the scaffold. Assuming that the fluorescent focus is in the middle of the BAC (106 kb ÷ 2 =) 53 kb should be subtracted from the gap size. On the right side of the scaffold the localized BAC Le_HBa0029L21 is 120 kb long, so an additional (120kb ÷ 2=) 60 kb should be subtracted from the scaffold size. Also the BAC localized to the right is not the terminal BAC, but 53 kb from the end of the right scaffold, so this amount of DNA also should be subtracted from the gap size. Thus, the corrected gap size is (180 kb – 53 kb – 60 kb – 53 kb =) 14 kb.

REFERENCE

Sherman, J. D. and S. M. Stack, 1992 Two-dimensional spreads of synaptonemal complexes from solanaceous plants. V. Tomato (*Lycopersicon esculentum*) karyotype and idiogram. Genome **35**: 354-359.
