# Supplementary material for: Fluorescence In Situ Hybridization and Optical Mapping to Correct Scaffold Arrangement in the Tomato Genome
Source: G3 (Bethesda). 2014 May 30;4(8):1395–405. doi: 10.1534/g3.114.011197 (PMC4132171; doi:10.1534/g3.114.011197)
Supplement: Supporting Information [file supp_g3.114.011197_FigureS1.pdf]

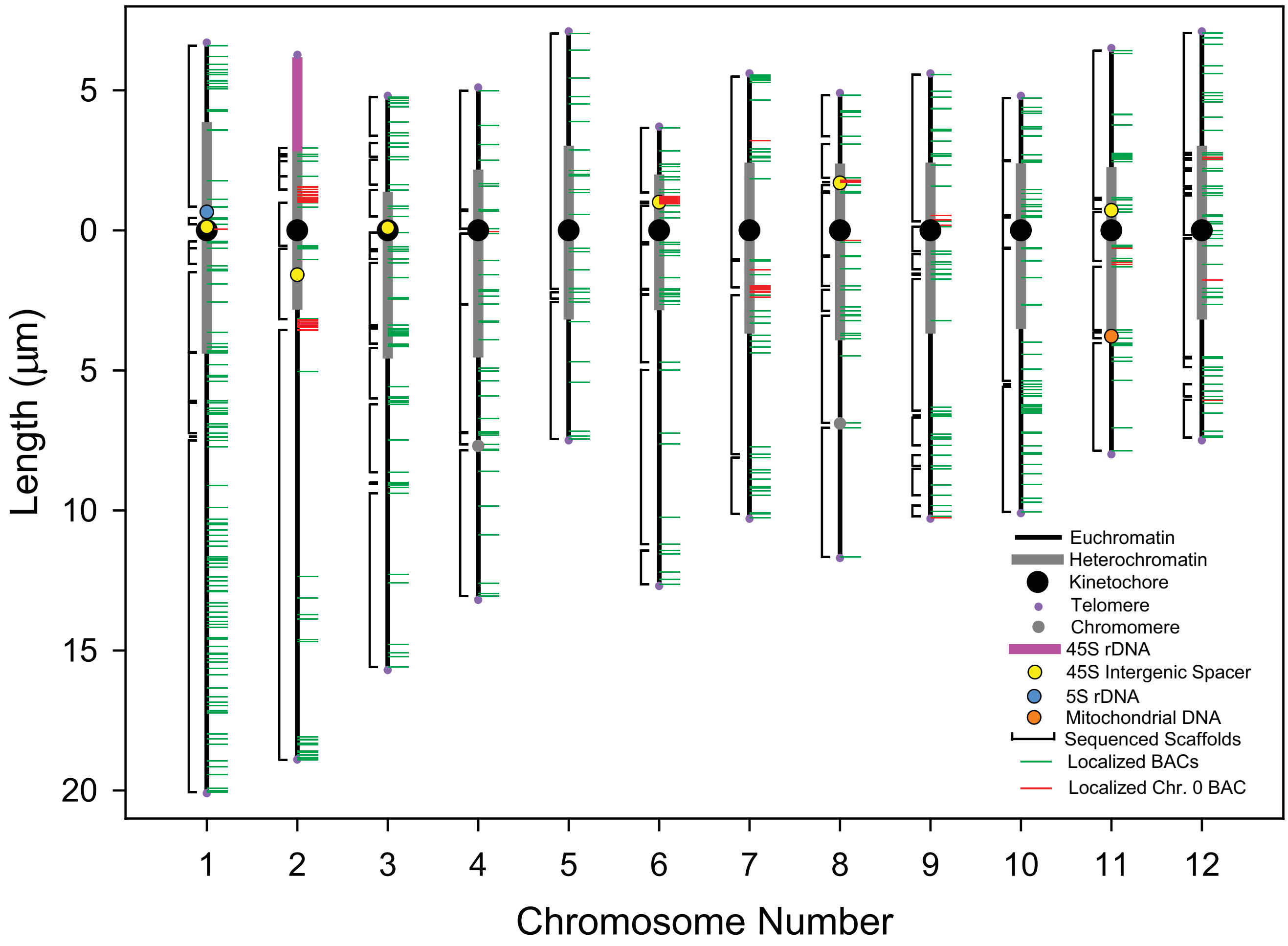

Figure S1 Diagrammatic representation of BAC- and repeated sequence-FISH localizations relative to inferred scaffold positions on an idiogram of the twelve tomato SCs. The twelve vertically oriented tomato SCs are numbered along the bottom. For each SC, black lines represent SC in distal euchromatin, thick grey lines represent SC in pericentric heterochromatin, and kinetochores (centromeres) are shown as black disks aligned at 0 µm on the left scale. Grey dots in the euchromatin of the long arms of SCs *4* and *8* represent chromomeres. The short arm of chromosome *2* is entirely heterochromatic with the nucleolus organizer region (NOR) in the thick, pink, terminal segment marking the location of 45S rDNA. Other repeated sequences localized by FISH include sites of 45S rDNA intergenic spacer sequence (yellow spots on SCs *1*, *2*, *3*, *6*, *8* and *11)*, 5S rDNA (blue spot in the short arm of SC *1*), a concentration of mitochondrial DNA (orange spot on SC *11*), and canonical $(TTTAGGG)_n$ telomere repeats (purple knobs at the ends of all chromosomes). Short horizontal red and green lines extending to the right of each SC show BACs located by FISH. Red lines indicate the locations of 75 chromosome *0* BACs, while green lines indicate the locations of all other BACs. A total of 627 BACs have been localized, but there are 639 horizontal lines because twelve of the BACs hybridize to two locations (Figure S1). See http://solgenomics.net/cview/map.pl?map_version_id=25 for identification of each BAC on the idiogram and the photographic data for every FISH localization. Brackets to the left of each SC mark chromosomal locations of scaffolds, while spaces between brackets represent gaps between scaffolds. Some scaffolds are so small that they appear only as lines in gaps, and some gaps are so small that no space is visible between adjacent brackets. Note that many chromosome *0* BACs (red horizontal lines) occur in gaps and that most of the locations of repeated sequences also correspond to gaps between sequenced scaffolds.
