# Supplementary material for: Fluorescence In Situ Hybridization and Optical Mapping to Correct Scaffold Arrangement in the Tomato Genome
Source: G3 (Bethesda). 2014 May 30;4(8):1395–405. doi: 10.1534/g3.114.011197 (PMC4132171; doi:10.1534/g3.114.011197)
Supplement: Supporting Information [file supp_g3.114.011197_FigureS2.pdf]

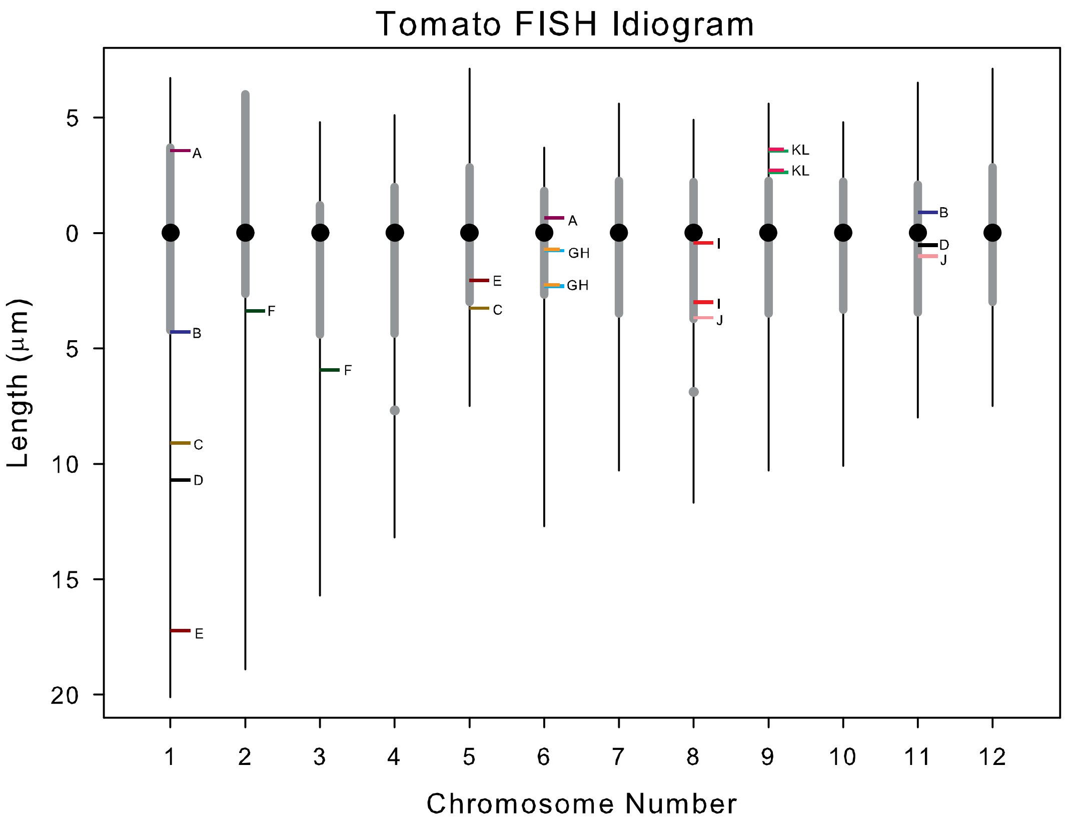

Figure S2 Idiogram of tomato SCs with FISH localizations of BACs having two hybridization sites. Each BAC is represented by two horizontal bars of the same color. Five BACs hybridize to two locations on the same SC. Two of these BACs are close to each other on SC *6* (G and H), two are close to each other on SC *9* (K and L), and one is on SC *8* (I). On SCs *6* and *9*, the two BACs are so close together that they must carry DNA sequence from the same small duplicated chromosome segment. The other seven BACs each hybridize at one location on two different SCs, A on *1* and *6*, B on *1* and *11*, C on *1* and *5*, D on *1* and *11*, E on *1* and *5*, F on *2* and *3*, J on *8* and *11*. These apparent duplications also could be due to chimeric BACs (one BAC with two random segments of tomato DNA), and/or cultures with two BACs, *i.e.*, contaminated cultures. Key to duplications: A is SL_MboI0081L19, B is LE_HBa0023H04, C is LE_HBa0006D05, D is LE_HBa0305L18, E is LE_HBa0033M02, F is LE_HBa0101C24, G is LE_HBa0031J01, H is SL_MboI0034N14, I is SL_MboI0012P13, J is SL_EcoRI0027A06, K is LE_HBa0300E15 and L is LE_HBa0026P14.
