# Supplementary material for: Fluorescence In Situ Hybridization and Optical Mapping to Correct Scaffold Arrangement in the Tomato Genome
Source: G3 (Bethesda). 2014 May 30;4(8):1395–405. doi: 10.1534/g3.114.011197 (PMC4132171; doi:10.1534/g3.114.011197)
Supplement: Supporting Information [file supp_g3.114.011197_FigureS3.pdf]

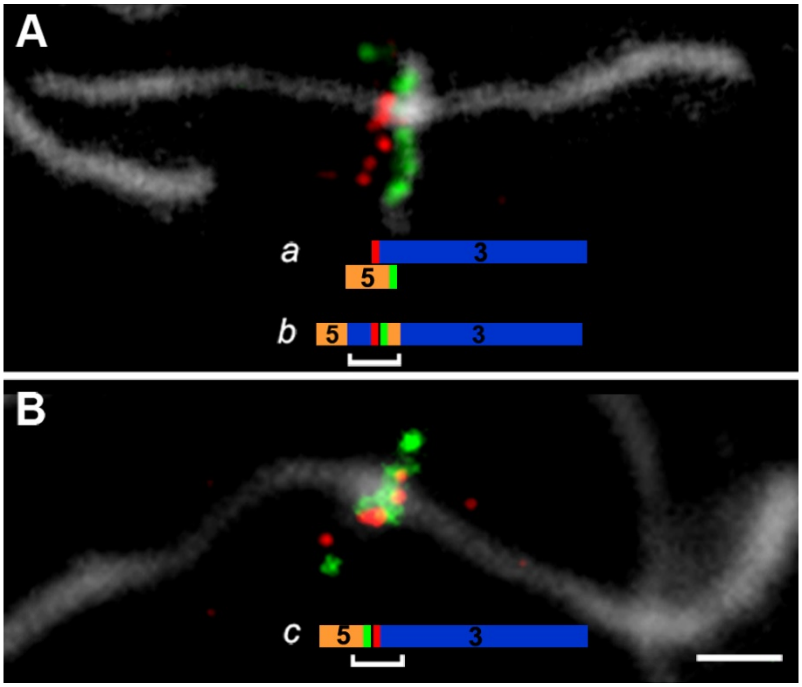

Figure S3 FISH reveals a probable inversion around the centromere of chromosome *12*, which distinguishes tomato var. *Cherry*, accession LA4444 and *S. lycopersicum*, var. Heinz 1706. Digitally reversed phase images of SC *12* from (**A**) LA4444 and (**B**) Heinz 1706 with overlaid FISH signals for probes LE_HBa0005D14 (red) that is part of scaffold SL2.40sc04057 (= scaffold 3) and SL_EcoRI0012H19 (green) that is part of scaffold SL2.40sc04757 (= scaffold 5). Both SCs are oriented with the short arms to the left. Both probes hybridize near the centromere in both lines, but the order of the two probes relative to the short arm differs. Although not shown here, probes that mark the opposite ends of scaffolds 3 and 5 are located in the same relative positions in the long and short arms in both lines. Interpretive drawings (a-*c*) of scaffolds 3 (blue) and 5 (orange) with the red bar indicating the approximate position of probe LE_HBa0005D14 on scaffold 3 and the green bar indicating the approximate position of probe SL_EcoRI0012H19 on scaffold 5. (***a***) The positions of the probes on these two scaffolds indicate that the scaffolds overlap in LA4444. However, sequencing of Heinz 1706 did not reveal any such overlap. (***b***) Diagram of a probable inversion (shown by the bracket underneath) of a segment of chromosome *12* from LA4444 relative to Heinz 1706, which explains the observed probe locations. (***c***) The positions of the probes at the ends of scaffolds 5 and 3 on chromosome *12* of Heinz 1706 are consistent with two adjacent scaffolds. The bracket underneath indicates the location of the chromosome segment inverted between LA4444 and Heinz 1706. Bar equals 2 µm for **A** and **B**. The drawings (***a-c***) are not to scale except the lengths of the two scaffolds are correct relative to each other.
