# Supplementary material for: Fluorescence In Situ Hybridization and Optical Mapping to Correct Scaffold Arrangement in the Tomato Genome
Source: G3 (Bethesda). 2014 May 30;4(8):1395–405. doi: 10.1534/g3.114.011197 (PMC4132171; doi:10.1534/g3.114.011197)
Supplement: Supporting Information [file supp_g3.114.011197_FigureS4.pdf]

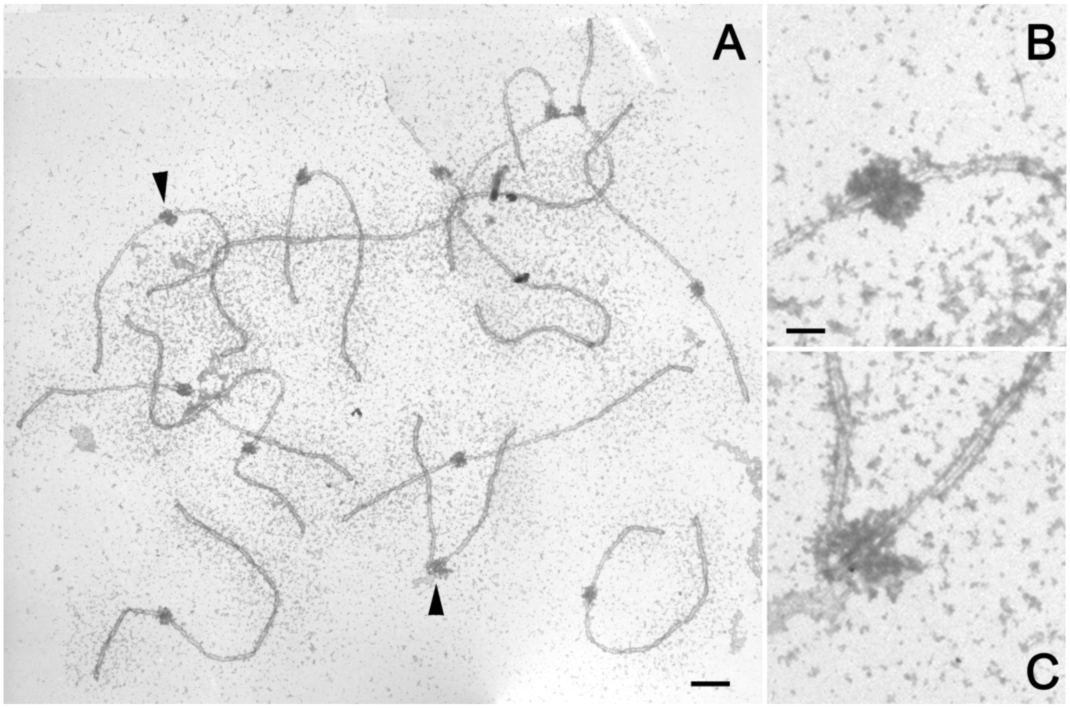

Figure S4 Electron micrographs of phosphotungstic acid (PTA)-stained SCs from a Heinz 1706 X Cherry LA4444 tomato hybrid. **A**. Complete SC spread showing no synaptic irregularities that would indicate structural heterozygosities between the two lines. Chromosomes *5* and *12* (indicated by arrowheads at kinetochores) are structurally indistinguishable. **B**. and **C.** Higher magnification views of pericentric regions of SCs *5* and *12* from (**A**). Both chromosomes show straight synapsis through the kinetochore, even though the hybrid is probably heterozygous for an inversion involving the kinetochore of chromosome *12*. SC spreading and PTA staining was performed as described by Stack and Anderson (2009). Bar in A = 2 µm, bar in B (and C) = 0.5 µm
