# Supplementary material for: Fluorescence In Situ Hybridization and Optical Mapping to Correct Scaffold Arrangement in the Tomato Genome
Source: G3 (Bethesda). 2014 May 30;4(8):1395–405. doi: 10.1534/g3.114.011197 (PMC4132171; doi:10.1534/g3.114.011197)
Supplement: Supporting Information [file supp_g3.114.011197_TableS2.pdf]

Table S2 Tomato SC karyotype[1]

Chromosome number	% of set	Chromosome length (µm)	Arm ratio	Short arm length (µm)	Long arm length (µm)
1	13.1	26.8	3.00	6.7	20.1
2	8.5	24.9	3.15	6.0	18.9
3	9.8	20.5	3.27	4.8	15.7
4	8.7	18.3	2.59	5.1	13.2
5	7.2	14.6	1.06	7.1	7.5
6	7.9	16.4	3.43	3.7	12.7
7	7.8	15.9	1.80	5.6	10.3
8	7.9	16.6	2.39	4.9	11.7
9	7.8	15.9	1.83	5.6	10.3
10	7.2	14.9	2.10	4.8	10.1
11	6.9	14.5	1.23	6.5	8.0
12	7.2	14.6	1.06	7.1	7.5
**Total**	**100.0**	**213.9**		**67.9**	**146.0**

[1]Modified from Sherman and Stack (1992) and Peterson et al. (1995)
