# Supplementary material for: Fluorescence In Situ Hybridization and Optical Mapping to Correct Scaffold Arrangement in the Tomato Genome
Source: G3 (Bethesda). 2014 May 30;4(8):1395–405. doi: 10.1534/g3.114.011197 (PMC4132171; doi:10.1534/g3.114.011197)
Supplement: Supporting Information [file supp_g3.114.011197_TableS3.pdf]

Table S3 All BACs and repeated sequences located by FISH on tomato SCs. Unless otherwise noted, all BACs are from the HindIII BAC library.

Chromosome arm	BAC	Position %	Position um (SC)	Scaffold	Legend Key
	Telomere	100.0%	0.0		**S = short arm; L = long arm**
**1S**	**SL_s0090M22**	**98.5%**	**0.1**	**S1**	Telomere
1S	165H20	92.6%	0.5		Eu/Het border using RNs to adjust estimate based on SC appearance
1S	088A08	88.5%	0.8		Centromere
1S	130I12	85.7%	1.0		Less than 1 micron from a telomere
1S	069E17	84.1%	1.1		or eu/het border
1S	240P19	83.0%	1.1		Less than 1 micron from a centromere
1S	262O22	79.7%	1.4		**End of a scaffold**
1S	305J13	78.3%	1.5		BAC with 2 positions
1S	291L11	76.4%	1.6		Repeated sequences
1S	263P17	75.6%	1.6		IGS = inter-genic spacer repeat from rDNA
1S	032H01	64.3%	2.4		
1S	003D15	63.5%	2.4		
1S	Eu/Het border	55.0%	3.0		
1S	SL_MboI0012I20	53.5%	3.1		

1S	SL_MboI0081L19	53.2%	3.1	
1S	177F14	26.4%	4.9	
1S	107C12	16.5%	5.6	
**1S**	**SL_MboI0034D03**	**12.5%**	**5.9**	**S1**
1S	027P13	12.4%	5.9	
1S	5S rDNA	9.8%	6.0	
**1S**	**SL_s0053P14**	**6.6%**	**6.3**	**S3**
1S	081I16	5.8%	6.3	
**1S**	**SL_s0083L21**	**3.2%**	**6.5**	**S3**
1S	IGS-derived satellite	1.8%	6.6	
1S	SL_MboI0014K08	0.6%	**6.7**	
	Centromere	0.0%	6.7	
**1L**	**SL_EcoRI0016I11**	**2.0%**	**7.1**	**S2**
1L	054N01	2.2%	7.2	
**1L**	**SL_s0006A13**	**3.2%**	**7.3**	**S2**
**1L**	**033N15**	**3.2%**	**7.3**	**S4**
1L	256E08	6.3%	8.0	
**1L**	**SL_s0042B18**	**6.9%**	**8.1**	**S4**
**1L**	**SL_MboI0042O02**	**7.2%**	**8.1**	**S5**
1L	061J17	9.5%	8.6	
1L	252G05	12.8%	9.3	
1L	017P20	18.1%	10.3	
1L	174H02	20.1%	10.7	
1L	126F09	20.7%	10.9	

1L	048O19	20.8%	10.9	
1L	Eu/Het border	21.0%	10.9	
1L	023H04	21.3%	11.0	
1L	163B20	21.3%	11.0	
**1L**	**037N04**	**21.6%**	**11.0**	**S5**
**1L**	**SL_s0040G18**	**21.8%**	**11.1**	**S6**
1L	095K03	23.9%	11.5	
1L	033C15	25.7%	11.9	
1L	305F14	26.0%	11.9	
1L	087D19	26.8%	12.1	
**1L**	**SL_EcoRI0021C24**	**30.3%**	**12.8**	**S6**
**1L**	**SL_s0022L14**	**30.6%**	**12.9**	**S8**
1L	004L01	30.6%	12.9	
1L	210F09	31.6%	13.1	
1L	SL_MboI0017A22	32.0%	13.1	
1L	SL_MboI0008G17	32.0%	13.1	
1L	SL_MboI0034B03	32.4%	13.2	
1L	SL_EcoRI0021A20	32.6%	13.3	
1L	SL_EcoRI0022O19	34.4%	13.6	
1L	055E23	34.8%	13.7	
1L	001J06	35.0%	13.7	
**1L**	**SL_s0121I01**	**36.0%**	**13.9**	**S8**
**1L**	**SL_s0071P10**	**36.6%**	**14.1**	**S7**
1L	067M04	36.8%	14.1	

1L	039M19	36.8%	14.1	
**1L**	**SL_s0024J19**	**37.3%**	**14.2**	**S9**
1L	029G15	37.3%	14.2	
1L	155M04	38.4%	14.4	
1L	006D05	45.3%	15.8	
1L	329A12	49.2%	16.6	
1L	289N16	51.3%	17.0	
1L	035M19	52.0%	17.2	
1L	182E16	52.3%	17.2	
1L	053C22	52.3%	17.2	
1L	305L18	53.2%	17.4	
1L	208M24	54.2%	17.6	
1L	034P21	55.2%	17.8	
1L	049F03	56.1%	18.0	
1L	330O05	58.0%	18.4	
1L	108J06	58.4%	18.4	
1L	140O23	58.6%	18.5	
1L	234D05	59.1%	18.6	
1L	080J18	59.8%	18.7	
1L	309D12	61.5%	19.1	
1L	125A09	62.3%	19.2	
1L	203E06	63.1%	19.4	
1L	123A07	64.0%	19.6	
1L	169H23	64.0%	19.6	

1L	058O13	64.2%	19.6
1L	057C07	66.2%	20.0
1L	159C14	66.8%	20.1
1L	165M11	66.8%	20.1
1L	051C14	67.8%	20.3
1L	010F03	68.7%	20.5
1L	005L21	69.5%	20.7
1L	001H15	70.0%	20.8
1L	302G11	70.5%	20.9
1L	057A01	72.3%	21.2
1L	SL_EcoRI0127B04	72.6%	21.3
1L	111D21	73.9%	21.6
1L	024O06	75.1%	21.8
1L	043D23	75.3%	21.8
1L	164M23	76.1%	22.0
1L	243A15	76.7%	22.1
1L	026N10	77.8%	22.3
1L	057J16	78.9%	22.6
1L	049P09	81.3%	23.0
1L	037D10	82.9%	23.4
1L	245N21	83.8%	23.5
1L	002F21	83.8%	23.5
1L	100N21	84.4%	23.7
1L	079N04	85.4%	23.9

1L	092H13	85.4%	23.9	
1L	033M02	85.7%	23.9	
1L	239E07	89.5%	24.7	
1L	064D24	90.3%	24.9	
1L	008L19	91.3%	25.0	
1L	131F15	94.2%	25.6	
1L	128J14	95.2%	25.8	
1L	043P11	96.7%	26.1	
1L	231M15	99.1%	26.6	
1L	088L02	99.5%	26.7	
**1L**	**SL_MboI0028C09**	**99.8%**	**26.8**	**S9**
1L	Telomere	100.0%	26.8	
2S	Telo./Break Point*	100.0%	0.0	
2S	45S rDNA	16.0%	3.0	
**2S**	**SL_EcoRI0007F19**	**15.6%**	**3.1**	**S4**
**2S**	**SL_EcoRI0008B15**	**14.4%**	**3.3**	**S4**
**2S**	**SL_s0053E24**	**14.0%**	**3.4**	**S6**
**2S**	**SL_s0044P10**	**13.1%**	**3.5**	**S6**
**2S**	**LE_HBa0011G24**	**13.1%**	**3.5**	**S5**
**2S**	**SL_s0056I16**	**10.2%**	**4.1**	**S5**
**2S**	**SL_MboI0036C03**	**10.2%**	**4.1**	**S1**
2S	SL_MboI0056H03	8.3%	4.4	
2S	SL_MboI0063O23	8.1%	4.5	

**2S**	**SL_s0079J24**	**7.7%**	**4.5**	**S1**
2S	SL_MboI0025G13	7.7%	4.5	
2S	SL_MboI0016G20	7.7%	4.5	
2S	078N01	7.3%	4.6	
2S	SL_EcoRI0040P16	6.8%	4.7	
2S	009K14	6.7%	4.7	
2S	111L05	6.7%	4.7	
2S	SL_EcoRI0024C17	6.6%	4.8	
2S	SL_MboI0034C01	6.2%	4.8	
2S	SL_MboI0050I21	6.0%	4.9	
2S	SL_MboI0034J07	5.9%	4.9	
2S	SL_EcoRI0031O09	5.9%	4.9	
2S	SL_MboI0004J02	5.9%	4.9	
2S	058D23	5.7%	4.9	
2S	043D06	5.6%	4.9	
2S	060L16	5.3%	5.0	
2S	012G12	5.3%	5.0	
**2S**	**SL_s0053C07**	**5.2%**	**5.0**	**S2**
2S	090J13	4.4%	5.2	
	Centromere	0.0%	6.0	
**2L**	**SL_s0050E09**	**2.9%**	**6.5**	**S2**
2L	006P20	3.2%	6.6	
**2L**	**SL_MboI0021A03**	**3.5%**	**6.7**	**S3**
2L	060G11	5.5%	7.0	

2L	IGS-derived satellite	8.4%	7.6	
2L	Eu/Het border	14.0%	8.6	
2L	176A14	16.7%	9.2	
**2L**	**SL_EcoRI0034J17**	**16.8%**	**9.2**	**S3**
2L	SL_MboI0010K24	16.9%	9.2	
2L	SL_EcoRI0032D02	17.0%	9.2	
2L	SL_MboI0050I17	17.3%	9.3	
2L	SL_EcoRI0002O15	17.3%	9.3	
2L	060D16	17.4%	9.3	
2L	SL_MboI0029F22	17.5%	9.3	
2L	101C24	17.9%	9.4	
2L	026M09	17.9%	9.4	
2L	059K12	17.9%	9.4	
2L	SL_EcoRI0006L04	18.1%	9.4	
2L	SL_EcoRI0042F18	18.1%	9.4	
2L	027N03	18.2%	9.4	
2L	SL_EcoRI0007J10	18.3%	9.5	
2L	SL_EcoRI0039I06	18.8%	9.6	
**2L**	**SL_s0101F18**	**18.8%**	**9.6**	**S7**
2L	067G24	18.9%	9.6	
2L	303I24	26.7%	11.0	
2L	286I11	65.4%	18.4	
2L	108A18	69.4%	19.1	
2L	106H06	72.6%	19.7	

2L	118M12	73.4%	19.9	
2L	001M12	77.3%	20.6	
2L	189G15	77.7%	20.7	
2L	SL_MboI0055O24	95.7%	24.1	
2L	064B17	96.2%	24.2	
2L	SL_EcoRI0034H10	96.3%	24.2	
2L	072B02	96.9%	24.3	
2L	SL_EcoRI0042D07	97.2%	24.4	
2L	SL_EcoRI0061K08	98.4%	24.6	
2L	257H21	98.7%	24.7	
2L	098J01	99.1%	24.7	
2L	032J10	99.6%	24.8	
2L	177F12	99.8%	24.9	
**2L**	**SL_MboI0017J13**	**100.0%**	**24.9**	**S7**
2L	Telomere	100.0%	24.9	
3S	Telomere	100.0%	0.0	
**3S**	**SL_s0009C01**	**99.3%**	**0.0**	**S1**
3S	020P05	98.0%	0.1	
3S	SL_MboI0103M17	96.6%	0.2	
3S	203C09	94.8%	0.3	
3S	SL_FOS0082H20	92.4%	0.4	
3S	SL_FOS0097P22	92.1%	0.4	
3S	137K15	80.6%	0.9	
3S	257N18	72.6%	1.3	

**3S**	**SL_s0086D22**	**70.2%**	**1.4**	**S1**
**3S**	**SL_s0018K15**	**65.1%**	**1.7**	**S4**
3S	030A11	65.0%	1.7	
3S	031M05	62.1%	1.8	
**3S**	**SL_s0002G24**	**54.9%**	**2.2**	**S4**
**3S**	**SL_s0050E05**	**52.5%**	**2.3**	**S5**
**3S**	**037B06**	**34.2%**	**3.2**	**S5**
**3S**	**020G20**	**29.8%**	**3.4**	**S6**
3S	Eu/Het border	25.0%	3.6	
3S	130G19	17.9%	3.9	
3S	162G22	16.2%	4.0	
**3S**	**039C15**	**10.2%**	4.3	**S6**
3S	IGS-derived satellite	1.9%	4.7	
	Centromere	0.0%	4.8	
**3L**	**SL_MboI0079O10**	**0.5%**	4.9	**S9**
3L	007J09	3.7%	5.4	
**3L**	**SL_MboI0002J15**	**4.3%**	5.5	**S9**
**3L**	**244B01**	**4.7%**	5.5	**S7**
**3L**	**SL_EcoRI0121G21**	**6.5%**	5.8	**S7**
**3L**	**027L13**	**7.4%**	6.0	**S8**
3L	197O22	10.7%	6.5	
3L	014A17	15.3%	7.2	
3L	077H15	15.6%	7.2	
**3L**	**SL_s0071G06**	**21.5%**	**8.2**	**S8**

**3L**	**SL_s0042B23**	**22.3%**	**8.3**	**S2**
3L	SL_MboI0022A20	22.4%	8.3	
3L	SL_MboI0018H18	22.6%	8.3	
3L	157B13	23.1%	8.4	
3L	011P01	23.2%	8.4	
3L	SL_MboI0023D14	23.2%	8.4	
3L	084A03	23.4%	8.5	
**3L**	**SL_EcoRI0018K21**	**23.4%**	**8.5**	**S12**
**3L**	**028L03**	**23.7%**	**8.5**	**S12**
3L	SL_MboI0011B02	24.0%	8.6	
3L	011C02	25.9%	8.9	
**3L**	**037N01**	**26.1%**	**8.9**	**S10**
3L	SL_MboI0015M02	26.2%	8.9	
**3L**	**030A19**	**26.3%**	**8.9**	**S2**
3L	299H10	26.5%	9.0	
3L	Eu/Het border	28.0%	9.2	
3L	031P17	35.5%	10.4	
3L	101C24	37.9%	10.7	
**3L**	**001H05**	**38.2%**	**10.8**	**S10**
3L	028E17	38.8%	10.9	
3L	254B14	39.1%	10.9	
**3L**	**SL_MboI0003H09**	**39.6%**	**11.0**	**S11**
3L	SL_s0003D15	47.7%	12.3	
**3L**	**SL_EcoRI0031G05**	**55.0%**	**13.4**	**S11**

**3L**	**SL_MboI0015G06**	**57.4%**	**13.8**	**S3**
**3L**	**SL_s0048H23**	**57.7%**	**13.9**	**S3**
3L	SL_EcoRI0034O23	57.9%	13.9	
3L	117I12	58.4%	14.0	
**3L**	**SL_EcoRI0006F17**	**59.8%**	**14.2**	**S13**
3L	157P11	78.3%	17.1	
3L	276M05	80.2%	17.4	
3L	241F16	94.2%	19.6	
3L	142A05	96.0%	19.9	
3L	159C06	97.0%	20.0	
**3L**	**SL_EcoRI0034G21**	**99.3%**	**20.4**	**S13**
3L	Telomere	100.0%	20.5	
4S	Telomere	100.0%	0.0	
**4S**	**SL_MboI0035I14**	**97.9%**	**0.1**	**S1**
4S	008K08	73.6%	1.3	
4S	036C23	60.3%	2.0	
4S	323C04	49.1%	2.6	
4S	Eu/Het border	39.0%	3.1	
4S	062J03	32.6%	3.4	
4S	209A01	30.8%	3.5	
**4S**	**012B20**	**14.7%**	**4.4**	**S1**
**4S**	**SL_EcoRI0036D15**	**13.6%**	**4.4**	**S3**
**4S**	**018I03**	**1.0%**	**5.0**	**S3**
	Centromere	0.0%	5.1	

4L	SL_EcoRI0027P01	0.3%	5.1	
**4L**	**SL_MboI0036H17**	**0.9%**	**5.2**	**S2**
4L	059C20	8.2%	6.2	
4L	105J24	12.0%	6.7	
4L	030F21	16.1%	7.2	
4L	007D04	16.5%	7.3	
4L	203K18	17.8%	7.4	
**4L**	**SL_MboI0016J15**	**19.9%**	**7.7**	**S2**
**4L**	**SL_MboI0038M16**	**20.1%**	**7.8**	**S4**
4L	006E18	24.5%	8.3	
4L	SL_MboI0078A08	24.8%	8.4	
4L	SL_MboI0120F05	29.6%	9.0	
4L	Eu/Het border	33.0%	9.5	
4L	291H22	37.3%	10.0	
4L	119A16	38.0%	10.1	
4L	020F17	40.7%	10.5	
4L	077O05	44.8%	11.0	
4L	078E04	50.9%	11.8	
4L	208L16	54.4%	12.3	
**4L**	**SL_MboI0029O16**	**54.6%**	**12.3**	**S4**
**4L**	**013P02**	**54.9%**	**12.3**	**S5**
4L	132O11	54.9%	12.3	
4L	171B19	55.5%	12.4	
**4L**	**SL_MboI0018D12**	**57.9%**	**12.7**	**S5**

4L	Chromomere	58.1%	12.8	
**4L**	**020C13**	**59.5%**	**13.0**	**S6**
4L	094K06	59.2%	12.9	
4L	SL_MboI0039E17	65.2%	13.7	
4L	053M02	74.6%	14.9	
4L	008K13	82.3%	16.0	
4L	303A06	95.4%	17.7	
4L	106F07	98.2%	18.1	
**4L**	**SL_MboI0023M21**	**98.9%**	**18.2**	**S6**
4L	Telomere	100.0%	18.3	
5S	Telomere	100.0%	0.0	
**5S**	**SL_s0094J05**	**98.9%**	**0.1**	**S1**
5S	189E17	90.6%	0.7	
5S	309L13	76.6%	1.7	
5S	147F10	67.4%	2.3	
5S	157F14	63.5%	2.6	
5S	298C03	54.7%	3.2	
5S	149J17	40.4%	4.2	
5S	Eu/Het border	40.0%	4.3	
5S	116D11	30.2%	5.0	
5S	282A06	28.3%	5.1	
5S	255N21	27.6%	5.1	
5S	303C11	20.5%	5.6	
5S	107A04	19.1%	5.7	

5S	011G20	7.9%	6.5	
	Centromere	0.0%	7.1	
5L	SL_MboI0058F07	8.5%	7.7	
5L	164O03	10.2%	7.9	
5L	091D14	18.8%	8.5	
5L	033M02	27.6%	9.2	
**5L**	**002J04**	**27.8%**	**9.2**	**S1**
**5L**	**SL_s0031L05**	**29.5%**	**9.3**	**S2**
**5L**	**SL_s0084H11**	**32.4%**	**9.5**	**S2**
**5L**	**016D20**	**34.1%**	**9.7**	**S3**
5L	SL_EcoRI0012L12	34.1%	9.7	
5L	Eu/Het border	40.0%	10.1	
5L	006D05	43.4%	10.4	
5L	169M21	62.6%	11.8	
5L	138J03	72.4%	12.5	
5L	100I16	95.5%	14.3	
5L	251J13	98.0%	14.5	
**5L**	**SL_EcoRI0004N07**	**99.3%**	**14.5**	**S3**
5L	Telomere	100.0%	14.6	
6S	Telomere	100.0%	0.0	
**6S**	**SL_MboI0032G06**	**98.8%**	**0.0**	**S1**
6S	251G05	76.6%	0.9	
6S	250I21	63.7%	1.3	
6S	024L21	61.4%	1.4	

6S	095C08	56.8%	1.6	
6S	SL_MboI0134P07	51.8%	1.8	
6S	Eu/Het border	49.0%	1.9	
6S	008F19	48.9%	1.9	
6S	097D13	39.4%	2.2	
**6S**	**028K02**	**36.4%**	**2.4**	**S1**
6S	SL_EcoRI0056L08	32.6%	2.5	
6S	SL_EcoRI0047D19	31.7%	2.5	
6S	SL_EcoRI0005K11	30.9%	2.6	
6S	SL_EcoRI0031A01	30.8%	2.6	
6S	SL_EcoRI0035D19	30.5%	2.6	
6S	SL_MboI0049I18	29.8%	2.6	
6S	LE_HBa0029E01	29.6%	2.6	
6S	027D17	28.9%	2.6	
6S	176D13	28.7%	2.6	
6S	078K13	28.7%	2.6	
6S	002P09	28.6%	2.6	
6S	SL_MboI0016K18	28.1%	2.7	
**6S**	**025N04**	**27.8%**	**2.7**	**S3**
6S	SL_EcoRI0021L01	27.6%	2.7	
6S	001E08	27.3%	2.7	
6S	IGS-derived satellite	27.1%	2.7	
**6S**	**SL_MboI0029F23**	**26.4%**	**2.7**	**S3**
6S	SL_EcoRI0013G12	25.8%	2.7	

**6S**	**SL_EcoRI0040C12**	**23.9%**	**2.8**	**S2**
6S	SL_MboI0081L19	17.7%	3.0	
6S	068M22	15.2%	4.6	
6S	057J04	2.7%	3.3	
	Centromere	0.0%	3.7	
**6L**	**SL_EcoRI0013C07**	**3.4%**	**4.1**	**S2**
**6L**	**SL_MboI0029C07**	**3.9%**	**4.2**	**S4**
6L	031J01	5.6%	4.4	
6L	SL_MboI0034N14	6.1%	4.5	
6L	188N10	13.2%	5.4	
**6L**	**SL_EcoRI0009J17**	**16.5%**	**5.8**	**S4**
**6L**	**SL_EcoRI0033O20**	**16.7%**	**5.8**	**S5**
6L	SL_MboI0018M19	16.9%	5.8	
6L	SL_EcoRI0040G06	17.7%	5.9	
**6L**	**031J01**	**17.9%**	**6.0**	**S5**
**6L**	**SL_MboI0034N14**	**18.1%**	**6.0**	**S6**
6L	SL_MboI0029D03	18.7%	6.1	
6L	SL_EcoRI0001M14	19.8%	6.2	
6L	SL_EcoRI0027F24	19.9%	6.2	
6L	055B14	20.9%	6.3	
6L	Eu/Het border	21.0%	6.4	
**6L**	**SL_EcoRI0023E10**	**37.1%**	**8.4**	**S6**
**6L**	**SL_EcoRI0018M04**	**39.2%**	**8.7**	**S7**
6L	SL_MboI0030C09	57.0%	10.9	

6L	081D06	60.1%	11.3	
6L	146O07	80.6%	13.9	
**6L**	**SL_EcoRI0008B16**	**88.2%**	**14.9**	**S7**
**6L**	**LE_HBa0025F06**	**90.0%**	**15.1**	**S8**
6L	068M14	91.0%	15.3	
6L	184G14	96.1%	15.9	
6L	098L02	98.1%	16.2	
**6L**	**SL_EcoRI0019F04**	**99.5%**	**16.3**	**S8**
6L	Telomere	100.0%	16.4	
7S	Telomere	100.0%	0.0	
7S	193E01	99.1%	0.1	
**7S**	**SL_EcoRI0006L14**	**98.0%**	**0.1**	**S1**
7S	002D20	97.5%	0.1	
7S	095C18	97.1%	0.2	
7S	007H24	96.6%	0.2	
7S	111F22	95.5%	0.3	
7S	150H03	94.5%	0.3	
7S	SL_EcoRI0042G10	83.0%	1.0	
7S	SL_EcoRI0025E06	57.1%	2.4	
7S	SL_MboI0137E08	52.0%	2.7	
7S	173A21	50.2%	2.8	
7S	SL_EcoRI0008H22	47.2%	3.0	
7S	SL_MboI0024O10	46.3%	3.0	
7S	325D07	44.0%	3.1	

7S	Eu/Het border	40.0%	3.4	
7S	293I23	33.0%	3.8	
	Centromere	0.0%	5.6	
**7L**	**SL_MboI0016H01**	**10.0%**	**6.6**	**S1**
**7L**	**032J14**	**10.6%**	**6.7**	**S2**
7L	003K20	13.6%	7.0	
7L	082D04	15.4%	7.2	
7L	027F11	19.3%	7.6	
7L	SL_EcoRI0021M11	19.5%	7.6	
**7L**	**SL_EcoRI0016C19**	**19.7%**	**7.6**	**S2**
7L	SL_EcoRI0041L20	19.9%	7.6	
7L	SL_EcoRI0041L23	19.9%	7.6	
7L	042K23	20.5%	7.7	
7L	036D05	21.1%	7.8	
7L	025A06	21.3%	7.8	
7L	SL_MboI0017P20	21.5%	7.8	
7L	SL_MboI0017I04	21.5%	7.8	
**7L**	**001O15**	**22.5%**	**7.9**	**S3**
7L	012C17	23.1%	8.0	
7L	034B22	27.8%	8.5	
7L	188B22	29.9%	8.7	
7L	224G23	32.1%	8.9	
7L	Eu/Het border	34.0%	9.1	
7L	041L08	36.3%	9.3	

7L	106F06	38.4%	9.6	
7L	SL_MboI0141H03	40.5%	9.8	
7L	SL_EcoRI0101G19	42.5%	10.0	
7L	SL_EcoRI0095F20	75.0%	13.3	
**7L**	**SL_EcoRI0032D11**	**77.5%**	**13.6**	**S3**
**7L**	**024C10**	**78.8%**	**13.7**	**S4**
7L	308M01	83.0%	14.1	
7L	079F09	84.0%	14.2	
7L	227C07	86.2%	14.5	
7L	178O02	88.7%	14.7	
7L	130B18	89.0%	14.8	
7L	226J04	89.4%	14.8	
7L	059P18	90.3%	14.9	
7L	261J23	91.9%	15.1	
7L	167K07	98.0%	15.7	
7L	179K09	98.0%	15.7	
**7L**	**SL_MboI0017L19**	**98.2%**	**15.7**	**S4**
7L	215P04	99.7%	15.9	
7L	Telomere	100.0%	15.9	
8S	Telomere	100.0%	0.0	
**8S**	**030O09**	**98.5%**	**0.1**	**S1**
8S	025I17	87.1%	0.6	
8S	216M19	85.9%	0.7	
8S	270A17	82.8%	0.8	

**8S**	**008J16**	**68.5%**	**1.5**	**S1**
**8S**	**015E06**	**62.8%**	**1.8**	**S2**
8S	Eu/Het border	45.0%	2.7	
**8S**	**009K19**	**38.5%**	**3.0**	**S2**
8S	SL_EcoRI0002D11	36.5%	3.1	
8S	LE_HBa0041G04	35.7%	3.2	
**8S**	**SL_MboI0012I24**	**35.2%**	**3.2**	**S7**
8S	SL_MboI0041D13	34.9%	3.2	
8S	IGS-derived satellite	34.6%	3.2	
**8S**	**SL_s0093G06**	**33.0%**	**3.3**	**S5**
**8S**	**SL_s0030B23**	**28.3%**	**3.5**	**S5**
**8S**	**SL_s0105O13**	**27.4%**	**3.6**	**S6**
	Centromere	0.0%	4.9	
8L	SL_EcoRI0008F12	3.1%	5.3	
8L	SL_MboI0012P13	3.7%	5.3	
**8L**	**SL_MboI0005G10**	**7.7%**	**5.8**	**S6**
**8L**	**SL_s0078C24**	**8.0%**	**5.8**	**S3**
8L	213E05	11.8%	6.3	
**8L**	**SL_s0058J24**	**17.0%**	**6.9**	**S3**
**8L**	**SL_EcoRI0024O12**	**18.2%**	**7.0**	**S4**
8L	034L18	23.4%	7.6	
8L	SL_MboI0019D14	24.6%	7.8	**S4**
8L	SL_MboI0012P13	25.7%	7.9	
8L	033A16	26.0%	7.9	**S8**

8L	027D09	27.6%	8.1	
8L	SL_EcoRI0027A06	31.4%	8.6	
8L	SL_EcoRI0039B17	32.0%	8.6	
8L	Eu/Het border	32.0%	8.6	
8L	160L02	32.1%	8.7	
8L	225C20	33.1%	8.8	
8L	076I13	38.3%	9.4	
8L	Chromomere	58.6%	11.8	
**8L**	**SL_MboI0007C12**	**58.7%**	**11.8**	**S8**
**8L**	**SL_s0082B11**	**60.2%**	**11.9**	**S9**
8L	005L01	99.6%	16.6	
8L	SL_EcoRI0040H01	99.7%	16.6	**S9**
8L	Telomere	100.0%	16.6	
9S	Telomere	100.0%	0.0	
**9S**	**SL_MboI0032A02**	**99.4%**	**0.0**	**S1**
9S	026I24	99.3%	0.0	
9S	072G22	88.9%	0.6	
9S	116C14	84.9%	0.8	
9S	168F14	77.9%	1.2	
9S	203J14	77.6%	1.3	
9S	300E15	64.5%	2.0	
9S	026P14	63.4%	2.1	
9S	255E01	56.9%	2.4	
9S	SL_MboI0080E11	48.9%	2.9	

9S	300E15	47.5%	2.9	
9S	026P14	47.3%	3.0	
9S	308C20	41.6%	3.3	
9S	Eu/Het border	40.0%	3.4	
9S	197E10	28.3%	4.0	
9S	SL_MboI0056H18	9.4%	5.1	
9S	072H18	6.6%	5.2	
**9S**	**SL_MboI0025N23**	**5.8%**	**5.3**	**S1**
9S	SL_MboI0045G03	3.4%	5.4	
9S	SL_EcoRI0052D12	3.4%	5.4	
**9S**	**SL_EcoRI0015A15**	**2.3%**	**5.5**	**S4**
	Centromere	0.0%	5.6	
**9L**	**SL_EcoRI0025E12**	**7.2%**	**6.3**	**S4**
**9L**	**SL_EcoRI0018E08**	**8.2%**	**6.4**	**S3**
9L	061J21	11.0%	6.7	
9L	075M11	11.8%	6.8	
**9L**	**LE_HBa0005G01**	**13.5%**	**7.0**	**S3**
**9L**	**SL_MboI0013P10**	**15.0%**	**7.1**	**S5**
**9L**	**SL_EcoRI0022O16**	**15.3%**	**7.2**	**S5**
**9L**	**SL_MboI0018I03**	**16.9%**	**7.3**	**S6**
9L	107D15	31.3%	8.8	
9L	Eu/Het border	34.0%	9.1	
9L	099F14	61.2%	11.9	
**9L**	**LE_HBa0005M15**	**62.7%**	**12.1**	**S6**

9L	107P11	63.7%	12.2	
**9L**	**SL_FOS0051C04**	**64.2%**	**12.2**	**S2**
**9L**	**SL_MboI0019G06**	**64.6%**	**12.3**	**S7**
9L	099P03	70.7%	12.9	
9L	278J12	71.6%	13.0	
9L	248I10	72.3%	13.0	
**9L**	**SL_MboI0020M04**	**74.5%**	**13.3**	**S7**
**9L**	**SL_MboI0025N11**	**77.9%**	**13.6**	**S8**
**9L**	**LE_HBa0010F04**	**81.6%**	**14.0**	**S8**
**9L**	**LE_HBa0001I06**	**82.7%**	**14.1**	**S9**
9L	245E05	88.2%	14.7	
**9L**	**SL_EcoRI0024O21**	**91.9%**	**15.1**	**S9**
**9L**	**SL_MboI0037I08**	**95.3%**	**15.4**	**S10**
9L	165P17	95.4%	15.4	
9L	109D11	97.5%	15.6	
**9L**	**SL_MboI0022L14**	**99.2%**	**15.8**	**S10**
9L	SL_MboI0042F10	99.6%	15.9	
9L	Telomere	100.0%	15.9	
10S	Telomere	100.0%	0.0	
**10S**	**SL_EcoRI0027L04**	**98.5%**	**0.1**	**S1**
10S	060A06	91.6%	0.4	
10S	091N19	88.4%	0.6	
10S	012D17	87.3%	0.6	
10S	176H22	87.2%	0.6	

10S	155I21	76.8%	1.1	
10S	222H10	75.4%	1.2	
10S	SL_EcoRI0076M19	70.5%	1.4	
10S	028O04	69.9%	1.4	
10S	041K23	56.3%	2.1	
**10S**	**SL_EcoRI0009D07**	**52.3%**	**2.3**	**S1**
**10S**	**023E16**	**51.7%**	**2.3**	**S2**
10S	201C12	50.7%	2.4	
10S	Eu/Het border	46.0%	2.6	
10S	205L07	30.5%	3.3	
10S	SL_MboI0020C04	27.5%	3.5	
10S	036L10	23.2%	3.7	
10S	111D09	19.1%	3.9	
10S	105C09	17.5%	4.0	
10S	SL_EcoRI0040D01	14.5%	4.1	
**10S**	**SL_s0071N16**	**11.3%**	**4.3**	**S2**
10S	237A02	10.8%	4.3	
**10S**	**SL_EcoRI0029F05**	**9.6%**	**4.3**	**S4**
	Centromere	0.0%	4.8	
**10L**	**SL_MboI0011N01**	**6.2%**	**5.4**	**S4**
**10L**	**SL_s0042K13**	**6.5%**	**5.5**	**S3**
10L	115K16	10.8%	5.9	
10L	114B06	16.6%	6.5	
10L	043B12	26.3%	7.5	

10L	037N23	26.4%	7.5	
10L	Eu/Het border	33.0%	8.1	
10L	044O20	39.6%	8.8	
10L	045L17	43.8%	9.2	
10L	053L19	49.1%	9.8	
**10L**	**SL_s0121P17**	**53.2%**	**10.2**	**S3**
**10L**	**SL_FOS0014E24**	**54.3%**	**10.3**	**S5**
**10L**	**SL_EcoRI0036N16**	**55.3%**	**10.4**	**S6**
10L	SL_EcoRI0013B18	56.3%	10.5	
10L	204I05	57.7%	10.6	
10L	189B10	58.5%	10.7	
10L	022B11	58.7%	10.7	
10L	044H12	61.7%	11.0	
10L	188N09	62.1%	11.1	
10L	071D20	62.9%	11.1	
10L	020A12	63.1%	11.2	
10L	248A13	63.5%	11.2	
10L	234C10	64.1%	11.3	
10L	156C03	64.6%	11.3	
10L	057G01	71.3%	12.0	
10L	206B16	71.8%	12.0	
10L	005D06	76.1%	12.5	
10L	256L16	78.5%	12.7	
10L	021O12	79.2%	12.8	

10L	334K22	82.8%	13.2	
10L	049K02	86.1%	13.5	
10L	011E16	89.9%	13.9	
10L	181E17	94.7%	14.4	
10L	013B20	96.0%	14.5	
**10L**	**SL_EcoRI0008A07**	**99.6%**	**14.9**	**S6**
10L	Telomere	100.0%	14.9	
11S	Telomere	100.0%	0.0	
**11S**	**SL_s0029B21**	**98.6%**	**0.1**	**S1**
11S	168B23	97.2%	0.2	
11S	024K09	63.9%	2.3	
11S	027B05	63.6%	2.4	
11S	015A13	58.0%	2.7	
11S	SL_MboI121I03	42.2%	3.8	
11S	034I10	41.8%	3.8	
11S	214E16	41.4%	3.8	
11S	080C09	41.0%	3.8	
11S	128G07	40.5%	3.9	
11S	064J13	39.9%	3.9	
11S	291F09	39.2%	4.0	
11S	SL_MboI0052K14	37.7%	4.1	
11S	Eu/Het border	32.0%	4.4	
**11S**	**SL_EcoRI0018D15**	**17.9%**	**5.3**	**S1**
**11S**	**008F06**	**16.7%**	**5.4**	**S4**

11S	023H04	13.7%	5.6	
**11S**	**004F15**	**11.7%**	**5.7**	**S4**
11S	IGS-derived satellite	11.0%	5.8	
**11S**	**SL_s0082L15**	**10.1%**	**5.8**	**S2**
	Centromere	0.0%	6.5	
11L	305L18	6.6%	7.0	
11L	187P23	7.4%	7.1	
11L	SL_EcoRI0045G19	8.0%	7.1	
11L	SL_EcoRI0027A06	12.6%	7.5	
**11L**	**001N12**	**13.7%**	**7.6**	**S2**
11L	SL_MboI0047F15	14.3%	7.6	
11L	062E02	15.2%	7.7	
**11L**	**SL_EcoRI0031L09**	**16.2%**	**7.8**	**S3**
11L	Eu/Het border	43.0%	9.9	
**11L**	**SL_s0045H10**	**44.4%**	**10.1**	**S3**
**11L**	**SL_s0084O21**	**45.6%**	**10.1**	**S5**
11L	Mitochondrial DNA	47.3%	10.3	
**11L**	**SL_s0017L16**	**48.1%**	**10.3**	**S5**
**11L**	**SL_s0105E01**	**50.4%**	**10.5**	**S6**
11L	245M17	50.7%	10.6	
11L	158K02	51.5%	10.6	
11L	119D16	56.6%	11.0	
11L	316E10	58.3%	11.2	
11L	249E07	66.8%	11.8	

11L	323E19	88.2%	13.6	
**11L**	**SL_s0014A19**	**98.3%**	**14.4**	**S6**
11L	Telomere	100.0%	14.5	
12S	Telomere	100.0%	0.0	
**12S**	**SL_MboI0030P17**	**99.3%**	**0.0**	**S1**
12S	045N22	96.8%	0.2	
12S	026C13	93.7%	0.4	
12S	163O04	82.7%	1.2	
12S	146I19	78.9%	1.5	
12S	049J09	69.3%	2.2	
12S	075A23	67.9%	2.3	
12S	180J10	65.9%	2.4	
12S	206N09	64.7%	2.5	
12S	105C12	56.9%	3.1	
12S	154D06	50.4%	3.5	
12S	Eu/Het border	40.0%	4.3	
**12S**	**SL_EcoRI0005K14**	**38.9%**	**4.3**	**S1**
**12S**	**LE_HBa0016B06**	**37.9%**	**4.4**	**S8**
12S	SL_MboI0050G12	36.9%	4.5	
**12S**	**SL_MboI0038L04**	**36.3%**	**4.5**	**S8**
12S	SL_EcoRI0010P04	35.8%	4.6	
**12S**	**SL_EcoRI0036O23**	**35.7%**	**4.6**	**S7**
**12S**	**SL_EcoRI0003D15**	**31.1%**	**4.9**	**S7**
**12S**	**SL_MboI0008A07**	**29.9%**	**5.0**	**S2**

**12S**	**003H12**	**22.2%**	**5.5**	**S2**
**12S**	**SL_MboI0023C14**	**21.0%**	**5.6**	**S6**
**12S**	**SL_EcoRI0004B02**	**18.9%**	**5.8**	**S6**
**12S**	**SL_MboI0040D19**	**17.6%**	**5.9**	**S5**
**12S**	**SL_MboI0019O03**	**7.7%**	**6.6**	**S3**
12S	005D14	7.0%	6.6	
**12S**	**SL_EcoRI0001K10**	**4.2%**	**6.8**	**S5**
12S	SL_EcoRI0012H19	3.4%	6.9	
12S	012P02	0.0%	7.1	
	Centromere	0.0%	7.1	
12L	SL_EcoRI0012H19	2.2%	7.3	**Heinz 1706 S5**
12L	005D14	3.7%	7.4	**Heinz 1706 S3**
12L	153L10	7.4%	7.7	
12L	148K11	16.1%	8.3	
12L	006G23	23.6%	8.9	
12L	047D08	27.6%	9.2	
12L	SL_MboI0009A03	29.5%	9.3	
12L	SL_MboI0128K09	31.6%	9.5	
12L	331D02	32.1%	9.5	
12L	152A18	35.4%	9.8	
12L	Eu/Het border	40.0%	10.1	
**12L**	**SL_MboI0035N06**	**60.2%**	**11.6**	**S3**
**12L**	**SL_MboI0006L17**	**61.0%**	**11.7**	**S4**

**12L**	**017P17**	**65.1%**	**12.0**	**S4**
12L	079O22	66.6%	12.1	
12L	SL_MboI0011A16	69.3%	12.3	
**12L**	**SL_EcoRI0031M18**	**73.1%**	**12.6**	**S9**
12L	012E19	76.4%	12.8	
**12L**	**SL_EcoRI0024H05**	**79.0%**	**13.0**	**S9**
**12L**	**LE_HBa0029L21**	**80.6%**	**13.1**	**S10**
12L	SL_EcoRI0045D23	81.0%	13.2	
12L	326K10	82.4%	13.3	
12L	093P12	87.0%	13.6	
12L	055G18	95.7%	14.3	
12L	116D04	97.9%	14.4	
**12L**	**LE_HBa0030J22**	**98.7%**	**14.5**	**S10**
12L	Telomere	100.0%	14.6	
