# Supplementary material for: Fluorescence In Situ Hybridization and Optical Mapping to Correct Scaffold Arrangement in the Tomato Genome
Source: G3 (Bethesda). 2014 May 30;4(8):1395–405. doi: 10.1534/g3.114.011197 (PMC4132171; doi:10.1534/g3.114.011197)
Supplement: Supporting Information [file supp_g3.114.011197_TableS4.pdf]

**Table S4 FISH-based scaffold order, orientation, size, and chromatin type**

| Chr. | Scaffold Name[1,2] | Scaffold Number[2,3] | Left (head)[4] BAC Probe[5] | Right (tail)[4] BAC Probe[5] | Scaffold Size (Mb)[6] | SC len. (µm) | Chromatin Type[11] |
|---|---|---|---|---|---|---|---|
| 1 | SL2.40sc04133 | 1 | s090M22 | M034D03 | 33.0 | 5.8 | Eu/Het |
| 1 | SL2.40sc04191 | 3 | s053P14 | s083L21 | 1.7 | 0.2 | Het |
| 1 | SL2.40sc03666 | 2 | E016I11 | H033N15 | 2.6 | 0.2 | Het |
| 1 | SL2.40sc03594 | 4 | s006A13 | s042B18 | 6.3 | 0.7 | Het |
| 1 | SL2.40sc05010 | 5 | s042O02 | H037N04 | 20.5 | 2.9 | Het/Border |
| 1 | SL2.40sc05941 | 6 | s040G18 | E021C24 | 6.2 | 1.7 | Eu/Border |
| 1 | SL2.40sc06903 | 8 | s022L14 | s121I01 | 2.7 | 1.1 | Eu |
| 1 | SL2.40sc06917 | 7 | s071P10 | n/a[7] | 0.4 | n/a | Eu |
| 1 | SL2.40sc04323 | 9 | s024J19 | M028C09 | 17.0 | 12.6 | Eu |
| 2 | SL2.40sc04732 | 4 | E007F19 | E008B15 | 1.7 | 0.2 | Het |
| 2 | SL2.40sc04208 | 6 | s053E24 | s044P10 | 1.4 | 0.2 | Het |
| 2 | SL2.40sc05776 | 5 | H011G24 | M036C03 | 2.1 | 0.5 | Het |
| 2 | SL2.40sc06593 | 1 | s056I16 | s079J24 | 4.0 | 0.5 | Het |
| 2 | SL2.40sc04142 | 2 | s053C07 | s050E09 | 6.5 | 1.5 | Het/Cent |
| 2 | SL2.40sc03766 | 3 | M021A03 | E034J17 | 11.7 | 2.5 | Het/Border |
| 2 | SL2.40sc03665 | 7 | s101F18 | M017J13 | 22.4 | 15.3 | Eu |
| 3 | SL2.40sc04439 | 1 | s009C01 | s086D22 | 2.5 | 1.4 | Eu |
| 3 | SL2.40sc04696 | 4 | s018K15 | s002G24 | 1.3 | 0.5 | Eu |
| 3 | SL2.40sc05330 | 5 | s050E05 | H037B06 | 2.6 | 0.9 | Eu/Border |
| 3 | SL2.40sc04126 | 6 | H020G20 | H039C15 | 6.5 | 0.9 | Het/Border |
| 3 | SL2.40sc04616 | 9 | M079O10 | M002J15 | 3.1 | 0.6 | Cent/Het |
| 3 | SL2.40sc06725 | 7 | H244B01 | E121G21 | 3.0 | 0.3 | Het |
| 3 | SL2.40sc04704 | 8 | H027L13 | s071G06 | 18.6 | 2.2[8] | Het |
| 3 | SL2.40sc03721 | 12 | E018K21 | H028L03 | 1.1 | 0.0[8] | Het/Border |
| 3 | SL2.40sc04822 | 2 | s042B23 | H030A19 | 4.1 | 0.5[8] | Het/Border |
| 3 | SL2.40sc03806 | 10 | H011C02 | H001H05 | 5.5 | 1.8[8] | Border/Eu |
| 3 | SL2.40sc03796 | 11 | M003H09 | E031G05 | 7.0 | 2.4 | Eu |
| 3 | SL2.40sc06911 | 3 | M015G06 | s048H23 | 0.4 | 0.0 | Eu |
| 3 | SL2.40sc03701 | 13 | E006F17 | E034G21 | 9.2 | 6.2 | Eu |
| 4 | SL2.40sc03604 | 1 | M035I14 | H012B20 | 11.6 | 4.2 | Eu/Het |
| 4 | SL2.40sc05339 | 3 | E036C15 | H018I03 | 2.0 | 0.6 | Het/Cent |
| 4 | SL2.40sc03683 | 2 | M036H17 | M016J15 | 17.6 | 2.5 | Cent/Het |
| 4 | SL2.40sc06101 | 4 | M038M16 | M029O16 | 23.3 | 4.6 | Het/Eu |
| 4 | SL2.40sc04680 | 5 | H013P02 | M018D12 | 0.8 | 0.4 | Eu |

| | | | | | | | |
|---|---|---|---|---|---|---|---|
| 4 | SL2.40sc04135 | 6 | H020C13 | M023M21 | 8.8 | 5.2 | Eu |
| 5 | SL2.40sc03726 | 1 | s094J05 | H002J04 | 42.1 | 9.1 | Eu/Het/Cent |
| 5 | SL2.40sc06155 | 2 | s031L05 | s084H11 | 4.3 | 0.2 | Het |
| 5 | SL2.40sc03902 | 3 | H016D20 | E004N07 | 18.6 | 4.9 | Eu/Het |
| 6 | SL2.40sc04474 | 1 | M032G6 | H028K02 | 8.6 | 2.3 | Eu/Het |
| 6 | SL2.40sc06140 | 3 | H025N04 | M029F23 | 0.7 | 0.1 | Het |
| 6 | SL2.40sc05383 | 2 | E040C12 | E013C07 | 6.1 | 1.3 | Het/Cent |
| 6 | SL2.40sc04279 | 4 | M029C07 | E009J17 | 9.6 | 1.6 | Het |
| 6 | SL2.40sc05188 | 5 | E033O20 | H031J01 | 2.4 | 0.2 | Border |
| 6 | SL2.40sc05732 | 6 | M034N10 | E023E10 | 6.6 | 2.4 | Border/Eu |
| 6 | SL2.40sc05054 | 7 | E018M04 | E008B16 | 10.3 | 6.2 | Eu |
| 6 | SL2.40sc03622 | 8 | H025F06 | E019F04 | 1.8 | 1.2 | Eu |
| 7 | SL2.40sc03731 | 1 | E006L14 | M016H01 | 33.3 | 6.5 | Eu/Het/Cent |
| 7 | SL2.40sc05397 | 2 | H032J14 | E016C19 | 8.0 | 0.9 | Het |
| 7 | SL2.40sc03685 | 3 | H001O15 | E032D11 | 20.7 | 5.7 | Het/Eu |
| 7 | SL2.40sc04626 | 4 | H024C01 | M017L19 | 3.4 | 2.0 | Eu |
| 8 | SL2.40sc04813 | 1 | H030O09 | H008J16 | 3.0 | 1.5 | Eu |
| 8 | SL2.40sc03770 | 2 | H015E06 | H009K19 | 6.3 | 1.2 | Eu/Border |
| 8 | SL2.40sc04167 | 7 | M012I24 | n/a[7] | 0.2 | n/a | Het |
| 8 | SL2.40sc03749 | 5 | s093G06 | s030B23 | 1.5 | 0.2 | Het |
| 8 | SL2.40sc04236 | 6 | s105O13 | M005G10 | 14.6 | 2.2 | Het/Cent |
| 8 | SL2.40sc03835 | 3 | s078C24 | s058J24 | 8.4 | 1.1 | Het |
| 8 | SL2.40sc04701 | 4 | E024O12 | M019D14 | 7.0 | 0.7 | Het |
| 8 | SL2.40sc04948 | 8 | H033A16 | M007C12 | 14.4 | 3.8 | Eu/Het |
| 8 | SL2.40sc03923 | 9 | s082B11 | E040H01 | 7.5 | 4.6 | Eu |
| 9 | SL2.40sc03771 | 1 | M032A02 | M025N23 | 19.2 | 5.2 | Eu/Het |
| 9 | SL2.40sc04008 | 4 | E015A15 | E025E12 | 5.2 | 0.9 | Het/Cent |
| 9 | SL2.40sc04950 | 3 | E018E08 | M005G01 | 5.8 | 0.5 | Het |
| 9 | SL2.40sc04785 | 5 | M013P10 | E022O16 | 2.0 | 0.0 | Het |
| 9 | SL2.40sc04777 | 6 | M018I03 | H005M15 | 28.2 | 4.7 | Eu/Het |
| 9 | SL2.40sc06916 | 2 | F051C04 | n/a[7] | 0.0[9] | n/a | Eu |
| 9 | SL2.40sc05269 | 7 | M019G06 | M020M04 | 2.7 | 1.0 | Eu |
| 9 | SL2.40sc03852 | 8 | M025N11 | H010F04 | 1.3 | 0.4 | Eu |
| 9 | SL2.40sc04828 | 9 | H001I06 | E024O21 | 2.5 | 0.9 | Eu |
| 9 | SL2.40sc06214 | 10 | M037I08 | M022L14 | 0.6 | 0.4 | Eu |
| 10 | SL2.40sc05925 | 1 | E027L04 | E009D07 | 5.4 | 2.2 | Eu/Border |
| 10 | SL2.40sc03798 | 2 | H023E16 | s071N16 | 16.5 | 1.9 | Het |
| 10 | SL2.40sc04872 | 4 | E029F05 | M011N01 | 3.9 | 1.1 | Cent |
| 10 | SL2.40sc05632 | 3 | s042K13 | s121P17 | 31.1 | 4.7 | Eu/Het |

| | | | | | | | |
|---|---|---|---|---|---|---|---|
| 10 | SL2.40sc04534 | 5 | F014E24 | n/a[7] | 0.1 | n/a | Eu |
| 10 | SL2.40sc04199 | 6 | E036N16 | E008A07 | 8.0 | 4.5 | Eu |
| 11 | SL2.40sc03748 | 1 | s029B21 | E018D15 | 15.2 | 5.2 | Eu/Het |
| 11 | SL2.40sc06763 | 4 | H008F06 | H004F15 | 2.5 | 0.3 | Het |
| 11 | SL2.40sc04054 | 2 | s082L15 | H001N12 | 8.0 | 1.8 | Het/Cent |
| 11 | SL2.40sc03752 | 3 | E031L09 | s045H10 | 17.4 | 2.3 | Het/Border |
| 11 | SL2.40sc06137 | 5 | s084O21 | s017L16 | 2.1 | 0.2 | Border |
| 11 | SL2.40sc03876 | 6 | s105E01 | s014A19 | 8.2 | 3.8 | Eu |
| 12 | SL2.40sc04607 | 1 | M030P17 | E005K14 | 16.1 | 4.3 | Eu/Border |
| 12 | SL2.40sc06147 | 8 | H016B06 | M038L04 | 1.2 | 0.1 | Border |
| 12 | SL2.40sc04039 | 7 | E036O23 | E003D15 | 4.9 | 0.3 | Border |
| 12 | SL2.40sc04878 | 2 | M008A07 | H003H12 | 5.7 | 0.5 | Het |
| 12 | SL2.40sc04266 | 6 | M023C14 | E004B02 | 1.3 | 0.1 | Het |
| 12 | SL2.40sc04757[10] | 5 | M040D19 | E012H19 | 5.8 | 1.4 | Het/Cent |
| 12 | SL2.40sc04057 | 3 | H005D14 | M035N06 | 25.2 | 4.2 | Eu/Het |
| 12 | SL2.40sc04915 | 4 | M006L17 | H017P17 | 1.6 | 0.3 | Eu |
| 12 | SL2.40sc05611 | 9 | E031M18 | E024H05 | 1.2 | 0.4 | Eu |
| 12 | SL2.40sc05380 | 10 | H029L21 | E030J22 | 2.6 | 1.4 | Eu |
| | | | | Total: | 760.0 | 196.7 | |

[1] http://solgenomics.net/cview/map.pl?map_id=agp

[2] FISH-based scaffolds are color coded with reference to a comparison with their order and orientation determined using the Kazusa EXPEN 2000 linkage map. Scaffold names and numbers that are unchanged by FISH are shown with black lettering on a white background. Changed scaffolds are shown with white lettering on a red background (order only), white lettering on a blue background (orientation only), and white lettering on a purple background (both order and orientation).

[3] Scaffold number is determined by scaffold order from head to tail in the pseudomolecules based on the linkage map.

[4] Left and right refer to the probe orientation when the chromosomes are diagramed with the telomere of the short arm on the left (= head) and the telomere of the long arm on the right (= tail).

[5] BAC library names have been abbreviated. H=HindIII, E=EcoRI, M=MboI, s=sheared, F=Fosmid

[6] The Tomato Genome Consortium, 2012 The tomato genome sequence provides insights into fleshy fruit evolution. Nature 485: 635-641; http://solgenomics.net

[7] Not applicable because only one BAC/fosmid was localized in this small scaffold.

[8] SC length determinations are relatively inaccurate in and near scaffolds 12 and 2.

[9] The actual length of this scaffold is 17.6 Kb so it is rounded to 0.0 Mb.

[10] Scaffold SL2.40sc04757 was ordered and oriented in Heinz 1706 because this scaffold was involved in an inversion around the kinetochore of Cherry Tomato LA4444.

11 Eu = euchromatin; Het = heterochromatin; cent = centromere/kinetochore; Border (transition between euchromatin and heterochromatin); Eu/Het includes euchromatin, heterochromatin, and the border between Eu and Het; Het/Cent includes heterochromatin and centromere; Border/Eu includes euchromatin and border with heterochromatin; Eu/Het/Cent includes euchromatin, the border with heterochromatin, heterochromatin and centromeres.
