# Supplementary material for: Fluorescence In Situ Hybridization and Optical Mapping to Correct Scaffold Arrangement in the Tomato Genome
Source: G3 (Bethesda). 2014 May 30;4(8):1395–405. doi: 10.1534/g3.114.011197 (PMC4132171; doi:10.1534/g3.114.011197)
Supplement: Supporting Information [file supp_g3.114.011197_TableS5.pdf]

Table S5 Comparison of scaffold order and orientation based on linkage, optical mapping, and FISH

Chromosome	Scaffold No.[1]	Scaffold Name[2]	Mb Length	Scaffold Order According to:			
				Linkage	Optical Superscaffold (OS)[3]		FISH[4]
1	1	SL2.40sc04133	33.0	1			1
1	2	SL2.40sc03666	1.7	2			3
1	3	SL2.40sc04191	2.6	3	2	OS 1	2*
1	4	SL2.40sc03594	6.3	4	4		4
1	5	SL2.40sc05010	20.5	5	5	OS 2	5
1	6	SL2.40sc05941	6.2	6	6		6
1	7	SL2.40sc06903	2.7	7	8		8
1	8	SL2.40sc06917	0.4	8	7		7
1	9	SL2.40sc04323	17.0	9			9
2	1	SL2.40sc06593	1.7	1	4	OS 3	4*
2	2	SL2.40sc04142	1.4	2	6		6*
2	3	SL2.40sc03766	2.1	3			5
2	4	SL2.40sc04732	4.0	4			1
2	5	SL2.40sc05776	6.5	5			2
2	6	SL2.40sc04208	11.7	6			3
2	7	SL2.40sc03665	22.4	7			7
3	1	SL2.40sc04439	2.5	1			1
3	2	SL2.40sc04822	1.3	2			4
3	3	SL2.40sc06911	2.6	3			5
3	4	SL2.40sc04696	6.5	4			6
3	5	SL2.40sc05330	3.1	5			9
3	6	SL2.40sc04126	3.0	6			7
3	7	SL2.40sc06725	18.6	7			8
3	8	SL2.40sc04704	4.1	8			2*
3	9	SL2.40sc04616	1.1	9			12
3	10	SL2.40sc03806	5.5	10			10
3	11	SL2.40sc03796	7.0	11			11
3	12	SL2.40sc03721	0.4	12	3	OS 4	3*
3	13	SL2.40sc03701	9.2	13	13		13
4	1	SL2.40sc03604	11.6	1			1
4	2	SL2.40sc03683	2.0	2			3*
4	3	SL2.40sc05339	17.6	3			2*
4	4	SL2.40sc06101	23.3	4			4

4	5	SL2.40sc04680	0.8	5			5
4	6	SL2.40sc04135	8.8	6			6
5	1	SL2.40sc03726	42.1	1			1
5	2	SL2.40sc06155	4.3	2			2*
5	3	SL2.40sc03902	18.6	3			3
6	1	SL2.40sc04474	8.6	1			1
6	2	SL2.40sc05383	0.7	2			3*
6	3	SL2.40sc06140	6.1	3			2
6	4	SL2.40sc04279	9.6	4			4
6	5	SL2.40sc05188	2.4	5	5		5*
6	6	SL2.40sc05732	6.6	6	6		6
6	7	SL2.40sc05054	10.3	7	7	OS 5	7
6	8	SL2.40sc03622	1.8	8	8		8
7	1	SL2.40sc03731	33.3	1	1		1
7	2	SL2.40sc05397	8.0	2	2	OS 6	2
7	3	SL2.40sc03685	20.7	3			3
7	4	SL2.40sc04626	3.4	4			4
8	1	SL2.40sc04813	3.0	1	1		1
8	2	SL2.40sc03770	6.3	2	2	OS 7	2
8	3	SL2.40sc03835	0.2	3			7
8	4	SL2.40sc04701	1.5	4	5		5*
8	5	SL2.40sc03749	14.6	5	6		6*
8	6	SL2.40sc04236	8.4	6	3	OS 8	3*
8	7	SL2.40sc04167	7.0	7	4		4*
8	8	SL2.40sc04948	14.4	8			8
8	9	SL2.40sc03923	7.5	9			9
9	1	SL2.40sc03771	19.2	1			1
9	2	SL2.40sc06916	0.0	2	4		4
9	3	SL2.40sc04950	5.2	3	3		3*
9	4	SL2.40sc04008	5.8	4	5	OS 9	5
9	5	SL2.40sc04785	2.0	5	6		6
9	6	SL2.40sc04777	28.2	6			2
9	7	SL2.40sc05269	2.7	7			7
9	8	SL2.40sc03852	1.3	8	8		8
9	9	SL2.40sc04828	2.5	9	9	OS 10	9
9	10	SL2.40sc06214	0.6	10	10		10
10	1	SL2.40sc05925	5.4	1	1		1
10	2	SL2.40sc03798	16.5	2	2	OS 11	2
10	3	SL2.40sc05632	3.9	3			4

10	4	SL2.40sc04872	31.1	4			3
10	5	SL2.40sc04534	0.1	5			5
10	6	SL2.40sc04199	8.0	6			6
11	1	SL2.40sc03748	15.2	1			1
11	2	SL2.40sc04054	2.5	2			4
11	3	SL2.40sc03752	8.0	3			2
11	4	SL2.40sc06763	17.4	4			3*
11	5	SL2.40sc06137	2.1	5	5	OS 12	5
11	6	SL2.40sc03876	8.2	6	6		6
12	1	SL2.40sc04607	16.1	1			1
12	2	SL2.40sc04878	1.2	2			8
12	3	SL2.40sc04057	4.9	3			7*
12	4	SL2.40sc04915	5.7	4			2
12	5	SL2.40sc04757	1.3	5			6
12	6	SL2.40sc04266	5.8	6	5	OS 13	5
12	7	SL2.40sc04039	25.2	7	3		3
12	8	SL2.40sc06147	1.6	8	4		4
12	9	SL2.40sc05611	1.2	9	9	OS 14	9
12	10	SL2.40sc05380	2.6	10	10		10
**Total**			760.0				

[1]Scaffolds are numbered according to their order determined using the tomato Kazusa EXPEN 2000 linkage map at http://solgenomics.net/cview/map.pl?map_version_id=103

[2] http://solgenomics.net/cview/map.pl?map_id=agp

[3]Optical superscaffolds consist of 2-4 sequenced scaffolds ordered and oriented by optical mapping. Scaffold numbers refer to column 2. Empty spaces indicate scaffolds that were not ordered and oriented by optical mapping. Green scaffolds are in the same order and orientation as that determined by the linkage map. Red scaffolds are in the same orientation but different order, blue scaffolds are in the same order but different orientation, and purple scaffolds are in a different order and orientation than that determined by the linkage map.

[4]Order and orientation of scaffolds determined by FISH. Scaffold numbers refer to column 2. Green scaffolds are in the same order and orientation by both optical mapping and by FISH.

*Denotes scaffolds with a different orientation by FISH than that determined by the linkage map.
