# Supplementary material for: Fluorescence In Situ Hybridization and Optical Mapping to Correct Scaffold Arrangement in the Tomato Genome
Source: G3 (Bethesda). 2014 May 30;4(8):1395–405. doi: 10.1534/g3.114.011197 (PMC4132171; doi:10.1534/g3.114.011197)
Supplement: Supporting Information [file supp_g3.114.011197_TableS6.pdf]

Table S6 Estimating the linear density of DNA in euchromatin, heterochromatin, and kinetochores by FISH[1]

Chromatin Type	Chr. #	Scaffold #	Scaffold Size Mb (Corrected Mb)[2]	SC Length - µm	Mb/µm
Euchromatin	1	SL2.40sc04323	17.0 (16.8)	12.6	1.33
	2	SL2.40sc03665	22.4 (21.8)	15.3	1.43
	3	SL2.40sc04439	2.5 (2.3)	1.4	1.62
	3	SL2.40sc03701	9.2 (8.7)	6.2	1.40
	4	SL2.40sc04135	8.8 (8.6)	5.2	1.65
	6	SL2.40sc05054	10.3 (9.8)	6.2	1.58
	6	SL2.40sc03622	1.8 (1.7)	1.2	1.38
	7	SL2.40sc04626	3.4 (3.2)	2.0	1.58
	8	SL2.40sc03923	7.5 (7.4)	4.6	1.60
	10	SL2.40sc04199	8.0 (7.7)	4.5	1.70
	11	SL2.40sc03876	8.2 (7.9)	3.8	2.07
	12	SL2.40sc05380	2.6 (2.3)	1.4	1.66
	**Total**		**101.7 (98.0) Mb**	**64.4 µm**	**1.52 Mb/µm[3]**
Heterochromatin	1	SL2.40sc03594	6.3 (5.9)	0.7	8.41
	7	SL2.40sc05397	8.0 (7.8)	0.9	8.69
	8	SL2.40sc04701	7.0 (6.8)	0.7	9.76
	10	SL2.40sc03798	16.5 (16.3)	1.9	8.58
	**Total**		**37.8 (36.8) Mb**	**4.2 µm**	**8.76 Mb/µm[3]**
Kinetochore	10	SL2.40sc04872	3.9 (3.6)	1.1	3.27
	**Total**		**3.9 (3.6) Mb**	**1.1 µm**	**3.27 Mb/µm[3]**

[1]Scaffold numbers, DNA amounts in scaffolds, lengths of scaffolds, and chromatin types were extracted from Table S1.

[2]The FISH signals at either end of a scaffold are not at the very ends of the scaffold but somewhat into the scaffold [see File S1, Additional Materials and Methods- Determining the amount of DNA per micrometer of SC (= linear DNA density) in kinetochores, euchromatin, and heterochromatin]. Because of this, scaffold DNA extending beyond the FISH signals must be subtracted from the length of the scaffold to yield the "Corrected Mb" between FISH signals that will be divided by SC length in micrometers to yield Mb/µm.

[3] = Total (corrected) Mb DNA ÷ total SC length (µm)
