# Supplementary material for: Fluorescence In Situ Hybridization and Optical Mapping to Correct Scaffold Arrangement in the Tomato Genome
Source: G3 (Bethesda). 2014 May 30;4(8):1395–405. doi: 10.1534/g3.114.011197 (PMC4132171; doi:10.1534/g3.114.011197)
Supplement: Supporting Information [file supp_g3.114.011197_TableS7.pdf]

Table S7 Gap sizes between scaffolds determined using BAC-FISH

Chr.	Gap between Scaffold A and Scaffold B[1]	BAC Probe for Tail of Scaffold A[2]	BAC Probe for Head of Scaffold B[2]	Chromatin Type[3]	DNA Mb per µm	Gap Size SC length (µm)	Distance between BACs (Mb)	Corrected Gap Size Mb estimate[4]
1	1-3	M034D03	s053P14	Het	8.8	0.3	2.6	2.4
1	3-2	s083L21	E016I11	Cent	3.3	0.7	2.2	1.9
1	2-4	H033N15	s006A13	Het	8.8	0.1	0.9	0.6
1	4-5	s042B18	s042O02	Het	8.8	0.3	2.6	2.2
1	5-6	H037N04	s040G18	Border	5.1	0.1	0.5	0.3
1	6-8	E021C24	s022L14	Border	5.1	0.1	0.5	0.3
1	8-7	s121I01	s071P10	Eu	1.5	0.3	0.5	0.2
1	7-9	s071P10	s024J19	Eu	1.5	0.3	0.5	0.2
2	4-6	E008B15	s053E24	Het	8.8	0.1	0.7	0.4
2	6-5	s044P10	H011G24	Het	8.8	0.0	0.2	0.0
2	5-1	M036C03	s056I16	Het	8.8	0.0	0.1	0.0
2	1-2	s079J24	s053C07	Het	8.8	0.5	4.1	3.2
2	2-3	s050E09	M021A03	Het	8.8	0.1	1.0	0.5
2	3-7	E034J17	s101F18	Border	5.1	0.4	1.9	1.4
3	1-4	s086D22	s018K15	Eu	1.5	0.2	0.4	0.1
3	4-5	s002G24	s050E05	Eu	1.5	0.1	0.2	0.0
3	5-6	H037B06	H020G20	Border	5.1	0.2	1.1	0.3
3	6-9	H039C15	M079O10	Het/Cent	8.8	0.6	5.0	4.7
3	9-7	M002J15	H244B01	Het	8.8	0.1	0.6	0.2
3	7-8	E121G21	H027L13	Het	8.8	0.1	1.2	0.8
3	8-12	s071G06	E018K21	Het	8.8	0.0	0.3	0.2[5]
3	12-2	H028L03	s042B23	Het	8.8	0.0	0.3	0.0[5]
3	2-10	H030A19	H011C02	Border	5.1	0.2	0.8	0.6[5]
3	10-11	H001H05	M003H09	Eu	1.5	0.2	0.3	0.0
3	11-3	E031G05	M015G06	Eu	1.5	0.4	0.6	0.1
3	3-13	s048H23	E006F17	Eu	1.5	0.3	0.4	0.1
4	1-3	H012B20	E036C15	Het	8.8	0.06	0.5	0.3
4	3-2	H018I03	M036H17	Cent	3.3	0.2	0.6	0.4
4	2-4	M016J15	M038M16	Het	8.8	0.03	0.2	0.1
4	4-5	M029O16	H013P02	Eu	1.5	0.04	0.1	0.0
4	5-6	M018D12	H020C13	Chrom	8.8	0.2	1.9	1.6
5	1-2	H002J04	s031L05	Het	8.8	0.1	1.1	0.9
5	2-3	s084H11	H016D20	Het	8.8	0.1	1.1	0.8

6	1-3	H028K02	H025N04	Het	8.8	0.3	2.8	2.4
6	3-2	M029F23	E040C12	Het	8.8	0.1	0.8	0.6
6	2-4	E013C07	M029C07	Het	8.8	0.1	0.6	0.4
6	4-5	E009J17	E033O20	Border	5.1	0.0	0.1	0.0
6	5-6	H031J01	M034N10	Border	5.1	0.0	0.1	0.0
6	6-7	E023E10	E018M04	Eu	1.5	0.3	0.4	0.0
6	7-8	E008B16	H025F06	Eu	1.5	0.2	0.3	0.0
7	1-2	M016H01	H032J14	Het	8.8	0.06	0.5	0.4
7	2-3	E016C19	H001O15	Het	8.8	0.3	2.5	2.4
7	3-4	H024C01	E032D11	Eu	1.5	0.13	0.2	0.0
8	1-2	H008J16	H015E06	Eu	1.5	0.3	0.4	0.0
8	2-7	H009K19	M012I24	Border	5.1	0.2	0.8	0.5
8	7-5	M012I24	s093G06	Border	5.1	0.1	0.6	0.3
8	5-6	s030B23	s105O13	Border	5.1	0.04	0.2	0.0
8	6-3	M005G10	s078C24	Het	8.8	0.04	0.3	0.05
8	3-4	s058J24	E024O12	Het	8.8	0.1	1.2	1.0
8	4-8	M019D14	H033A16	Het	8.8	0.2	1.4	0.8
8	8-9	M007C12	s082B11	Eu	1.5	0.2	0.3	0.0
9	1-4	M025N23	E015A15	Het	8.8	0.2	1.7	1.5
9	4-3	E025E12	E018E08	Het	8.8	0.1	0.9	0.8
9	3-5	M005G01	M013P10	Het	8.8	0.2	1.4	1.1
9	5-6	E022O16	M018I03	Het	8.8	0.2	1.4	1.3
9	6-2	H005M15	F051C04	Eu	1.5	0.2	0.3	0.0
9	2-7	F051C04	M019G06	Eu	1.5	0.1	0.1	0.0
9	7-8	M020M04	M025N11	Eu	1.5	0.4	0.5	0.0
9	8-9	H010F04	H001I06	Eu	1.5	0.1	0.2	0.0
9	9-10	E024O21	M037I08	Eu	1.5	0.4	0.5	0.1
10	1-2	E009D07	H023E16	Border	5.1	0.02	0.1	0.0
10	2-4	s071N16	E029F05	Het	8.8	0.1	0.7	0.4
10	4-3	M011N01	s042K13	Het	8.8	0.02	0.2	0.0
10	3-5	s121P17	F014E24	Eu	1.5	0.13	0.2	0.1
10	5-6	F014E24	E036N16	Eu	1.5	0.10	0.2	0.0
11	1-4	E018D15	H008F06	Het	8.8	0.1	0.7	0.4
11	4-2	H004F15	s082L15	Het	8.8	0.1	0.9	0.6
11	2-3	H001N12	E031L09	Het	8.8	0.2	1.8	1.4
11	3-5	s045H10	s084O21	Border	5.1	0.1	0.5	0.1
11	5-6	s017L16	s105E01	Border	5.1	0.2	0.9	0.7
12	1-8	E005K14	H016B06	Border	5.1	0.1	0.4	0.2
12	8-7	M038L04	E036O23	Border	5.1	0.0	0.2	0.0

12	7-2	E003D15	M008A07	Border	5.1	0.1	0.4	0.1
12	2-6	H003H12	M023C14	Het	8.8	0.1	0.7	0.6
12	6-5	E004B02	M040D19	Het	8.8	0.1	0.8	0.5
12	5-3[6]	E012H19	H005D14	Het	8.8	0.1	1.0	0.8
12	3-4	M035N06	M006L17	Eu	1.5	0.1	0.1	0.0
12	4-9	H017P17	E031M18	Eu	1.5	0.6	0.9	0.1
12	9-10	E024H05	H029L21	Eu	1.5	0.1	0.2	0.0
							**Total:**	**43.9**

[1] Gaps are named by the two flanking scaffolds. Scaffold A is closer to the short arm of the chromosome, and across the gap, scaffold B is closer to the long arm of the chromosome. Scaffold number is based on scaffold order from head to tail of pseudomolecules based on the linkage map, and the number and name of each scaffold is defined in Table S1.

[2] BAC library names have been abbreviated. H=HindIII, E=EcoRI, M=MboI, s=sheared, F=Fosmid

[3]Eu=Euchromatin, Het=Pericentric Heterochromatin, Cent=Centromere, Chrom=Chromomere (non-pericentric heterochromatin), Border=in transition from euchromatin to heterochromatin. These landmarks are based on chromosome structure in SC spreads.

[4] Corrected gap size estimates are smaller than distance between BACs because BACs were not usually at the absolute end of the scaffold and FISH positions were calculated from the middle of the BAC rather than the end of the BAC. See Materials and Methods for procedure to correct gap size. Negative values have been changed to 0.

[5] Positions of the tail of scaffold 8, both ends of scaffolds 2 and 12, and the head of scaffold 10 are relatively uncertain, so estimates of the sizes of gaps 8-12, 12-2, and 2-10 are relatively uncertain as well.

[6] Tomato variety Heinz 1706 was used for measurements of this gap due to an inversion of the kinetochore compared to Cherry Tomato LA4444.
