# Supplementary material for: Fluorescence In Situ Hybridization and Optical Mapping to Correct Scaffold Arrangement in the Tomato Genome
Source: G3 (Bethesda). 2014 May 30;4(8):1395–405. doi: 10.1534/g3.114.011197 (PMC4132171; doi:10.1534/g3.114.011197)
Supplement: Supporting Information [file supp_g3.114.011197_TableS8.pdf]

Table S8 Comparison of gap sizes by optical mapping and FISH

Adjacent Scaffolds		FISH Gap ID	Optical Gap Size (Kb)	FISH Gap Size (Kb)	Difference in Gap Size (FISH minus Optical) Kb[1]	Chromatin Type[3]
SL2.40SC05611	SL2.40SC05380	Chr. 12: 9-10	0	0[2]	0	Eu
SL2.40SC03622	SL2.40SC05054	Chr. 6: 7-8	1	0[2]	-1	Eu
SL2.40SC05732	SL2.40SC05054	Chr. 6: 6-7	-2	0[2]	2	Eu
SL2.40SC03798	SL2.40SC05925	Chr. 10: 1-2	2	0[2]	-2	Border
SL2.40SC05188	SL2.40SC05732	Chr. 6: 5-6	15	0[2]	-15	Het
SL2.40SC04813	SL2.40SC03770	Chr. 8: 1-2	19	0[2]	-19	Eu
SL2.40SC03835	SL2.40SC04236	Chr. 8: 6-3	23	50	27	Het
SL2.40SC03852	SL2.40SC04828	Chr. 9: 8-9	46	0[2]	-46	Eu
SL2.40SC03701	SL2.40SC06911	Chr. 3: 3-13	37	100	63	Eu
SL2.40SC04828	SL2.40SC06214	Chr. 9: 9-10	26	100	74	Eu
SL2.40SC04236	SL2.40SC03749	Chr. 8: 5-6	76	0[2]	-76	Het
SL2.40SC04915	SL2.40SC05611	Chr. 12: 4-9	16	100	84	Eu
SL2.40SC06917	SL2.40SC06903	Chr. 1: 8-7	33	200	167	Eu
SL2.40SC05941	SL2.40SC06917	Chr. 1: 6-8	68	300	232	Eu/Border
SL2.40SC03594	SL2.40SC03666	Chr. 1: 2-4	347	600	253	Het
SL2.40SC05941	SL2.40SC05010	Chr. 1: 5-6	7	300	293	Border
SL2.40SC04208	SL2.40SC04732	Chr. 2: 4-6	65	400	335	Het
SL2.40SC03731	SL2.40SC05397	Chr. 7: 1-2	48	400	352	Het
SL2.40SC06137	SL2.40SC03876	Chr. 11: 5-6	41	700	659	Eu
SL2.40SC04950	SL2.40SC04008	Chr. 9: 4-3	124	800	676	Het
SL2.40SC04757	SL2.40SC04057	Chr. 12: 5-3	42	800	758	Het
SL2.40SC04701	SL2.40SC03835	Chr. 8: 3-4	32	1000	968	Het
SL2.40SC04785	SL2.40SC04950	Chr. 9: 3-5	18	1100	1082	Het
SL2.40SC04785	SL2.40SC04777	Chr. 9: 5-6	139	1300	1161	Het
		**Totals:**	**1223**	**8250**	**7027**	

[1] The gap sizes are arranged in order of increasing absolute difference in gap size estimates. Gaps in euchromatin tend to be smaller than gaps in heterochromatin.

[2] A value of 0 kb indicates that a gap had a negative value after correcting for the position of FISH signals relative to the end of the scaffold (see Estimating Gap Sizes in Materials and Methods and in Results).

[3] Eu = euchromatin, Het = heterochromatin, Border = transition zone between euchromatin and heterochromatin
