# Supplementary material for: Fluorescence In Situ Hybridization and Optical Mapping to Correct Scaffold Arrangement in the Tomato Genome
Source: G3 (Bethesda). 2014 May 30;4(8):1395–405. doi: 10.1534/g3.114.011197 (PMC4132171; doi:10.1534/g3.114.011197)
Supplement: Supporting Information [file supp_g3.114.011197_TableS9.pdf]

Table S9 Estimates of tomato genome size (1C DNA amount)

Method	Genome Size pg (Mb equivalent)[1]	Reference
Microspectrophotometry	1.0 (965)	(Bennett & Smith 1976)
Microspectrophotometry and flow cytometry	0.95 (917)	(Michaelson *et al.* 1991)
Flow cytometry	0.75 (724)	(Galbraith *et al.* 1983)
Flow cytometry	1.10 (1062)	(Anderson *et al.* 1985)
Flow cytometry	1.0 (965)	(Arumuganathan & Earle 1991)
Flow cytometry	0.98 (946)	(Dolezel *et al.* 1992)
Flow cytometry	0.93 (897)	(Valkonen 1994)
Flow cytometry	1.00 (965)	(Obermayer *et al.* 2002)
Cot curve	0.86 (830)	(Peterson *et al.* 1998)
**Average**	**0.95 (919)**	

[1] Picograms were converted to megabase pairs (Mb) by multiplying the number of picograms (pg) times 965 Mb (Bennett & Smith 1976).

Anderson, L. K., S. M. Stack, M. H. Fox, and C. Zhang, 1985 The relationship between genome size and synaptonemal complex length in higher plants. Exp. Cell Res. **156:** 367-378.

Arumuganathan, K., and E. D. Earle, 1991 Nuclear DNA content of some important plant species. Plant Mol. Biol. Reptr. **9:** 208-218.

Bennett, M. D., and J. B. Smith, 1976 Nuclear DNA amounts in angiosperms. Phil. Trans. Roy. Soc. Lond. B **274:** 227-274.

Dolezel, J., S. Sgorbati, and S. Lucretti, 1992 Comparison of three DNA fluorochromes for flow cytometric estimation of nuclear DNA content in plants. Physiol. Plant. **85:** 625-631.

Galbraith, D. W., K. R. Harkins, J. M. Maddox, N. M. Ayers, D. P. Sharma *et al.* 1983 Rapid flow cytometric analysis of the cell cycle in intact plant tissues. Science **220:** 1049-1051.

Michaelson, M. J., H. J. Price, J. R. Ellison, and J. S. Johnston, 1991 Comparison of plant DNA contents determined by Feulgen microspectrophotometry and laser flow cytometry. Am. J. Bot. **78:** 183-188.

Obermayer, R., I. J. Leitch, L. Hanson, and M. D. Bennett, 2002 Nuclear DNA C-values in 30 species double the familial representation in pteridophytes. Ann. Bot. **90:** 209-217.

Peterson, D. G., W. R. Pearson, and S. M. Stack, 1998 Characterization of the tomato (*Lycopersicon esculentum*) genome using in vitro and in situ DNA reassociation. Genome **41:** 346-356.

Valkonen, J. P. T., 1994 Nuclear DNA content of the Solanum spp. in the series Etuberosa as determined by laser flow cytometry. Annals of Applied Botany **125:** 589-600.
