# Supplementary material for: Fluorescence In Situ Hybridization and Optical Mapping to Correct Scaffold Arrangement in the Tomato Genome
Source: G3 (Bethesda). 2014 May 30;4(8):1395–405. doi: 10.1534/g3.114.011197 (PMC4132171; doi:10.1534/g3.114.011197)
Supplement: Supporting Information [file supp_g3.114.011197_TableS10.pdf]

Table S10 Location of unassigned (chromosome *0*) BACs by FISH

Library	BAC ID	Chromosome	Arm	% from KC	Gap ID[1]	Flanking Scaffold	Flanking Scaffold
Mbo I	014K08	1	S	0.6%	3-2	SL2.40sc04191	SL2.40sc03666
Mbo I	056H03	2	S	8.3%	1-2	SL2.40sc06593	SL2.40sc04142
Mbo I	063O23	2	S	8.1%	1-2	SL2.40sc06593	SL2.40sc04142
Mbo I	016G20	2	S	7.7%	1-2	SL2.40sc06593	SL2.40sc04142
Mbo I	025G13	2	S	7.7%	1-2	SL2.40sc06593	SL2.40sc04142
Hind III	078N01	2	S	7.3%	1-2	SL2.40sc06593	SL2.40sc04142
EcoR I	040P16	2	S	6.8%	1-2	SL2.40sc06593	SL2.40sc04142
Hind III	009K14	2	S	6.7%	1-2	SL2.40sc06593	SL2.40sc04142
EcoR I	024C17	2	S	6.6%	1-2	SL2.40sc06593	SL2.40sc04142
Mbo I	034C01	2	S	6.2%	1-2	SL2.40sc06593	SL2.40sc04142
Mbo I	050I21	2	S	6.0%	1-2	SL2.40sc06593	SL2.40sc04142
EcoR I	031O09	2	S	5.9%	1-2	SL2.40sc06593	SL2.40sc04142
Mbo I	004J02	2	S	5.9%	1-2	SL2.40sc06593	SL2.40sc04142
Mbo I	034J07	2	S	5.9%	1-2	SL2.40sc06593	SL2.40sc04142
Hind III	058D23	2	S	5.7%	1-2	SL2.40sc06593	SL2.40sc04142
Hind III	043D06	2	S	5.6%	1-2	SL2.40sc06593	SL2.40sc04142
Hind III	012G12	2	S	5.3%	1-2	SL2.40sc06593	SL2.40sc04142
Hind III	060L16	2	S	5.3%	1-2	SL2.40sc06593	SL2.40sc04142
Mbo I	010K24	2	L	16.9%	3-7	SL2.40sc03766	SL2.40sc03665
EcoR I	032D02	2	L	17.0%	3-7	SL2.40sc03766	SL2.40sc03665
Mbo I	050I17	2	L	17.3%	3-7	SL2.40sc03766	SL2.40sc03665
EcoR I	002O15	2	L	17.3%	3-7	SL2.40sc03766	SL2.40sc03665
Hind III	060D16	2	L	17.4%	3-7	SL2.40sc03766	SL2.40sc03665
Mbo I	029F22	2	L	17.5%	3-7	SL2.40sc03766	SL2.40sc03665
Hind III	059K12	2	L	17.9%	3-7	SL2.40sc03766	SL2.40sc03665
Hind III	026M09	2	L	17.9%	3-7	SL2.40sc03766	SL2.40sc03665
EcoR I	042F18	2	L	18.1%	3-7	SL2.40sc03766	SL2.40sc03665
EcoR I	006L04	2	L	18.1%	3-7	SL2.40sc03766	SL2.40sc03665
Hind III	027N03	2	L	18.2%	3-7	SL2.40sc03766	SL2.40sc03665
EcoR I	007J10	2	L	18.3%	3-7	SL2.40sc03766	SL2.40sc03665
EcoR I	039I06	2	L	18.8%	3-7	SL2.40sc03766	SL2.40sc03665
Hind III	067G24	2	L	18.9%	3-7	SL2.40sc03766	SL2.40sc03665
EcoR I	027P01	4	L	0.3%	3-2	SL2.40sc05339	SL2.40sc03683
EcoR I	056L08	6	S	32.6%	1-3	SL2.40sc04474	SL2.40sc06140
EcoR I	047D19	6	S	31.7%	1-3	SL2.40sc04474	SL2.40sc06140

EcoR I	005K11	6	S	30.9%	1-3	SL2.40sc04474	SL2.40sc06140
EcoR I	031A01	6	S	30.8%	1-3	SL2.40sc04474	SL2.40sc06140
EcoR I	035D19	6	S	30.5%	1-3	SL2.40sc04474	SL2.40sc06140
Mbo I	049I18	6	S	29.8%	1-3	SL2.40sc04474	SL2.40sc06140
Hind III	029E01	6	S	29.6%	1-3	SL2.40sc04474	SL2.40sc06140
Hind III	027D17	6	S	28.9%	1-3	SL2.40sc04474	SL2.40sc06140
Hind III	078K13	6	S	28.7%	1-3	SL2.40sc04474	SL2.40sc06140
Hind III	002P09	6	S	28.6%	1-3	SL2.40sc04474	SL2.40sc06140
Mbo I	016K18	6	S	28.1%	1-3	SL2.40sc04474	SL2.40sc06140
EcoR I	021L01	6	S	27.6%	1-3	SL2.40sc04474	SL2.40sc06140
Hind III	001E08	6	S	27.3%	1-3	SL2.40sc04474	SL2.40sc06140
EcoR I	013G12	6	S	25.8%	1-3	SL2.40sc04474	SL2.40sc06140
EcoR I	025E06	7	S	57.1%	n/a[2]	n/a[2]	n/a[2]
Hind III	003K20	7	L	13.6%	n/a[2]	n/a[2]	n/a[2]
Hind III	027F11	7	L	19.3%	2-3	SL2.40sc05397	SL2.40sc03685
EcoR I	021M11	7	L	19.5%	2-3	SL2.40sc05397	SL2.40sc03685
EcoR I	041L20	7	L	19.9%	2-3	SL2.40sc05397	SL2.40sc03685
EcoR I	041L23	7	L	19.9%	2-3	SL2.40sc05397	SL2.40sc03685
Hind III	042K23	7	L	20.5%	2-3	SL2.40sc05397	SL2.40sc03685
Hind III	036D05	7	L	21.1%	2-3	SL2.40sc05397	SL2.40sc03685
Hind III	025A06	7	L	21.3%	2-3	SL2.40sc05397	SL2.40sc03685
Mbo I	017I04	7	L	21.5%	2-3	SL2.40sc05397	SL2.40sc03685
Mbo I	017P20	7	L	21.5%	2-3	SL2.40sc05397	SL2.40sc03685
Hind III	012C17	7	L	23.1%	2-3	SL2.40sc05397	SL2.40sc03685
EcoR I	002D11	8	S	36.5%	7-5	SL2.40sc04167	SL2.40sc03749
Hind III	041G04	8	S	35.7%	7-5	SL2.40sc04167	SL2.40sc03749
Mbo I	041D13	8	S	34.9%	7-5	SL2.40sc04167	SL2.40sc03749
EcoR I	008F12	8	L	3.1%	n/a[2]	n/a[2]	n/a[2]
Mbo I	056H18	9	S	9.4%	1-4	SL2.40sc03771	SL2.40sc04008
Hind III	072H18	9	S	6.6%	1-4	SL2.40sc03771	SL2.40sc04008
Mbo I	045G03	9	S	3.4%	1-4	SL2.40sc03771	SL2.40sc04008
EcoR I	052D12	9	S	3.4%	1-4	SL2.40sc03771	SL2.40sc04008
Mbo I	042F10	9	L	99.6%	10-T	SL2.40sc06214	telomere
EcoR I	045G19	11	L	8.0%	n/a[2]	n/a[2]	n/a[2]
Mbo I	047F15	11	L	14.3%	2-3	SL2.40sc04054	SL2.40sc03752
Hind III	062E02	11	L	15.2%	2-3	SL2.40sc04054	SL2.40sc03752
Mbo I	050G12	12	S	36.9%	1-8	SL2.40sc04607	SL2.40sc06147
EcoR I	010P04	12	S	35.8%	8-7	SL2.40sc06147	SL2.40sc04039
Hind III	006G23	12	L	23.6%	n/a[2]	n/a[2]	n/a[2]

EcoR I	045D23	12	L	81.0%	9-10	SL2.40sc05611	SL2.40sc05380

[1]Gap ID refers to the gap name given in the FISH Scaffold/Gap diagrams and is derived from the linkage map-based order of the scaffolds.

[2] BAC is not within a gap
